# Combining Spatial Multi‐Omics Data to Decipher Spatial Domains and Elucidate Cell Heterogeneity Based on Self‐Supervised Graph Learning

**DOI:** 10.1002/advs.75533

**Published:** 2026-05-11

**Authors:** Yuejing Lu, Rui Qiao, Ying Li, Junhong Li, Nuoya Yue, Jing Ge, Jiao Wang, Luonan Chen, Peiluan Li

**Affiliations:** ^1^ School of Mathematics and Statistics Henan University of Science and Technology Luoyang China; ^2^ School of Artificial Intelligence Wuhan University Wuhan China; ^3^ College of Electronic and Information Engineering Shenzhen University Shenzhen Guangdong China; ^4^ School of Life Sciences Shanghai University Shanghai China; ^5^ Shanghai Immune Therapy Institute Renji Hospital, Shanghai Jiao Tong University School of Medicine Shanghai China; ^6^ School of Life Science and Technology Shandong Vocational University of Foreign Affairs Jinan China; ^7^ Department of Rehabilitation Medicine The Affiliated Taian City Central Hospital of Qingdao University Shandong China; ^8^ Key Laboratory of Biotechnology and Bioengineering of State Ethnic Affairs Commission Northwest Minzu University Lanzhou Gansu China; ^9^ School of Mathematical Sciences and School of AI Shanghai Jiao Tong University Shanghai China; ^10^ Tianfu Jincheng Laboratory Chengdu China; ^11^ Key Laboratory of Systems Health Science of Zhejiang Province, Hangzhou Institute for Advanced Study University of Chinese Academy of Sciences Hangzhou China; ^12^ Guangdong Provincial Key Laboratory of Mathematical and Neural Dynamical Systems Great Bay University Dongguan China

**Keywords:** cancer microenvironments, decipher spatial domains, self‐supervised graph learning by self‐training, spatial multi‐omics, tumor heterogeneity

## Abstract

Spatial multi‐omics technologies enable in situ molecular profiling but face challenges in integrating multi‐modal data for spatial domain identification and cell heterogeneity analysis. We propose SOTMGF, a self‐supervised, goal‐directed multi‐view graph fusion framework for spatial multi‐omics data. SOTMGF includes five modules: pre‐clustering, sparse feature processing, multi‐view feature extraction and fusion (integrating molecular expression, spatial location, disease microenvironment, and molecular associations), and multi‐modality integration. The self‐training process and graph embedding are optimized iteratively within a unified framework, enabling mutual benefits across several components. SOTMGF outperformed existing methods in spatial domain identification, data denoising, and detection of spatially variable molecular features. Innovatively, it jointly analyzes spatial transcriptomics (ST) and proteomics (SP) from the same tissue, computationally generates spatial ATAC‐seq via Tangram, reconstructs spatial pseudo‐expression to identify spatial dark genes/proteins (SDGs/SDPs), and iteratively optimizes self‐training and graph embedding in a unified framework. SOTMGF outperforms existing methods in spatial domain detection and denoising, reveals mRNA‐protein discordance, predicts key transcription factors, and aids biomarker and therapeutic target discovery, advancing spatial biology research, molecular regulatory mechanisms, and therapeutic discovery.

## Graphical abstract

A self‐supervised multi‐view graph fusion framework integrates spatial multi‐omics, excelling in domain identification and denoising. It reconstructs spatial pseudo‐expression, jointly analyzes multi‐omics data, infers RNA velocity, predicts spatial omics features from single‐cell multi‐omics, and detects spatially dark genes and transcription factors, advancing spatial biology and therapeutic discovery.

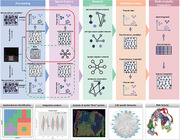

## 1 Introduction

Spatial omics, which involves the in situ measurement of molecular parameters on intact tissue samples [1], reveals intercellular microenvironmental interactions and spatial heterogeneity from a molecular perspective. Various spatial omics techniques have been developed, including spatial transcriptomics (ST), spatial proteomics (SP), and spatial metabolomics. These advances provide enriched biomolecular profiles for each observation unit (e.g., spots, pixels, or single cells) along with corresponding spatial location information, enabling a deeper understanding of the interactions and spatial distribution of molecules within living organisms [2, 3].

ST technologies encompass both in situ capture and sequencing‐based methods, such as 10x Genomics Visium and Slide‐seq, as well as image‐based technologies, such as STARmap. These technologies play a crucial role in discovering novel biomarkers, dissecting cell heterogeneity, and evaluating therapeutic responses [4]. Several computational methods have been developed to analyze such complex data. For instance, STAGATE is a graph attention autoencoder framework to accurately identify spatial domains in ST data [5]. Similarly, SpatialFlow generates spatially consistent low‐dimensional embeddings through a spatially regularized network of deep graphs, effectively integrating gene expression similarity with spatial information [6]. Meanwhile, conST offers interpretability by identifying representative points that support clustering [7]. DeepST provides a versatile and precise deep learning framework capable of integrating ST data across multiple batches or technological platforms to improve spatial domain identification [8]. Additionally, stMGATF integrates multi‐modality information to elucidate tissue heterogeneity and detect spatial dark genes (SDGs) that may play significant roles in disease progression [9]. Together, these tools have significantly advanced the field of ST data analysis and delivered notable successes. Nevertheless, current methods still face challenges in optimizing the self‐training processes and embedding extraction within a unified iterative framework. Moreover, there remains a need for more effective strategies to integrate multi‐omics and multi‐modality information, including molecular associations.

Spatial multi‐omics provides complementary information that significantly enhances our understanding of complex biological processes. Notably, since proteins are key functional drivers in biology and represent common therapeutic targets, SP enables the simultaneous quantification of multiple proteins while preserving spatial information at the subcellular level [10, 11]. Several computational tools have been developed for analyzing spatial omics data. For example, Squidpy aggregates tools from omics and image analysis to facilitate scalable characterization of spatial molecular profiles [12]. Similarly, SOTIP offers a general approach that incorporates tumor microenvironments and their interrelationships within a unified graph, supporting various downstream analytical tasks [13]. Despite these advances, current methods are predominantly designed for single‐omics datasets and lack the capability to jointly analyze multiple spatial omics modalities from the same tissue section. Furthermore, most spatial sequencing technologies are limited to measuring only one type of omics data along with spatial coordinates, thereby missing critical correlative information from other molecular layers.

In recent years, there has been a growing emphasis on developing computational approaches that utilize multi‐omics and multi‐modality information, such as molecular expression, physical location, cellular microenvironment, and molecular association, to enable integrated analysis of spatial multi‐omics, including SP and ST within a single tissue section [14]. Several methods have been introduced for the joint analysis of multi‐modality spatial omics. For instance, SpatialGlue employs a dual‐attention mechanism operating at both the intra‐modal and inter‐modal levels, accounting for the fact that different graphs may contribute variably to each spot and that distinct omics modalities offer complementary biological insights [15]. Similarly, MISO (Multi‐modal Spatial Omics) is a computational framework for feature extraction and spatial clustering, aiming to achieve comprehensive integration across diverse modalities in multi‐modality spatial experiments, including omics data and high‐resolution histology images [16]. However, current methods often overlook critical aspects of the spatial microenvironment surrounding each spot and fail to account for molecular association networks. These elements could play essential roles in accurately identifying spatial domains and elucidating tissue organization, suggesting a significant gap in existing integrative models.

To address the aforementioned challenges, we propose SOTMGF, a self‐supervised, goal‐directed multi‐view graph fusion framework, designed to integrate multi‐modality spatial omics data, including molecular expression (proteome, transcriptome, and epigenome), spatial location, tumor microenvironment information, and molecular associations.

In contrast to existing methods, SOTMGF employs a self‐training graph clustering mechanism guided by automatically generated pseudo‐labels, through which it refines clustering outcomes and mutual optimization between modules. This process ensures collaborative learning across modalities and produces informative, low‐dimensional embeddings conducive to downstream tasks. By effectively preserving the multi‐modality information inherent in spatial omics data, SOTMGF enables the derivation of biologically meaningful representations that reveal cellular developmental trajectories within spatial contexts and facilitate the identification of novel markers critical for understanding diverse disease progression.

SOTMGF demonstrates strong performance in denoising raw spatial data, detecting spatial structural domains, and identifying cancer‐enriched structural regions, which aids in elucidating tissue heterogeneity. SOTMGF supports integration of ST, SP, and paired scRNA‐seq data, leveraging cross‐modal complementarity to uncover substantial discordances between mRNA and protein expression. To further extend the utility of SOTMGF, we computationally imputed spatial ATAC‐seq data from mouse brain MERFISH ST and matching scSHARE‐seq data using Tangram. This approach allows integrative multi‐omics analysis incorporating spatial transcriptomics, chromatin accessibility, and RNA expression—facilitating biomarker discovery and nomination of potential therapeutic targets. Most importantly, a key innovation of SOTMGF is its incorporation of molecular interaction networks to reconstruct spatial pseudo‐expression (SPE). This enables identification of novel biomarkers that, despite showing no significant difference in raw expression, exhibit pronounced spatial variation in SPE. Additionally, SOTMGF predicts spatially dark genes (SDGs), spatial dark proteins (SDPs), and regulatory transcription factors (TFs) implicated in the disease samples. These factors likely operate through functional and signaling pathways critical to various diseases, offering insights not only into the mechanistic underpinnings of the diseases but also into early clinical diagnosis and targeted therapy.

In summary, we proposed SOTMGF, a self‐supervised, goal‐directed, and self‐training multi‐view graph learning model, which was designed to integrate spatial multi‐omics data. This flexible framework effectively deciphers spatial domains and tumor heterogeneity, providing a powerful tool for multidimensional spatial biology research. Its applications in identifying critical spatial features, such as domain boundaries, molecular dark matter, and cell‐state transitions, highlight its potential to advance both basic biological discovery and clinical translation.

## 2 Results

### 2.1 Overview of the SOTMGF Framework and its Enhancement of Spatial Domain Detection in Simulated Data and Mouse Spleen Dataset

**FIGURE 1:**
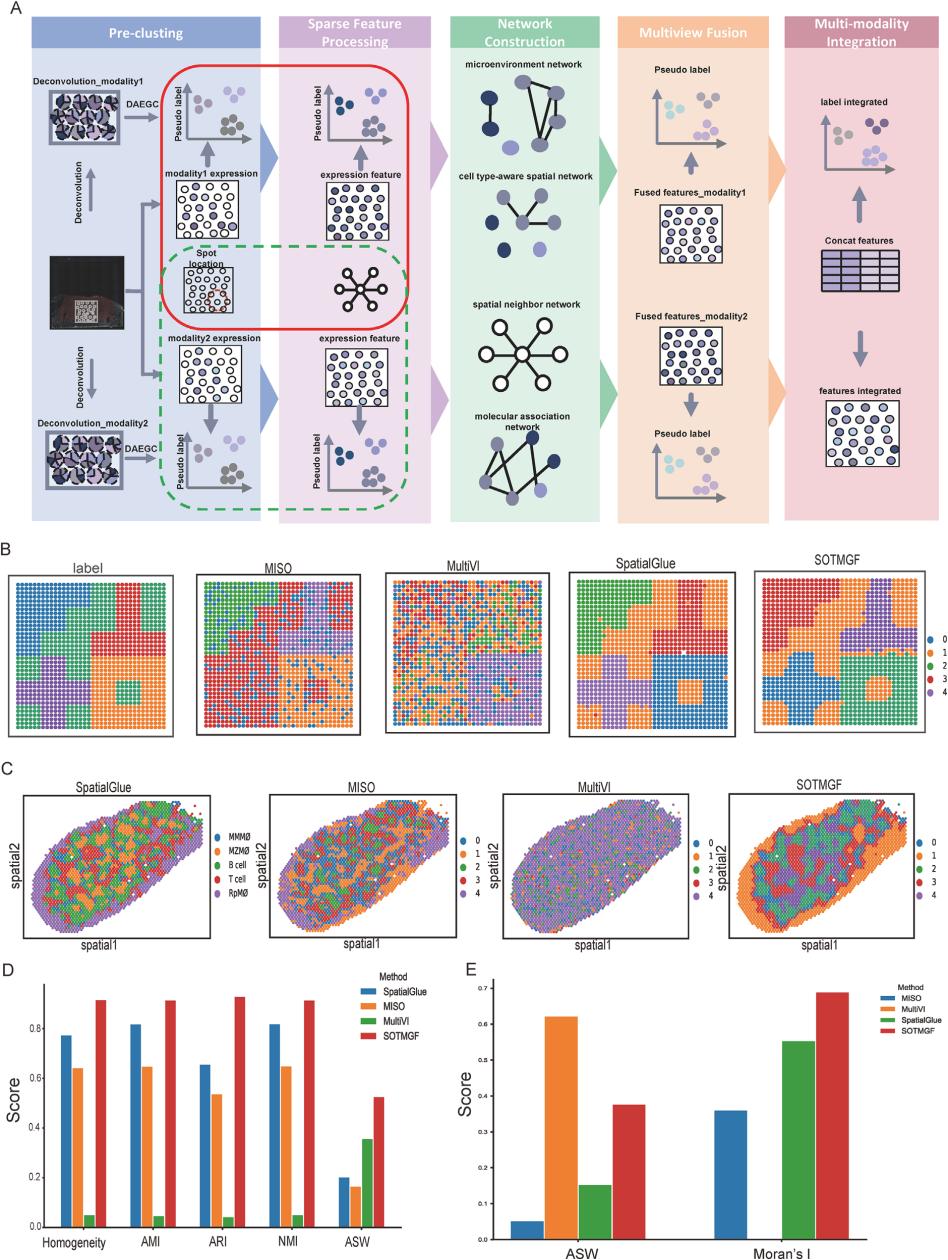
Overview of the SOTMGF model and benchmarking of SOTMGF and other methods on simulated and spleen datasets. (A) Schematic illustration of SOTMGF architecture. SOTMGF is divided into five parts: preclustering, sparse feature processing, network construction and multi‐view fusion, multi‐modality integration. The preclustering module preclustered based on DAEGC. The sparse feature processing module uses a Transformer to generate the dense gene expression features for re‐clustering to obtain the pseudo‐labels. The red rectangles represent the sparse feature processing module for modality 1, and the green rectangles represent the sparse feature processing module for modality 2. The multi‐view fusion module constructs a spatial neighbor network and a cell type‐aware spatial network, a molecular expression‐aware cell neighborhood graph. The multi‐view fusion module integrates the low‐dimensional features from four views (two or more modalities) through the attention mechanism, the low‐dimensional features of multiple views (modalities) are extracted by MDAGC and fused to obtain the joint features and perform clustering. The multi‐modality integration module integrates the low‐dimensional features from different modalities through an interpretable Transformer. The embedding learning and clustering results are finally optimized by unsupervised self‐training iterations. (B) Labeling of the simulated dataset and spatial domain identification in tri‐modality, including spatial transcriptome, spatial proteome, and ATAC data. (C) Clustering results obtained by single‐cell and spatial multi‐omics integration methods (SpatialGlue, MISO, MultiVI, COSMOS, and SOTMGF) applied to mouse spleen RNA and protein data acquired using SPOTS. (D) Comparison histogram of spatial domain metrics identified in datasets containing three spatial omics data. (E) Comparison histogram of spatial domain metrics identified in spatial map RNA and protein data (annotated results of mouse spleen acquired using SPOTS).

SOTMGF is a comprehensive computational framework designed for analyzing spatial multi‐omics data through a structured workflow comprising five integrated modules: preclustering, sparse feature processing, network construction, multi‐view fusion, and multi‐modality integration (Figure 1). The preclustering module decomposes the cell type composition of each observation unit (e.g., spots, pixels, or single cells) using deconvolution based on the conditional autoregressive model (CARD) [17]. These cell type compositions, along with their physical location information, are preclustered using Deep Attentional Embedded Graph Clustering (DAEGC), which encodes both topological and nodal features into a compact representation. The sparse feature processing module is designed to transfer the sparse gene expression features to a dense feature space. It uses pseudo‐labels, generated by preclustering, as target information to supervise the node embedding learning. This module employs a Transformer‐based multi‐head self‐attention mechanism to generate dense gene expression features, which are then re‐clustered to obtain the pseudo‐labels. In the network construction and feature extraction module, physical location data are used to construct a physical location‐based spatial neighbor network and a cell type‐aware spatial network, which are pruned using pseudo‐labels. Additionally, it calculates the optimal transport distance to construct a microenvironment network by leveraging the distribution of neighboring cell types and combining it with molecular association network information. Furthermore, the low‐dimensional features of each view are extracted using Deep Attention‐Guided Clustering with dual self‐supervision (DAGC), which combines a soft self‐supervision strategy to preserve distributional consistency with a hard self‐supervision strategy with pseudo‐supervisory loss to improve clustering performance. The multi‐view fusion module and multi‐modality integration module integrate the low‐dimensional features from four views via an attention mechanism, yielding joint feature for clustering. The embedding learning and clustering results are finally optimized by unsupervised self‐training iterations. The self‐training process and graph embedding are co‐learned and jointly optimized within a unified framework, enabling several components to benefit mutually. Finally, we utilized raw modality 1 expression and raw modality 2 combined with physical location information to obtain low‐dimensional embeddings of modality 1 and modality 2 through SOTMGF. We then clustered the spatial barcodes into phenotypically distinct groups. Following this step, we concatenated the modality1 and modality 2 features and used the joint features as input to an interpretable Transformer model to learn the joint features for clustering.

To evaluate the performance of SOTMGF in spatial domain identification, we employed simulated spatial multi‐omics datasets generated by SpatialGlue using non‐negative spatial factorization [15]. SpatialGlue utilized the “ggblocks” model to construct expression matrices across distinct modalities: Modality 1 represented a spatial transcriptomic matrix (1296 cells × 1000 genes) following a zero‐inflated negative binomial (ZINB) distribution, while Modality 2 simulated spatial proteomic data (1296 cells × 100 proteins) under a negative binomial distribution. To mitigate stochasticity, five simulated datasets with varying parameters were generated for the dual‐modality scenario. In addition, a tri‐modality dataset incorporating the transcriptomic, proteomic, and epigenomic features was created. Gaussian noise was incorporated into all modalities to mimic real‐world biological variability.

For benchmarking against SpatialGlue [15], MISO [16], and MultiVI [18], SOTMGF was applied to multi‐modality datasets with ground‐truth labels (Figure 1B). In dual‐modality datasets, which include transcriptome and proteome, SOTMGF and SpatialGlue demonstrated high concordance with ground‐truth spatial domains. In contrast, MISO partially recovered structures, leaving one domain unresolved, while MultiVI exhibited poor performance due to the neglect of the spatial information (Figure S1). In the analysis of tri‐modality datasets, which include transcriptome, proteome, and epigenome, SOTMGF retained robust domain recovery. However, both SpatialGlue and MISO were unable to fully resolve one domain, with MISO displaying substantial noise. Despite integrating tri‐modality data, MultiVI lacked spatial awareness and resulted in indistinct domain boundaries (Figure 1B).

In addition, to evaluate the performance of SOTMGF in spatial domain identification, we employed the annotation of the mouse spleen dataset generated by SpatialGlue as ground truth. SOTMGF demonstrated high concordance with spatial domains generated by SpatialGlue (Figure 1C). In contrast, MISO partially recovered structures, leaving one domain unresolved, while MultiVI did not capture coherent clusters. We further visualized the extracted features using UMAP, which demonstrated that low‐dimensional embeddings derived from SOTMGF clearly revealed different states of the tumor (Figure S2). In contrast, embeddings generated by MISO and other methods exhibited overlapping distributions.

Quantitative evaluation using supervised metrics, Homogeneity, Adjusted Mutual Information (AMI), Adjusted Rand Index (ARI), Normalized Mutual Information (NMI), and Average Silhouette Width (ASW) confirmed SOTMGF's superiority. In tri‐modality analysis, SOTMGF consistently outperformed all competitors, underscoring its robustness and accuracy in multi‐modal spatial domain identification (Figure 1D). To evaluate the contribution of SOTMGF's multi‐modal fusion module, we visualized the spatial domain identification results of RNA, proteomics, and epigenomics separately to demonstrate the effectiveness of the multi‐modal fusion module (Figure S3). In mouse spleen spatial profiling data, SOTMGF is on par with MISO in these supervised metrics, with ARI significantly higher than the other methods (Figure 1E).

### 2.2 SOTMGF can Perform Joint Analysis of the Spatial Transcriptome and Spatial Proteome, and Contributes to Recognizing Spatial Dark Proteins and Spatial Dark Genes

**FIGURE 2:**
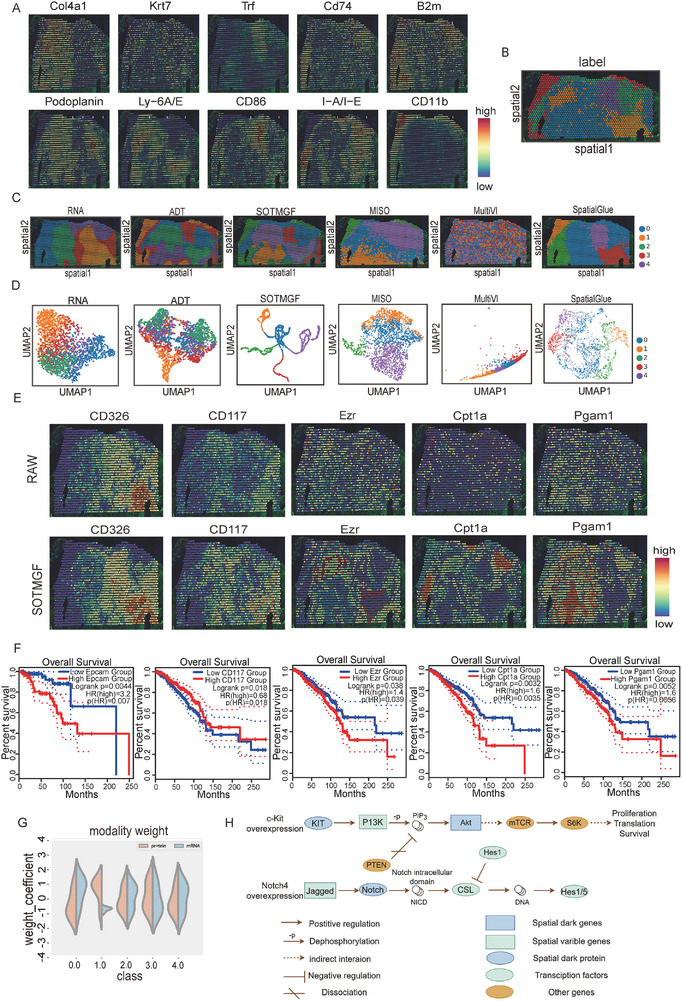
SOTMGF can perform joint analysis of the spatial transcriptome and spatial proteome. (A) Spatial patterns of potential genes and proteins specific to each structural domain. (B) Spatial barcode clustering results with annotation of major cell‐type enrichment based on SPOTS [11]. (C) Visualization of spatial barcode clustering results using ST, SP, the joint analysis of SP and ST, MISO, MultiVI, and SpatialGlue. (D) Scatter plot of the two‐dimensional UMAP extracted from latent features with ST, SP, and ST, SP, the joint analysis of SP and ST, MISO, MultiVI, and SpatialGlue. (E) Raw and denoised spatial expression patterns of representative SDGs and SDPs reconstructed by SOTMGF in a mouse breast cancer region, compared to raw expression values. (F) Prognostic significance of SOTMGF‐identified SDGs and SDPs based on survival analysis using TCGA breast cancer RNA‐seq data. (G) Modality weights coefficient explaining the importance of different modalities to each cluster in the mouse breast cancer dataset. (H) Schematic of NOTCH and RTK signaling pathways, highlighting crosstalk between key SDPs and TFs identified via SOTMGF.

To fully exploit the complementary information provided by proteomes and transcriptomes and deepen the understanding of complex biological processes, we propose SOTMGF. This method enables the joint analysis of SP and ST simultaneously and was validated using breast cancer spatial profiling data [19]. This dataset includes both protein and transcript measurements [11], which was generated using SPOTS with the 10x Visium technology, capturing whole transcriptomes and extracellular proteins with polyadenylated antibody‐derived tag‐conjugated antibodies. We visualized the spatial patterns of some potential genes and proteins specific to each structural domain as references (Figure 2A).

We employed the annotation of the mouse breast cancer dataset generated by SPOTS as ground truth (Figure 2B). Raw protein expression and raw gene expression, combined with physical location information, were used to obtain low‐dimensional embeddings of the proteome and transcriptome. Then, spatial barcodes were clustered into phenotypically distinct groups. Thereafter, the transcriptome and proteome features were concatenated, and the joint features were input into an interpretable Transformer model for clustering. The final clustering result was obtained, and the corresponding uniform manifold approximation and projection (UMAP) plot was drawn, clearly revealing the different states of the tumor (Figure 2C,D). It is apparent that SOTMGF is closest to the known layer [11] based on the spatial patterns of some potential genes and proteins specific to each structural domain, and the integration analysis of SP and ST achieved the best performance in spatial‐domain detection, as patches belonging to different layers are distinctly separated. UMAP plots generated from the potential features of SOTMGF showed that SOTMGF clearly distinguished different regions. The clusters were functionally annotated as follows: Cluster 0 (lymphocyte—enriched/peritumor), Cluster 1 (fibroblast—high), Cluster 2 (Mac2 ‐ enriched), Cluster 3 (Mac1 ‐ enriched), and Cluster 4 (fibroblast—low). Notably, M2 macrophages form an immunosuppressive barrier at the tumor border [11], and modality weight analysis indicated that transcriptomes contributed more to Clusters 0, 2, and 4, while proteomes dominated Clusters 1 and 3 (Figure 2G).

Traditional methods identify SDGs/SDPs based solely on raw expression, missing biologically relevant molecules with subtle but functionally significant changes. SOTMGF reconstructs spatial pseudo‐expression (SPE) by denoising raw data (weighted averaging of 15 nearest neighbors in low‐dimensional space), enabling the detection of SDGs/SDPs that are differential in SPE but not in raw data. Specifically, we first derived low‐dimensional embeddings of each cell using SOTMGF. We then computed Euclidean distances between these embeddings to identify the 15 nearest neighbors for each cell. By calculating the weighted average of their gene and protein expressions, we obtained reconstructed, denoised expression profiles. Utilizing these refined expressions, a set of robust SDPs and SDGs was successfully identified (Figure 2E; Figures S4 and S5). Notably, in a prognostic analysis using the TCGA Breast Invasive Carcinoma (BRCA) dataset, we confirmed that molecules such as CD326, CD117, and Cpt1a, which were selected via SOTMGF, were significantly associated with clinical outcomes in breast cancer (Figure 2F). For instance, the SDP CD117 (Epcam) showed a prognostic significance of p = 0.016, and the SDG Cpt1a yielded p = 0.0032. While their original spatial expressions showed no significant differences between cluster 0 and other clusters (Figure 2E, upper panel), the reconstructed SPE profiles revealed statistically significant differential expression (Figure 2E, lower panel). These findings indicate that SOTMGF‐unveiled molecules play crucial roles in disease progression and highlight the utility of SOTMGF in detecting functionally relevant spatial biomarkers that would otherwise remain undetected.

Transcription factors (TFs) play pivotal roles in breast cancer progression by regulating complex signaling networks [20]. Using SOTMGF‐reconstructed spatial expression profiles, we identified significant spatially dark genes (SDGs) and predicted their upstream TFs via ChEA3 [21] (Figure S6). Notably, five key TFs—Hes1, Rbpj, Irf1, Foxa1, and Jun—collectively regulated 75.37% of these SDGs (Figure S6). Among them, HES1 is known to induce Epithelial‐mesenchymal transition (EMT) in triple‐negative breast cancer (TNBC) and promote tumor invasion [22, 23], while RBPJ serves as a critical transcription factor within the Notch pathway, switching between repressor and activator roles based on Notch activation status [24]. Further functional and interaction analyses, including PPI network mapping (Figure S7) and pathway enrichment (Figure S8), revealed strong associations with breast cancer‐related processes and the Notch signaling pathway. Importantly, SOTMGF uncovered spatially segregated patterns of key molecules such as Notch4, overexpressed in TNBC vascular endothelial and tumor cells and linked to cancer stemness [25, 26], and CD117 (c‐Kit), which promotes metastasis in TNBC through activation of STAT3, Akt, and ERK1/2 [27]. These molecules operate within critical oncogenic pathways, including Notch and RTK, which regulate stemness, EMT, angiogenesis, and metastasis [28, 29, 30] (Figure 2H). These findings underscore the ability of SOTMGF to reveal functionally coherent yet spatially subtle regulatory programs, offering deeper biological insights inaccessible through conventional expression analysis.

### 2.3 SOTMGF Enhances Spatial Domain Detection in the Human Dorsolateral Prefrontal Cortex Dataset

**FIGURE 3:**
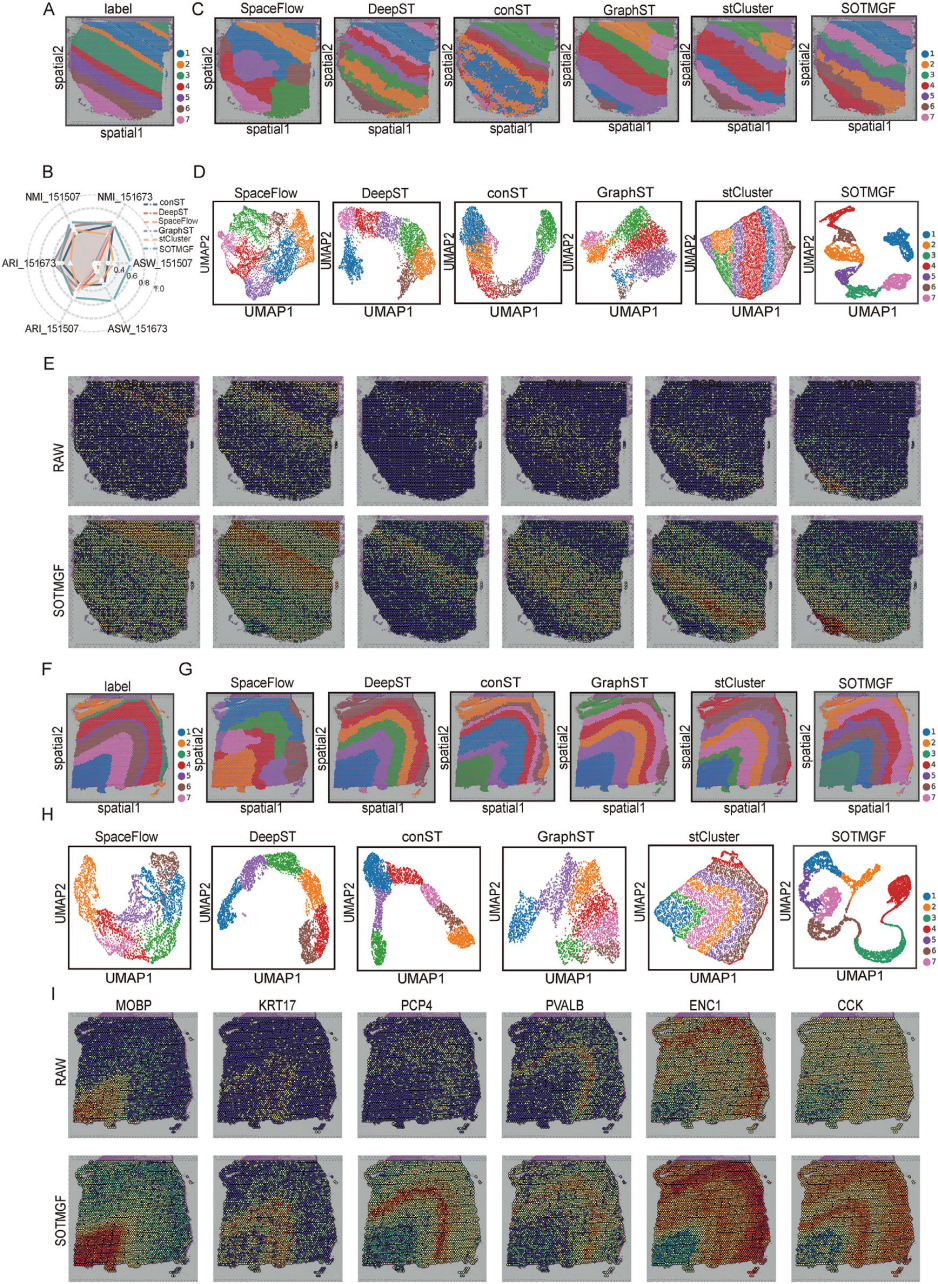
SOTMGF improves spatial domain detection in the human DLPFC dataset. (A) Ground‐truth segmentation of cortical layers and white matter (WM) in DLPFC section 151507. (B) Performance comparison of SOTMGF and other methods (SpaceFlow, DeepST, conST, GraphST, stCluster) based on ARI, NMI, and ASW metrics across slices 151507 and 151673. (C) Spatial domain segmentations of results of competing methods and SOTMGF for slice 151507. (D) Scatter plot of the two‐dimensional UMAP extracted from latent features by SpaceFlow, DeepST, conST, GraphST, stCluster, and SOTMGF in DLPFC section 151507. (E) Spatial expression of layer‐specific genes (*AQP4*, *HPCAL1*, *PCP4*, and *MOBP*) for slice 151507, denoised by SOTMGF, with raw data provided for comparison. (F) Ground‐truth segmentation of six cortical layers and WM in DLPFC section 151673. (G) Domain segmentations of cortical layers and WM by SpaceFlow, DeepST, conST, GraphST, stCluste, and SOTMGF in DLPFC section 151673. (H) Scatter plot of the two‐dimensional UMAP extracted from latent features by SpaceFlow, DeepST, conST, GraphST, stCluster, and SOTMGF in DLPFC section 151673. (I) Spatial expression of layer‐specific genes (*MOBP*, *KRT17*, *PCP4*, *PVALB*, *ENC1*, and *CCK*) for slice 151673, denoised by SOTMGF, with raw data provided for comparison.

To evaluate the generalizability and performance of SOTMGF on complex and well‐annotated brain structures, we applied the model to a human dorsolateral prefrontal cortex (DLPFC) 10X Visium dataset [31]. The dataset comprises 12 tissue slices from three human brains, with the six neuronal layers and white matter (WM) layer annotated as the ground truth for performance benchmarking. (Figure 3A).

Compared with previous spatial clustering methods, including SpaceFlow, DeepST, conST, GraphST, and stCluster, SOTMGF demonstrated improved identification of spatial domains. The adjusted Rand index [32] (ARI), normalized mutual information [33] (NMI), and average silhouette width [34] (ASW) index were used to evaluate performance. The radar charts summarized these metrics for the DLPFC 151507 and 151673 slices, and showed that SOTMGF performs well in spatial‐domain recognition based on these indices (Figure 3B).

Initially, SOTMGF decomposed the cell type composition of each spot using deconvolution based on CARD [17] (Figure S9). Visualization of the clustering results on slice 151507 revealed that SOTMGF achieved the highest agreement with manual annotations (ARI = 0.5707), most closely matching the ground‐truth layer segmentation (Figure 3C). Notably, SOTMGF successfully delineated all cortical layers, including L1 and L2, which were consistently overlooked by other methods, and produced spatially continuous domains with clear boundaries (Figure 3C). In contrast, other methods exhibited fragmented domain boundaries, poor layer discrimination, and inconsistent clustering patterns.

We further visualized the extracted features using UMAP, demonstrating that low‐dimensional embeddings derived from SOTMGF clearly distinguished between layers (Figure 3D). In contrast, embeddings generated by SpaceFlow and other methods exhibited overlapping distributions and failed to achieve effective layer separation. Ablation studies further confirmed the critical role of SOTMGF's attention‐based fusion mechanism. Replacing this mechanism with simple mean pooling led to decreased accuracy and reduced boundary consistency. Although the model without the cell type‐aware spatial network still achieved a competitive ARI value of 0.6066, the complete four‐view integration consistently provided superior spatial continuity and higher biological coherence (Figure S10). Additionally, by aggregating signals from the 15 nearest neighbors in the embedding space, SOTMGF denoised the expression data. The resulting spatial pseudo‐expression (SPE) profiles enhanced layer‐specific signal detection, revealing stronger enrichment of key marker genes such as AQP4 in Layer 1, HPCAL1 in Layer 3, and MOBP in white matter compared to the original results (Figure 3E) [35]. These genes are known to be involved in water transport and neuroinflammation, neuronal calcium signaling, and myelin function, respectively, further illustrating that SOTMGF enables the uncovering of biological spatial patterns.

These findings were further validated using DLPFC slice 151673 (Figure 3F). SOTMGF also produced spatially coherent domains with sharp boundaries (Figure 3G), well‐defined layer separation in UMAP space (Figure 3H), and enhanced expression patterns for layer‐specific genes, including MOBP (WM) and PCP4 (L5) [35] (Figure 3I). These results collectively demonstrate that SOTMGF effectively reduces technical noise, clarifies spatial expression patterns, and significantly improves the accuracy of spatial domain detection.

To address geometric invariance, our model is designed to be invariant to rigid transformations (rotations) by constructing graphs based on Euclidean distances and cell‐type labels. Experimental validation on the V10S14‐085_XY04‐21–0057 dataset [36] demonstrates high clustering consistency under orthogonal rotations (Figures S11 and S12), outperforming other methods.

### 2.4 SOTMGF Enables Cell Trajectory Analysis to Infer Cellular Evolution and Differentiation Processes

To delineate cellular differentiation trajectories and infer the evolution and differentiation processes between cells, we applied SOTMGF to the HybISS spatial dataset of the developing brain [37]. By integrating reference scRNA‐seq data, SOTMGF successfully inferred high‐resolution spatial differentiation trajectories and reconstructed dynamic developmental changes at single‐cell resolution.

**FIGURE 4:**
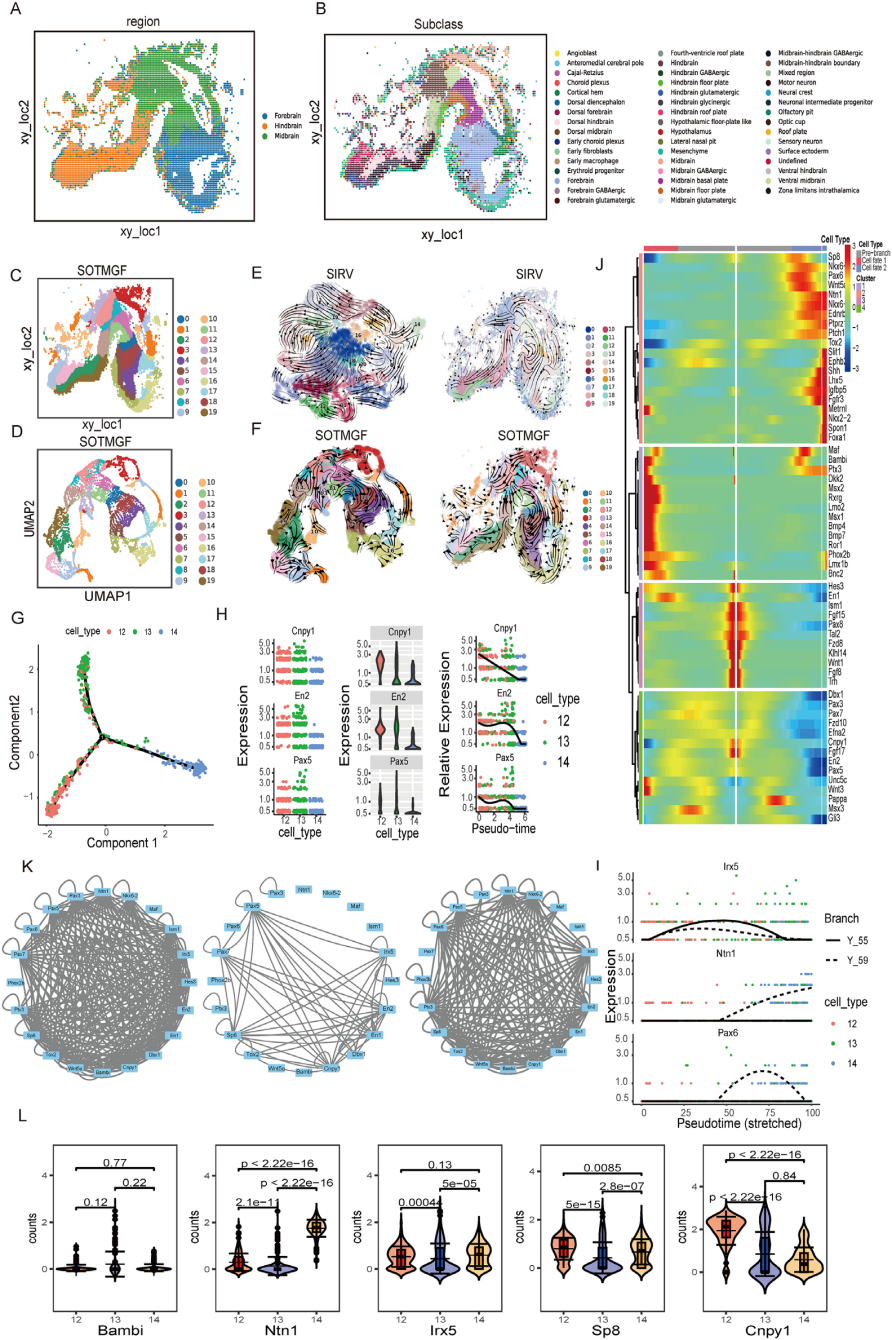
SOTMGF enables cell trajectory analysis on developing mouse brain data measured using the HybISS protocol. (A) Spatial map showing the location of each ‘Region’ label in the tissue annotated by SIRV. (B) Spatial map showing the location of each ‘Subclass’ label annotated by SIRV in the tissue. (C) Cluster assignments generated by SOTMGF from the HybISS spatial dataset of the developing mouse brain. (D) Scatter plot of the two‐dimensional UMAP extracted from latent features by SOTMGF. (E) Main flow of RNA velocities visualized by velocity streamlines, projected on a UMAP plot and spatial domain plot of the HybISS data based on SIRV. (F) Main flow of RNA velocities visualized by velocity streamlines, projected on a UMAP plot and spatial domain plot of the HybISS data based on SOTMGF. (G) Visualization of clustering and trajectory inference from SOTMGF clusters 12, 13, and 14. (H) Pseudotime‐dependent changes in the expression levels of *Cnpy1*, *En2*, and *Pax5*. Each color indicates one cluster. (I) Distribution of gene expression by BEAM analysis. (J) Heatmap of gene expression analyzed by BEAM analysis. (K) Average conditional cell‐specific networks of clusters 12, 13, and 14 with the 20 selected branching‐related genes. The edge between two genes indicates direct dependency. (L) Violin plot of gene expression of Bambi, which inhibits brain development, and the genes *Ntn1*, *Sp8*, *Irx5*, and *Cnpy1*, which promote brain development, in clusters 12, 13, and 14.

Annotations of different brain regions, including the forebrain, midbrain, and hindbrain, along with their finer subclass labels, were accurately transferred from single‐cell RNA sequencing (scRNA‐seq) to spatial data using SIRV [38] (Figure 4A,B). The HybISS dataset was clustered using SOTMGF, yielding spatially organized clusters in both low‐dimensional UMAP visualizations and the original spatial coordinates (Figure 4C,D).

RNA velocity analysis based on SOTMGF‐reconstructed expression profiles revealed continuous developmental flows that closely aligned with spatial domains and delineated branching trajectories across clusters (Figure 4E,F). The inferred trajectories were more continuous and spatially consistent than those from SIRV, enabling reconstruction of lineage relationships obscured in non‐spatial or low‐resolution data. Notably, clusters 12 and 13 at the midbrain–hindbrain boundary exhibited a three‐way divergence toward clusters 3, 8, and 10. Similarly, cluster 8 in the forebrain differentiated indirectly toward cluster 11 via cluster 6. Additional trajectories included cluster 0 branching into clusters 6, 11, and 18; convergence of clusters 4 and 18 into cluster 0; and an extended hindbrain path from cluster 2 merging with cluster 9 into cluster 15 (Figure 4F). By projecting velocity vectors into a spatial context, SOTMGF revealed intricate differentiation dynamics, including indirect and multi‐step trajectories, which are not discernible from scRNA‐seq data alone.

Pseudotime trajectory analysis using Monocle2 was performed on key clusters (12, 13, 14, and 0, 11, 18, 6) to reconstruct branches patterns and developmental progression (Figure 4G,H; Figures S13 and S14). This analysis identified several genes critical to the development of the midbrain‐hindbrain boundary (MHB), including Cnpy1, which modulates FGF signaling at the MHB, as well as En2, Pax2, and Pax5, known for their contributions in regional patterning and neurodevelopment [39, 40, 41].

To identify branching drivers, we utilized Branched Expression Analysis Modeling (BEAM). With the SOTMGF denoised SPE profiles (Figure 4I,J; Figures S15 and S16). The results indicate that Pax6 is recognized as a master regulator of neurogenesis, serving as a key branching gene with high expression in cluster 14. Figure 4J This highlights SOTMGF's capability to reveal subtle yet functionally critical gene expression patterns that are overlooked by raw data analysis.

To quantitatively assess branching dynamics, we plotted the average gene associations of 20 branching‐related genes across clusters 12, 13, and 14 (Figure 4K). Clusters 12 and 14 (located at or near the MHB) exhibited stronger gene associations, whereas cluster 13 (hindbrain region) showed markedly weaker associations, consistent with its less dynamic differentiation state. This result was supported by violin plot analysis showing high expression of developmental inhibitors (Bambi [42], Hes3 [43]) and low expression of promotive factors (Ntn1 [44], Sp8 [45], Irx5 [46, 47], Cnpy1 [48]) in cluster 13 (Figure 4L; Figure S17).

In conclusion, these results demonstrate the capability of SOTMGF to reconstruct high‐resolution, spatially coherent differentiation trajectories and uncover novel regulatory dynamics by effectively integrating spatial and single‐cell transcriptomic data.

### 2.5 SOTMGF Enhances the Delineation of Tumor Boundaries and Enables the Identification of Ligand‐Receptor Signaling Pathways in Invasive Carcinoma

**FIGURE 5:**
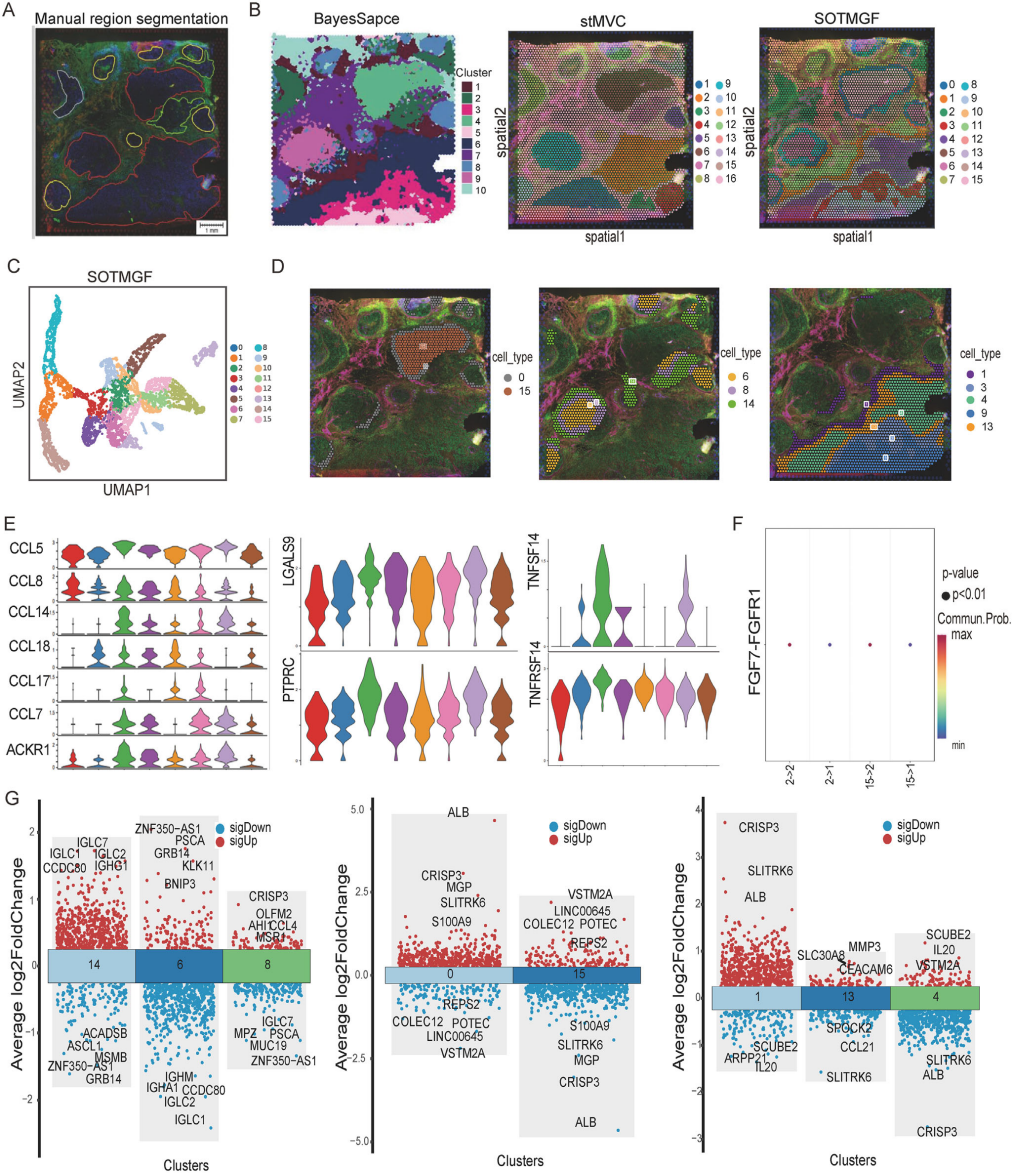
SOTMGF enhances the delineation of tumor boundaries. (A) Immunofluorescent staining of IDC tissue shows 12 regions: invasive carcinoma (red), carcinoma in situ (orange), and benign hyperplasia (green). The intensities of DAPI, fiducial frame, and anti‐CD3 are indicated by green, blue, and yellow, respectively. (B) Spatial clustering comparison among BayesSpace, stMVC, and SOTMGF. (C) UMAP visualization of latent features by SOTMGF, with each color indicating a different cluster. (D) Spatial distribution maps of structural domains 6, 8, and 14; structural domains 0 and 15; and structural domains 1, 4, and 13. (E) Violin plots showing the expression levels of key genes encoding receptors and ligands in IDC. (F) Dot plot illustrating FGF signaling pathway conductance between clusters 0, 1, and 14, highlighting important ligand‐receptor pairs. Dot color reflects communication probabilities, and dot size represents computed p‐values. Empty spaces indicate zero communication probability. P‐values are computed from a one‐sided permutation test. (G) Volcano plot showing differential gene expression per structural domain, with up‐regulated genes indicated in red and down‐regulated genes in blue.

We applied SOTMGF to analyze the IDC dataset, which includes 16 regions (Figure 5A). Compared to methods such as BayesSpace [49] and stMVC [50], SOTMGF is more effective in detecting structural domains within cancer regions—particularly in the invasive carcinoma area (marked in red), where it identified five distinct clusters (including clusters 3, 4, and 9), outperforming other methods that detected at most three or four (Figure 5B). The feature embeddings extracted by SOTMGF also demonstrated improved separation between different cellular states, providing insights into the cancer microenvironment and progression.

**FIGURE 6:**
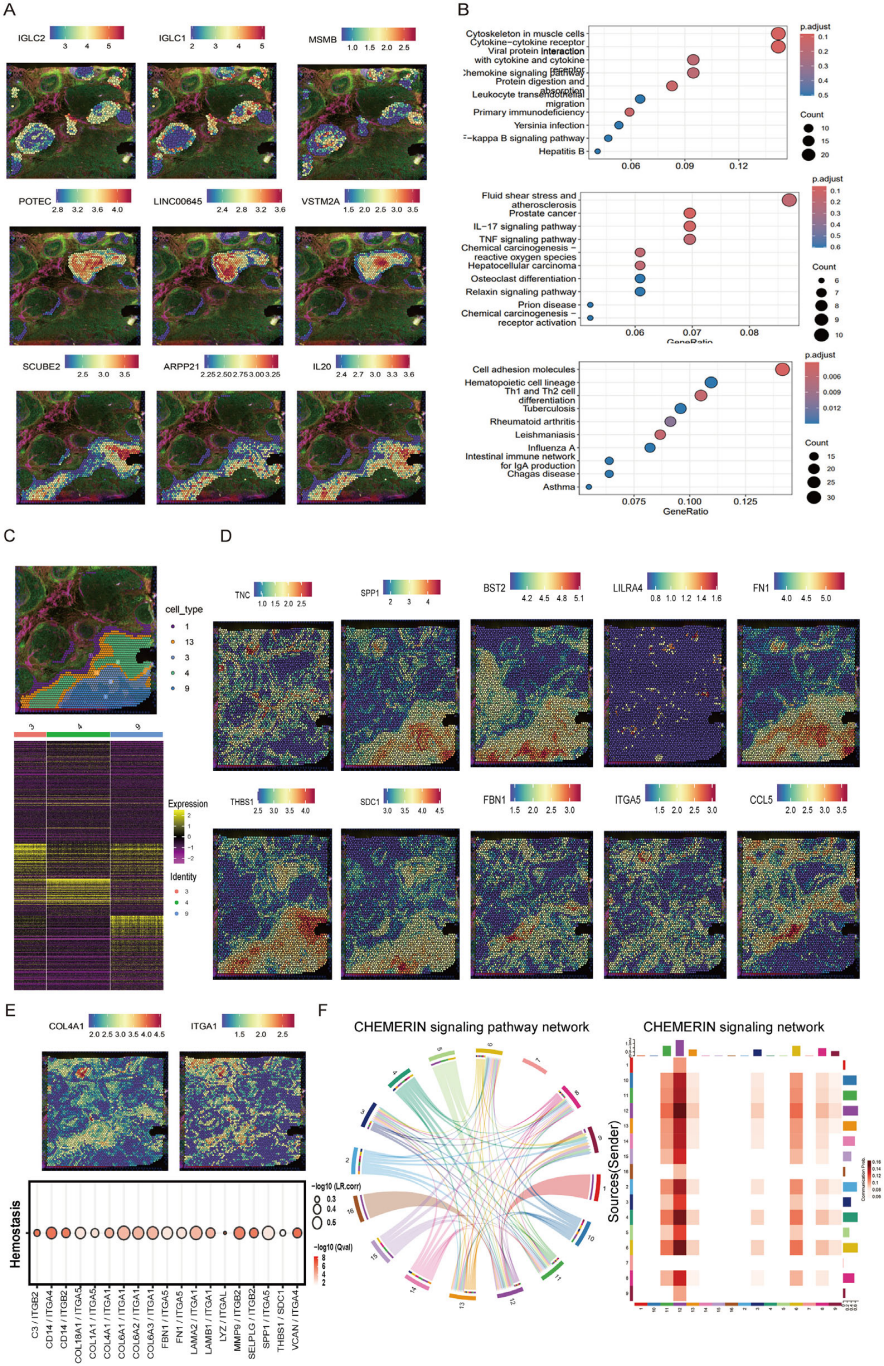
SOTMGF enables identification of ligand‐receptor signaling pathways in invasive carcinoma. (A) Spatial expression of genes for indicative markers: plasma B cells (IGLC7, IGLC2), MSMB, oncogenes (LINC00645), proliferation‐promoting gene (POTEC), immunosuppression gene (SCUBE2), and tumor progression genes (ARPP21, IL20) in these domains. (B) Gene function enrichment analysis of SVGs in domains 0, 1, and 14 was conducted using the R package ClusterProfiler. The enrichment test was performed using KEGG. (C) Spatial distribution map of structural domains 1, 3, 4, 9, and 13. The heatmap shows the gene expression of signature genes from three domains enriched in the invasive carcinoma region as identified by SOTMGF. Rows represent genes, and columns represent domains. (D) Representative spatial distributions of LRI in structural domains 3, 4, and 9. (E) Spatial distribution and bubble map of the gene COL4A1 and its receptor, illustrating their response to tumor progression. (F) The inferred GALECTIN signaling network shows the relative contribution of each ligand‐receptor pair to the overall GALECTIN signaling network in IDC.

SOTMGF initially defined tumor boundaries using spatial clustering: clusters 0, 1, and 14 were localized to the edge of the invasive carcinoma region (red area), whereas clusters 4, 6, 8, 13, and 15 were entirely within the invasive zone (Figure 5C,D). This boundary delineation outperformed methods such as BayesSpace [49] and stMVC [50], because SOTMGF extracted feature embeddings with superior cell state separation, and in the lower‐left invasive region, it identified 5 clusters (including distinct clusters 3, 4, 9) compared to a maximum of 3–4 clusters identified by other tools (Figure 5B). Signal transduction and differential gene analyses further validated the structural and functional specificity of the tumor boundary regions identified by SOTMGF. The combined edge clusters (0, 1, and 14) exhibited ten distinct signaling pathways, which is one more than the inner invasive clusters, including a unique FGF pathway mediated by FGF7‐FGFR1 (Figure 5E,F; Figure S18). Differential gene expression highlighted pronounced microenvironmental heterogeneity(Figure 5G): domain 14 (adjacent to inner tumor regions) showed upregulation of plasma B cell markers (IGLC2, IGLC7); domain 0 was enriched in immune‐related (e.g., ALB) and angiogenesis genes; while domain 1—surrounding inner clusters 4 and 13—displayed elevated expression of fibroblast‐associated and immunosuppressive genes such as SCUBE2 [51], along with proliferation and invasion promoters ARPP21 and IL20 (Figure 6A). KEGG enrichment analysis confirmed the functional specialization of these boundary domains: cluster 14 was associated with cellular immunomodulation [52], cluster 0 with angiogenesis and cell proliferation [53, 54], and cluster 1 with cell adhesion and antigen uptake (Figure 6B).

Furthermore, SOTMGF identified 70 biologically relevant ligand‐receptor (LR) interactions across the tissue (Figure S19), highlighting key signaling activities within distinct invasive clusters. In cluster 3, SPP1–ITGA5 and FN1–ITGA5 were enriched and found to activate extracellular matrix organization, promoting cell adhesion and invasion [55, 56, 57]. Cluster 4 exhibited the interaction between THBS1–SDC1, which is also involved in matrix remodeling. In contrast, cluster 9 showed high expression of BST2, which is associated with breast cancer progression [58] (Figure 6C,D). Spatial expression analysis revealed that COL4A1 is predominantly localized to domains 10 and 11 adjacent to the invasive region, consistent with its known functions in malignant mesenchymal tissues [59] (Figure 6E). Further analysis of the signaling pathway revealed ten pathways across all domains, with the CHEMERIN pathway, which was primarily mediated by the LGALS9–CD45 pair, being particularly relevant to immune escape. This interaction inhibits immune surveillance and reduces metastasis [60], with CD45 serving as a key immune regulator [61]. Circle plot visualization highlighted signaling crosstalk between domains 3, 4, 6, and 15 with domains 5, 10, and 11(Figure 6F). Importantly, CHEMERIN signaling was uniquely detected in domains 3, 6, and 13, all located within the invasive carcinoma region, underscoring their pro‐tumorigenic functional role.

In summary, SOTMGF provides a powerful framework for delineating tumor microstructure and characterizing molecular heterogeneity, identifying key ligand‐receptor pairs, and uncovering signaling pathways within these regions, uncovering critical targets for cancer progression and immune evasion in breast cancer treatment.

### 2.6 SOTMGF Enables Integrative Analysis of Spatial Multi‐Omics with MERFISH and SHARE‐seq

**FIGURE 7:**
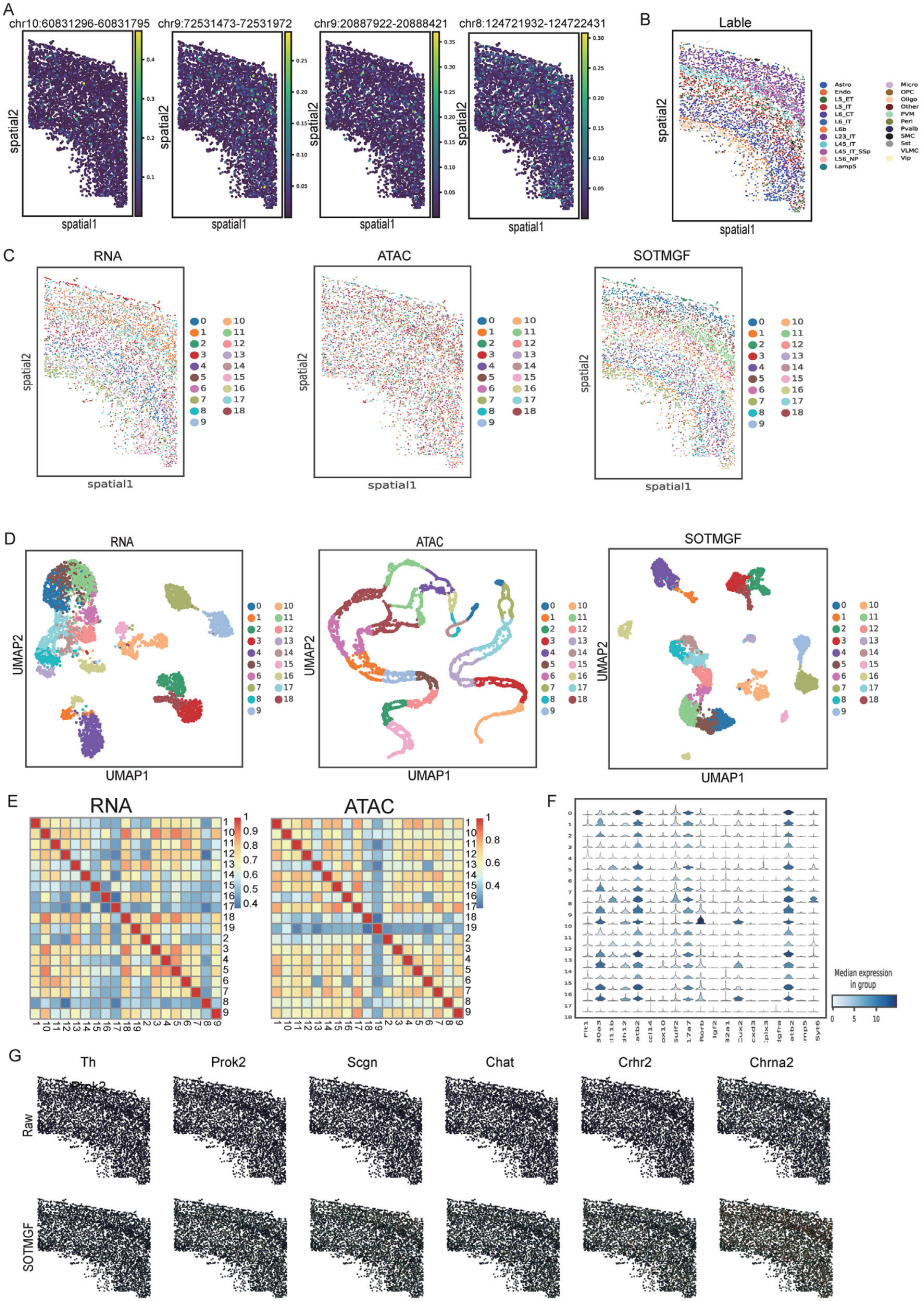
SOTMGF can perform joint analysis with MERFISH and SHARE‐seq. (A) Patterns of predicted chromatin accessibility. Spatial patterns of chromatin accessibility predicted by Tangram. (B) Predicted deterministic cell type composition. The composition of deterministic cell types predicted by Tangram. (C) Visualization of spatial domains detection using ST, chromatin accessibility with spatial patterns, and the joint analysis of chromatin accessibility with spatial patterns and ST. (D) Scatter plot of the two‐dimensional UMAP extracted from latent features with ST, chromatin accessibility with spatial patterns, and the joint analysis of chromatin accessibility with spatial patterns and ST. (E) Cluster‐to‐cluster Spearman correlations between chromatin accessibility with spatial patterns and ST. (F) Expression levels of differentially expressed genes across clusters in raw spatial transcriptome data. (G) Spatial expression patterns of SDGs in a MOP region, denoised using SOTMGF, with raw data provided for comparison.

To overcome the limitation of current spatial sequencing technologies, which predominantly capture single‐omics spatial information (e.g., ST), we developed an integrative strategy leveraging single‐cell multi‐omics profiles to infer spatially resolved multi‐omics information. Specifically, we integrated two datasets: 1) MERFISH data providing spatially resolved RNA expression in mouse brain [62], and 2) SHARE‐seq data simultaneously profiling RNA expression (over 16 000 genes) and chromatin accessibility (ATAC‐seq, over 400 000 peaks) in >3000 cells [63]. Our approach involved two key steps: First, we aligned spatial RNA patterns between MERFISH and the RNA component of SHARE‐seq using Tangram [64]. Then, leveraging the successfully mapped RNA spatial location as a scaffold, we inferred the spatial patterns of chromatin accessibility profiled by SHARE‐seq to reconstruct a spatially resolved multi‐omics profile (Figure 7A). We also obtained the probability matrix of finding each cell in the sc/snRNA‐seq data in each voxel of the spatial data. Based on this matrix, we can obtain a deterministic mapping as a reference by assigning the most probable sc/snRNA‐seq cell to each spatial voxel (Figure 7B).

We obtained low‐dimensional embeddings of transcriptome and chromatin accessibility data via SOTMGF using ST data and chromatin accessibility data with spatial patterns. After this step, we connected the transcriptome and chromatin accessibility features and used them as inputs to an interpretable Transformer model to learn the joint features to obtain the final clustering results. These results show that combined spatial multi‐omics data analysis achieves better performance in identifying spatial domains and visualizing clustering results (Figure 7C). Meanwhile, we generated scatter plots of the two‐dimensional UMAP extracted from latent features. Compared with models that consider ST and chromatin accessibility data separately, the model that combines ST and chromatin accessibility with spatial patterns clearly distinguished each cluster (Figure 7D). Upon visualizing the weights of the different omics in the final clustering, it was evident that in clusters 8, 13, and 18, the transcriptomes were weighted more, whereas in clusters 1, 6, and 10, chromatin accessibility contributed more to the final clustering results (Figure S20). In addition, we calculated Spearman correlations for gene expression and chromatin accessibility between clusters (Figure 7E). Chromatin accessibility data correlations were calculated based on the average peak counts per cluster, while transcriptome correlations were calculated based on the average gene expression within each cluster.

We also calculated the spatially highly variable genes (SVGs) in each cluster and visualized the distribution of these SVGs (Figure 7F). Finally, we performed differential gene analysis. We obtained SVGs by performing differential gene analysis on the raw data, and then denoising the raw data by calculating the average of the 15 spots neighboring each spot in the potential embedding space. For the denoised data, we can identify genes that exhibit insignificant differences in the raw expression data but become significant in the denoised data through differential gene analysis (Figure 7G). For example, exogenous up‐regulation of Prok2 inhibits ferroptosis and improves motor and cognitive ability in traumatic brain injury models [65], and intervention targeting the Prok2‐Fbxo10‐Acsl4 signaling pathway significantly improves motor and cognitive prognosis in mice after craniocerebral trauma. These effects became significant only after denoising (Figure 7G). This demonstrates that SOTMGF can uncover biologically relevant spatial gene patterns with increased sensitivity.

## 3 Conclusions

Spatial multi‐omics data have independently revolutionized our understanding of complex biological processes by enabling in situ profiling of molecular landscapes within intact tissues [11]. The advent of spatial omics technologies has enabled researchers to gain spatial insights into transcriptomics, proteomics, metabolomics, and even multi‐omics [1]. However, the simultaneous integration of spatial multi‐omics data on a single tissue section in a spatially informative manner remains an unmet need and a substantial challenge [15]. Existing approaches often treat each omics modality in isolation or rely on cross‐tissue alignments, which fail to capture the precise spatial correlations between molecular layers that are essential for understanding tissue function and disease mechanisms.

The continuous advancement of spatial multi‐omics sequencing technologies presents significant challenges for the intelligent analysis of spatial multi‐omics data, particularly in identifying spatial domains. Spatial domains refer to multicellular neighboring structures formed by cells in space, closely associated with specific biological functions [66]. Profound analysis of spatial domains is crucial for understanding developmental processes, discovering novel disease biomarkers, elucidating tumor heterogeneity, and characterizing the tumor microenvironment. Current methods, however, struggle to integrate the diverse information from multi‐omics modalities while preserving spatial fidelity, leading to incomplete or inaccurate domain characterization.

To address these challenges, we developed SOTMGF, a self‐supervised, goal‐directed multi‐view graph fusion framework for spatial omics data analysis. SOTMGF integrates multi‐modality information from spatial multi‐omics using dual self‐supervised learning to extract multi‐view cellular embedding representations, which are then integrated through an attention mechanism. We evaluated the effectiveness of attention‐based multi‐view fusion by using the average of all view representations as a comparison. In addition, an ablation study was conducted to assess the contribution of each view. The importance of the individual view was further assessed by testing with only two views and three views.

Notably, SOTMGF distinguishes itself from existing methods through four key aspects: (a) SOTMGF leverages SP and ST data from the same tissue section to integrate multidimensional information from spatial multi‐omics and extract latent low‐dimensional embeddings for joint analysis, which captures post‐transcriptional regulatory dynamics that are critical for understanding functional phenotypes. (b) Existing spatial sequencing technologies mainly capture single‐omics spatial information but lack information on the corresponding additional omics. In order to expand the scope of applicable datasets for SOTMGF, we computationally generated spatial ATAC‐seq data corresponding to ST using Tangram, which allows for the spatial multi‐omics integration analysis of RNA expression and ATAC‐seq data, uncovering epigenetic regulatory mechanisms that drive spatial gene expression patterns. (c) SOTMGF simultaneously incorporates multi‐modality information from spatial omics, including molecular expression, physical location, neighboring cell type distribution, and molecular associations, and fuses these multi‐modality data via an attention mechanism. This ensures that no critical information, including microenvironmental or molecular association data, is overlooked. (d) SOTMGF consists of five modules, in which the self‐training process and graph embedding iteratively cycle within a unified framework, learning and optimizing together so that the components mutually benefit to further enhance clustering performance. Evaluations on data from different platforms and spatial resolutions further confirm that the low‐dimensional embeddings generated by SOTMGF accurately capture the hierarchical structure of spatial omics data.

SOTMGF offers advantages such as modularity, reproducibility, and flexibility. In practical data analysis, researchers can selectively construct relevant views for cell embedding representations, making it widely applicable to the integration of spatial multi‐omics. Applied to a mouse breast cancer dataset with co‐registered ST and SP, SOTMGF improved spatial domain detection, identified novel SDPs and SDGs, and predicted key TFs. Biomarkers such as Notch4, CD117, and Rbpj—implicated in tumor progression and metastasis—were highlighted, offering potential value for diagnostics and targeted therapy [67]. Applied to the human DLPFC dataset, SOTMGF demonstrated excellent performance in identifying spatial domains. SOTMGF revealed cell differentiation trajectories from a spatial perspective via RNA velocity analysis, uncovering changes in molecular associations during cell development and differentiation intensity across different spatial domains. Applied to a human breast cancer IDC dataset, SOTMGF successfully identified tumor boundaries, dissected the cancer microenvironment, and identified ligands and receptors related to disease progression. Finally, by integrating MERFISH‐based ST with SHARE‐seq‐derived chromatin accessibility data, we demonstrated how SOTMGF supports spatially informed multi‐omics integration for biomarker and target discovery.

Despite these strengths, our work also has limitations: (a) SOTMGF requires pre‐specification of the number of domains, which may be challenging in the absence of prior knowledge, which is a common issue for most unsupervised clustering methods. (b) The biological and clinical implications of SDPs and SDGs identified by SOTMGF require further validation through experimental and clinical studies.

In conclusion, SOTMGF provides a flexible and powerful framework for integrating spatial multi‐omics data to decipher spatial domains and elucidate cell heterogeneity. Its ability to capture spatial‐omics synergy, identify dark molecules, and resolve functional cellular trajectories positions it as a valuable tool for advancing both basic biological discovery and clinical translation.

## 4 Experimental Section/Methods

### 4.1 Preclustering Using Deep Attentional Embedded Graph Clustering

We perform preclustering using DAEGC [68], which encodes the topology and node content graphs into a compact representation. This is achieved by employing an attentional network to capture the importance of neighboring nodes relative to the target node. An inner‐product decoder is then trained on this representation to reconstruct the graph structure. Additionally, soft labels derived from the graph embeddings themselves are generated to supervise the self‐training graph clustering process, which iteratively refines the clustering results. This self‐training process is jointly optimized with the embedded graph in a unified framework.

The graph used for preclustering is represented as *G* = (*V*,  *X^pre^*, *A^pre^*), where *V* = {*v_i_*}_*i* = 1, 2, ···*n*_ consists of a set of nodes. *X^pre^* is the cell type composition of each observation unit, and *A^pre^* is the adjacency matrix ($A^{pre}\in R^{n\times n}$). The adjacency matrix *A^pre^* of the shared nearest neighbor graph is constructed by first defining a radius *r*, then setting $A_{ij}^{pre}=1$ if the Euclidean distance between *v_i_* and *v_j_* is less than *r*, and $A_{ij}^{pre}=0$ otherwise.

For the representation of the graph structure *A^pre^* and node content *X^pre^* in a unified framework, SOTMGF developed a variant of the graph attention network as a graph encoder. To measure the importance of different neighbors, the neighbor representations are assigned different weights according to the following equation:

(1) $$z_{i}^{l+1}=\sigma \left(\sum_{j\in N_{i}}\left(\alpha_{ij}Wz_{j}^{l}\right)\right)$$

where $z_{i}^{l+1}$ indicates the output representation of node *i*, σ is a nonlinear function, and *N_i_* represents the neighbors of *i*. α_*ij*_ is the attention coefficient indicating the importance of neighboring node *j* to node *i*. This coefficient measures the importance of neighboring node *j* in terms of both attribute value and topological distance.

In terms of attribute values, the attention coefficients α_*ij*_ can be expressed as:

(2) $$\alpha_{ij}={\vec{\alpha }}^{T}\left[Wx_{i}∥Wx_{j}\right]$$

where $\vec{\alpha }\in R^{2m^{\prime }}$.

In terms of topology, neighboring nodes contribute to the representation of the target node through edges. The proximity matrix is obtained by considering t‐order neighboring nodes in the graph, and is defined as:

(3) $$M=\left(B+B^{2}+\cdots +B^{t}\right)/t$$

where *B* is the transfer matrix, with *B_ij_* = 1/*d_i_* if and only if *e_ij_* ∈ *E*, and *B_ij_* = 0 otherwise. Here, *d_i_* represents the degree of node *i*. The element *M_ij_* indicates the topological correlation of node *j* with node *i* up to the t‐order. There is considerable flexibility in choosing *t* for different datasets, allowing a balance between the accuracy and efficiency of the model.

The attention coefficients are normalized using the softmax function over all neighborhoods $j\in N_{i}$, where *N_i_* represents the neighboring nodes of *i* in *M*. Together with the topological weights *M* and the activation function δ, the attention coefficients can be expressed as:

(4) $$\alpha_{ij}=\frac{\exp\left(\delta M_{ir}\left({\vec{\alpha }}^{T}\left[W_{x_{i}}∥W_{x_{j}}\right]\right)\right)}{\sum_{r\in N_{i}}\exp\left(\delta M_{ir}\left({\vec{\alpha }}^{T}\left[W_{x_{i}}∥W_{x_{j}}\right]\right)\right)}$$

where δ represents LeakyReLU.

Given the input $x_{i}=z_{i}^{0}$, two graph attention layers are defined as:

(5) $$z_{i}^{\left(1\right)}=\sigma \left(\sum_{j\in N_{i}}\alpha_{ij}W^{\left(0\right)}x_{j}\right)$$

(6) $$z_{i}^{\left(2\right)}=\sigma \left(\sum_{j\in N_{i}}\alpha_{ij}W^{\left(1\right)}z_{i}^{\left(1\right)}\right)$$

These layers encode the structure and node attributes as hidden representations such that $z_{i}=z_{i}^{\left(2\right)}$. The reconstructed adjacency matrix is obtained using the inner product decoder:

(7) $${\hat{A}}_{ij}=sigmoid\left(z_{i}^{T}z_{j}\right)$$

The reconstruction loss is defined as:

(8) $$L_{recon}=\sum_{i=1}^{n}loss\left(A_{ij},\;{\hat{A}}_{ij}\right)$$

In addition to optimizing the reconstruction error, we incorporate hidden embedding inputs into the self‐optimizing clustering module, in which the following objectives are minimized:

(9) $$L_{cluster}=KL\left(P∥Q\right)=\sum_{i}\sum_{u}p_{iu}log\frac{p_{iu}}{q_{iu}}$$

The similarity between the node embedding *z_i_* and the cluster center embedding µ_*u*_ is measured with Student's t‐distribution, which can be regarded as a soft clustering assignment distribution for each node defined as,

(10) $$q_{iu}=\frac{{\left(1+∥z_{i}-\mu_{u}{∥}^{2}\right)}^{-1}}{\sum_{k}{\left(1+∥z_{i}-\mu_{k}{∥}^{2}\right)}^{-1}}$$

And *p_iu_* is the target distribution defined as:

(11) $$p_{iu}=\frac{q_{iu}^{2}/\sum_{i}q_{iu}}{\sum_{k}\left(q_{ik}^{2}/\sum_{i}q_{ik}\right)}$$

DAEGC jointly optimizes the autoencoder embedding extraction and clustering learning process with a total objective function defined as:

(12) $$L=L_{recon}+\gamma L_{cluster}$$

where γ ≥ 0 is a coefficient balancing them. The estimated labels can be obtained as follows:

(13) $$s_{i}=argmax_{u}q_{iu}$$

By leveraging the attentional network and the iterative refinement process, DAEGC enhances the clustering quality by effectively integrating both topological and content information, thereby improving the preclustering phase and laying a robust foundation for subsequent analyses.

### 4.2 Extracting Molecular Expression Potential Embedding via Transformer

The Transformer is a sequence modeling neural network architecture based on a self‐attention mechanism. This mechanism considers all positions of the input sequence for computation, effectively capturing global correlations within the input sequence. The Transformer follows an encoder‐decoder architecture and employs both a scaled dot‐product attention mechanism and a multi‐head attention mechanism for its encoder and decoder components.

The encoder comprises a stack of N = 3 identical layers. Each layer consists of two sublayers: a multi‐head self‐attention mechanism and a fully connected feed‐forward network. We utilize residual connections and layer normalization around each of these sublayers. Specifically, the output of each sublayer is given by LayerNorm(*x* + Sublayer(*x*)), where Sublayer(*x*) represents the function implemented by the sublayer itself. To facilitate these residual connections, all sublayers in the model, as well as the embedding layers, produce outputs of dimension d_*model*_ = 1024.

The decoder also consists of a stack of N = 3 identical layers. In addition to the two sublayers present in each encoder layer, the decoder inserts a third sublayer that performs multi‐head attention on the output of the encoder stack. Residual connections are again used around each sublayer, followed by layer normalization.

Multi‐Head Attention is a core component of the transformer model, employing self‐attention across various representation subspaces. This approach enables the model to focus on information in multiple subspaces while maintaining computational efficiency. The input to Multi‐Head Attention comprises three vectors: the query vector (Q), key vector (K), and value vector (V). These vectors, representing raw molecular expression matrices, are linearly transformed into *h* groups (heads) of different feature vectors.

(14) $$\mathrm{MultiHead}\left(Q,K,V\right)=\mathrm{Concat}\left(head_{1},\text{…},\;head_{h}\right)W^{O}$$

where $head_{i}=\mathrm{Attention}\left(QW_{i}^{Q},KW_{i}^{K},VW_{i}^{V}\right)$, the projections are parameter matrices $W_{i}^{Q}\in R^{d_{model}\times d_{k}}$, $W_{i}^{K}\in R^{d_{model}\times d_{k}},$ $W_{i}^{V}\in R^{d_{model}\times d_{v}}$ and $W^{O}\in R^{hd_{v}\times d_{model}}$. Additionally, $\mathrm{Attention}\left(Q,K,V\right)=\mathrm{softmax}\left(\frac{QK^{T}}{\sqrt{d_{k}}}\right)V$.

First, Q and K undergo an inner product to obtain the cosine similarity between them, which is then divided by $\sqrt{d_{k}}$ to scale the attentional weights. Subsequently, the softmax function is applied to derive the weights. This scaling of the attention weights, based on the dot‐product attention mechanism, is termed Scaled Dot‐Product Attention.

In this work, we employ *h* parallel attention heads. For each head, we set $d_{k}=d_{v}=\frac{d_{model}}{h}$. Due to the reduced dimensionality of each head, the total computational cost remains comparable to that of single‐head attention with full dimensionality.

After extracting the dense representation of the original molecular expression signature, the similarity between the node embedding *z_i_* and the cluster center embedding µ_*u*_ is measured using Student's t‐distribution. This can be regarded as a soft clustering assignment distribution for each node, defined as:

(15) $$q_{iu}=\frac{{\left(1+∥z_{i}-\mu_{u}{∥}^{2}\right)}^{-1}}{\sum_{k}{\left(1+∥z_{i}-\mu_{k}{∥}^{2}\right)}^{-1}}$$

The target distribution *p_iu_* is defined as:

(16) $$p_{iu}=\frac{q_{iu}^{2}/\sum_{i}q_{iu}}{\sum_{k}\left(q_{ik}^{2}/\sum_{i}q_{ik}\right)}$$

The loss function of the Transformer module is:

(17) $$L_{trans}=L_{deco}+\lambda \cdot L_{auto}+KL\left(P∥Q\right)$$

where *L_auto_* is the cross‐entropy between the adjacency matrices *A* and *A*′, λ = 8, and *E*′ is the decoder of the Transformer module, defined as the inner product between the low‐dimensional feature *R* and *R^T^*: *E*′ = *sigmoid*(*R* ·  *R^T^*).

*L_deco_* is defined as:

(18) $$\begin{array}{ccc} L_{deco} & = & -\frac{1}{N\times N}\sum_{i=1}^{N}\sum_{j=1}^{N}\left(e_{ij}\times \log\left(e_{ij}\right)\right.\\ & & +\;\left.\left(1-e_{ij}\right)\times \log\left(1-e_{ij}\right)\right) \end{array}$$

We process the lower‐dimensional features to obtain the spot class prediction. The prediction *Y*′ = *softmax*(*R*), and *L_auto_* is defined as:

(19) $$L_{auto}=\frac{1}{u}\sum_{l=1}^{u}\left(-\sum_{i=1}^{K}y_{i}\log\left(y_{i}^{\prime }\right)\right)$$

where *u* is the number of spots that include training sets, *K* is the number of classes of labels, *y_i_* is the true class, and $y_{i}^{\prime }$ is the predicted class.

The Transformer module also incorporates a self‐optimizing clustering module, in which the following objective is minimized:

(20) $$KL\left(P∥Q\right)=\sum_{i}\sum_{u}p_{iu}log\frac{p_{iu}}{q_{iu}}$$

where $q_{iu}=\frac{{\left(1+∥z_{i}-\mu_{u}{∥}^{2}\right)}^{-1}}{\sum_{k}{\left(1+∥z_{i}-\mu_{k}{∥}^{2}\right)}^{-1}}$, $p_{iu}=\frac{q_{iu}^{2}/\sum_{i}q_{iu}}{\sum_{k}\left(q_{ik}^{2}/\sum_{i}q_{ik}\right)}$.

### 4.3 Constructing Each View by Exploiting Different Information

#### 4.3.1 Processing Spatial Location Information to Construct the Spatial Neighbor Network

The location information provided by spatial omics data bridges gene expression information and tissue image information.

The node feature *X* is a potential embedding of the original gene expression extracted by a transformer, and *A* is an adjacency matrix ($A\in R^{n\times n}$). For the spatial neighbor network, given a radius *r*, the adjacency matrix $A_{ij}^{snn}$ is defined such that: $A_{ij}^{snn}=1$ if the Euclidean distance between *v_i_* and *v_j_* is less than *r*. Otherwise, $A_{ij}^{snn}=0$.

#### 4.3.2 Combining Precluster Labels to Construct Cell Type‐Aware Spatial Network

For spatial omics data with lower resolution, the SOTMGF introduces a cell type‐aware module to prune the spatial neighbor network based on the results of DAEGC‐based preclustering (or MDAGC clustering). Specifically, pre‐clustering clusters the cell‐type distribution obtained by deconvolution, combined with physical location via the DAEGC algorithm. MDAGC clustering extracts low‐dimensional latent features across multi‐views and performs fusion clustering.

If two spots belong to different clusters, the edge is pruned, as illustrated in Figure 1. This pruning process better characterizes the heterogeneous spatial similarity between neighboring spots. For the cell type‐aware spatial network, given a radius *r*, the adjacency matrix $A_{ij}^{csn}$ is defined such that: $A_{ij}^{csn}=1$ in case the Euclidean distance between *v_i_* and *v_j_* is less than the radius *r*, *v_i_* and *v_j_* belong to same cluster. Otherwise, $A_{ij}^{csn}=0$.

#### 4.3.3 Building Microenvironment Network Based on the Distribution of Node Cell Types

To capture cellular microenvironmental information, SOTMGF constructs the microenvironment network. In this graph, nodes represent dense features of molecular expression extracted by a transformer, and edges are defined by connectivity‐guided, molecular‐expression‐aware cell‐neighborhood minimum graph distance. Initially, the frequency of each cell cluster (determined by DAEGC preclustering results or MDAGC clustering results) within the cellular space neighborhood is calculated to obtain a histogram.

The connectivity‐guided minimum graph distance (CGMGD) is then computed to approximate distances in the spatial omics state space by integrating the UMAP local data structure with the PAGA global topology. The CGMGD is computed in the following steps:

Evaluate Connectivity: Using PAGA, the connectivity between cell clusters is assessed to generate a binary connectivity matrix (BCM), indicating whether an edge exists between two cell clusters.

Calculate Pairwise Distances: The pairwise distances of cluster centers in the UMAP embedding space of individual cells are calculated to generate a UMAP distance matrix (UDM).

Each node in the microenvironment network represents a cell cluster. The edges of the graph are defined by the adjacency matrix, which is obtained by multiplying the elements of the BCM and UDM.

Compute CGMGD: The CGMGD is determined by searching the microenvironment network for the pairwise minimum distance.

The variable *f_pq_* is determined by the following optimization function:

$$\min\sum_{p=1}^{t}\sum_{q=1}^{t}f_{pq}CGMGD\left(p,q\right)$$

$$s.t.\;\left\{\begin{array}{c} f_{pq}\geq 0,p,q\in \left[1,t\right]\\ \sum_{p=1}^{t}f_{pq}\leq w_{q}^{MECN_{j}},p,q\in \left[1,t\right],j\in \left[1,n\right]\\ \sum_{q=1}^{t}f_{pq}\leq w_{p}^{MECN_{i}},p,q\in \left[1,t\right],i\in \left[1,n\right] \end{array}\right.,$$

where *n* and *t* represent the number of cells and cell clusters, $\mathrm{MEC}N_{i}=\left\{\left(T_{p},w_{T_{p}}^{i}\right)\right\},\;T_{p}\in \left[1,t\right],\;i\in \left[1,n\right].\;T_{p}$ enumerates all possible cell cluster labels and $w_{T_{p}}^{MECN_{i}}$ is the total normalized count of *T_p_* clusters in *MECN_i_*, the *CGMGD*(*p*, *q*) is precomputed between all pairs of cell clusters. *f_pq_* represents the relative (normalized) quantity transferred from cell cluster *p* of microenvironment *i* to cell cluster *q* of microenvironment *j*. The distance between *MECN_i_* and *MECN_j_* is defined as:

(21) $$\mathrm{dist}\left(\mathrm{MEC}N_{i},\mathrm{MEC}N_{j}\right)=\frac{\sum_{p=1}^{t}\sum_{q=1}^{t}f_{pq}CGMGD\left(p,q\right)}{\sum_{p=1}^{t}\sum_{q=1}^{t}f_{pq}}$$

So, the edge of the microenvironment network is defined as:

(22) $$A_{ij}^{micro}=\frac{\sum_{p=1}^{t}\sum_{=1}^{t}f_{pq}CGMGD\left(p,q\right)}{\sum_{p=1}^{t}\sum_{q=1}^{t}f_{pq}}$$

#### 4.3.4 Constructing Molecular Association Network by Exploiting Molecular Association Matrix

The use of molecular association data has been found to significantly improve the performance of single‐cell clustering [72]. We employ the cellular association matrix of the SRT data to construct a direct association network between genes for each cell. The conditional independence of genes *x* and y given the conditional gene *z* in cell *k* is determined using the statistical index:

(23) $$\rho_{xy|z}^{\left(k\right)}=\frac{n_{xyz}^{\left(k\right)}}{n_{z}^{\left(k\right)}}-\frac{n_{xz}^{\left(k\right)}}{n_{z}^{\left(k\right)}}\cdot \frac{n_{yz}^{\left(k\right)}}{n_{z}^{\left(k\right)}}$$

which ranges from −1 to 1. Here, $n_{z}^{\left(k\right)},\;n_{xz}^{\left(k\right)}\;,\;n_{yz}^{\left(k\right)},\;\mathrm{and}\;n_{xyz}^{\left(k\right)}$ are the respective numbers of cells in the neighborhoods of *z_k_*, (*x*_*k*,_ *z_k_*), (*y*_*k*,_ *z_k_*), and (*x*_*k*,_ *y*_*k*,_ *z_k_*). Genes *x* and *y* in cell *k* are associated if $\rho_{xy}^{\left(k\right)}$ is greater than the significance level, indicating an edge exists between them. Conversely, *x* and *y* in cell *k* are independent, with no edges between them.

To reduce computational costs, we identify direct associations between pairs of genes in a cell with a small number of conditional genes, which may be key regulatory genes in biological processes, such as TFs and kinases. From a network perspective, the conditional genes are often hub genes in the gene‐gene network. The default parameters used in constructing the network are *alpha* = 0.5,  *kk* = 1,  *boxsize* = 1.5,  and *weighted* = 1. These can be adjusted according to specific requirements.

The Conditional Cell‐specific Network (CCSN) obtained for cell *k* under conditional gene *z* is denoted as $c_{ij}^{\left(k\right)}$. We can reduce the dimensionality while integrating the network properties. The conditional network degree of the *i*‐th gene in the *k*‐th cell is given by $v_{ik}=\sum_{j=1}^{m}c_{ij}^{\left(k\right)}$.

This allows us to obtain a conditional degree matrix containing *m* × *n* elements. Next, we extract low‐dimensional latent embeddings from the conditional degree matrix. To achieve this, we process the conditional degree matrix $V=\{v_{ik}\left|i=1,\;2,\;\text{…},\;num\left(gene\right),\;j=1,\;2,\;\text{…},\;num\left(cell\right)\right\}$ with an autoencoder.

We construct the molecular association graph by processing the gene expression features *w_i_* to form edge features, using the conditional gene expression matrix features as node features. This is defined as:

(24) $$A_{ij}^{\mathrm{GAG}}=\frac{w_{i}\cdot w_{j}}{\sqrt{{\left|w_{i}\right|}^{2}+{\left|w_{j}\right|}^{2}}}$$

### 4.4 Learning Low‐Dimensional Representations of Each View by Deep Attention‐Guided Graph Clustering with Dual Self‐Supervision(DAGC)

First, a DAE (Denoising Autoencoder) module with a series of encoders and decoders is utilized to extract the low‐dimensional representation $X_{trans}^{1}$ by optimizing the reconstruction loss:

(25) $$L_{R}=∥X-\hat{X}{∥}_{F}^{2}$$

where ***X*** and $\hat{X}$ represent the input matrix and the reconstruction matrix, respectively. Specifically, $X=X_{trans}^{1}$, $\hat{X}={\hat{X}}_{trans}^{1}$, The encoder and decoder operations at layer *i* are defined as: $H_{i}=\phi \left(W_{i}^{e}H_{i-1}+b_{i}^{e}\right)$, ${\hat{H}}_{i}=\phi \left(W_{i}^{d}{\hat{H}}_{i-1}+b_{i}^{d}\right)$, where ***H**_i_* and ${\hat{H}}_{i}$ denote the outputs of the encoder and decoder at layer *i*, respectively. Here, *l* denotes the number of layers in the encoder and decoder, while $W_{i}^{e}$, $b_{i}^{e}$, $W_{i}^{d}$, $b_{i}^{d}$ indicate the weights and biases of the encoder‐decoder at layer *i*. The function ϕ(·) signifies the activation function.

The GCN (Graph Convolutional Network) features learned from layer *i* are denoted as $Z_{i}\in R^{n\times d_{i}}$, where *d_i_* indicates the dimensionality of layer *i*, and ***Z***_0_ = ***X***. The ***Z**_i_* and ***H**_i_* are then adaptively and dynamically weighted by the Heterogeneity‐Wise Fusion (HWF) [72]. The corresponding attention coefficient weights are defined as:

(26) $$M_{i}\in \left[m_{i,1}∥m_{i,2}\right]=\gamma_{A}\left(\left[Z_{i}∥H_{i}\right]W_{i}^{a}\right)$$

where $M_{i}\in R^{n\times 2}$ is the matrix of attention coefficients, and ***m***_*i*,1_ and ***m***_*i*,2_ are the weight vectors measuring the importance of ***Z**_i_* and ***H**_i_*, respectively. The function γ_*A*_(·) is defined as: $\gamma_{A}\left(\cdot \right)=\ell_{2}\left(softmax(LReLU(\cdot ))\right)$.

The adaptively fused ***Z**_i_* and ***H**_i_* are given by $Z_{i}^{\prime }=\left(m_{i,1}1_{i}\right)⊙Z_{i}+\left(m_{i,2}1_{i}\right)⊙H_{i}$,

where 1_*i*_ indicates a matrix with all elements equal to 1, and ⊙ denotes the Hadamard product of matrices. Then, $\;Z_{i}^{\prime }\in R^{n\times d_{i}}$ is utilized as the input to layer *i* + 1 to learn ***Z***_*i* + 1_:

(27) $$Z_{i+1}=LReLU\left(D^{-\frac{1}{2}}\left(A+I\right)D^{-\frac{1}{2}}Z_{i}^{\prime }W_{i}\right)$$

where ***W**_i_* denotes the weight of the *i*‐th GCN layer and $D^{-\frac{1}{2}}\left(A+I\right)D^{-\frac{1}{2}}$ performs normalization on ***A***.

In conjunction with this, SOTMGF introduces a Scale‐Wise Fusion (SWF) module that connects different layers of multiscale features through an attention‐based mechanism. A fully connected layer $W^{s}\in R^{\left(\sum_{j=1}^{l}d_{j}+d_{l}\right)\times \left(l+1\right)}$ parameterized by a weight matrix, is designed to capture the relationships among multiscale features. The process is denoted as:

(28) $$U=\gamma_{A}\left(\Xi_{j=1}^{l+1}Z_{j}W_{i}^{s}\right)$$

where $\Xi_{j=1}^{l+1}Z_{j}=\left[Z_{1}∥\text{…}∥Z_{l}∥Z_{l+1}\right]$ represents the concatenation operation of multiple elements. Subsequently, feature fusion is performed as follows:

(29) $$Z^{\prime }=\Xi_{j=1}^{l+1}\left(\left(\mu_{j}1_{j}\right)⊙Z_{i}\right)$$

here, ***µ**_j_* denotes the *j*‐th element of **U**, and $\Xi_{j=1}^{l+1}u_{j}=U$. This ultimately yields a fusion feature ***Z*** defined by:

(30) $$Z=softmax\left(D^{-\frac{1}{2}}\left(A+I\right)D^{-\frac{1}{2}}Z^{\prime }W\right)$$

where ***W*** denotes the learning parameter.

The center embedding ***µ*** of K‐means is computed using the gene expression feature ***H*** obtained from DAE. The similarity between the extracted feature ***h**_i_* and its corresponding centroid vector ***µ**_j_* is then calculated. This similarity can be regarded as a probability distribution ***Q***, whose *i*, *j*‐th element is $q_{i,j}=\frac{{\left(1+∥h_{i}-\mu_{j}{∥}^{2}\right)}^{-1}}{\sum_{j^{\prime }}{\left(1+∥h_{i}-\mu_{j^{\prime }}{∥}^{2}\right)}^{-1}}$.

The DWF module predicts final clustering labels in an attention‐driven manner by fusing learned probability distributions. The importance of ***Z*** and ***Q*** is determined using the attention mechanism:

(31) $$V=\left[v_{1}∥v_{2}\right]=\gamma_{A}\left(\left[Z∥Q\right]\hat{W}\right)$$

where $V\in R^{n\times 2}$ is the attention coefficient matrix, and $\hat{W}$ is the weight matrix learned through the fully connected layer. The module adaptively leverages ***Z*** and ***Q*** as follows:

(32) $$F=\left(v_{1}1\right)⊙Z+\left(v_{2}1\right)⊙Q$$

here, 1_*i*_ represents a vector with all elements equal to 1. The resulting ***F*** is then normalized using the softmax function:

(33) $$F=softmax\left(F\right)\;s.t.\sum_{j=1}^{k}f_{i,j}=1,\;f_{i,j}>0$$

where *f*_*i*,*j*_ is an element in ***F***. After the network is adequately trained, the predicted label can be inferred as: $y_{i}=\arg max_{j}\;f_{i,j}\;s.t.\;j=1,\text{…},k$.

Finally, a dual self‐supervision strategy is employed to guide the entire network training, combining a soft self‐supervision strategy based on KL divergence and a hard self‐supervision strategy leveraging pseudo‐supervised loss.

#### 4.4.1 Soft Self‐Supervision

Initially, by squaring $z_{i,j}^{t}$, an auxiliary distribution is derived:

(34)

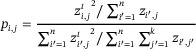

where 0 ≤ *p*_*i*,*j*_ ≤ 1 is an element of ***P***. We then minimize *KL*(***P***, ***Q***), *KL*(***P***, ***Z***), and *KL*(***Z***, ***Q***) to promote a highly consistent distributional alignment model. The loss function for soft self‐supervision is given by:

(35) $$L_{S}=\lambda_{1}*\left(KL\left(P,Z\right)+KL\left(P,Q\right)\right)+\lambda_{2}*KL\left(Z,Q\right)$$

where λ_1_ > 0 and λ_2_ > 0 are trade‐off parameters.

#### 4.4.2 Hard Self‐Supervision

To further utilize the information obtained from cluster assignments, we introduce a pseudo‐supervision technique with pseudo‐labels ${\hat{y}}_{i}=y_{i}$. Considering that pseudo‐labels may contain many incorrect labels, we select supervised information with high confidence by setting a very high threshold value:

(36) $$g_{i,j}=\left\{\begin{array}{c} 1\;\;\;if\;f_{i,j}>r\;\\ 0\;\;\;otherwise \end{array}\right.$$

where *r* = 0.7. Training is then supervised with high‐confidence pseudo‐labeling. The loss function for hard self‐supervision is:

(37) $$L_{H}=\lambda_{3}\sum_{i}\sum_{j}g_{i,j}*\gamma_{CE}\left(f_{i,j},\;\gamma_{OH}\left({\hat{y}}_{i}\right)\right)$$

where λ_3>0 is the weighted parameter, γ_*CE*_ denotes the cross‐entropy loss, and γ_*OH*_ denotes one‐hot encoding for ${\hat{y}}_{i}$.

#### 4.4.3 Combined Loss Function

With the combination of the soft self‐supervised strategy (SSS) and the hard self‐supervised strategy (HSS) to drive network training, the overall loss function can be expressed as:

(38) $$L=\mathrm{mi}n_{F}\left(L_{R}+L_{S}+L_{H}\right)$$

By integrating these strategies, the network achieves robust and consistent training outcomes [72].

### 4.5 Learning Final Low‐Dimensional Representations by Attention Mechanism

The above process is performed for each view to obtain the low‐dimensional embeddings $F_{SLG}^{t}$, $F_{SLG-C}^{t}$, $F_{MEG}^{t}$ and $F_{GAG}^{t}$. Let $F_{1}^{t}=F_{SLG}^{t}$, $F_{2}^{t}=F_{SLG-C}^{t}$, $F_{3}^{t}=F_{MEG}^{t}$, and $F_{4}^{t}=F_{GAG}^{t}$ for each view, where *t* denotes the *t*‐th iteration.

The weights are learned adaptively using the attention mechanism. The features from different views are connected through an attention‐based mechanism. Denote the whole process as:

(39) $$U_{M}=\gamma_{A}\left(\Xi_{m=1}^{4}F_{m}^{t}W_{m}^{M}\right)$$

where $\Xi_{m=1}^{4}F_{m}^{t}=\left[F_{1}^{t}∥F_{2}^{t}∥F_{3}^{t}∥F_{4}^{t}\right]$ denotes the concatenation operation of multiple elements. Then, feature fusion is performed:

(40) $$Z^{\prime }=\Xi_{m=1}^{4}\left(\left(\mu_{m}1_{j}\right)⊙F_{m}^{t}\right)$$

where ***µ**_m_* denotes the *m*‐th element of **U**_***M***_. Since $\Xi_{j=1}^{4}u_{j}=U_{M}$, the final fused feature is obtained as:

(41) $$Z^{t}=softmax\left(D^{-\frac{1}{2}}\left(A+I\right)D^{-\frac{1}{2}}Z^{\prime }W\right)$$

where ***W*** denotes the learning parameter, *t* denotes the *t*‐th iteration, and $A\in R^{n\times n}$ denotes the adjacency matrix.

Finally, the dual self‐supervision strategy combines the soft self‐supervision strategy (SSS) and the hard self‐supervision strategy (HSS) to guide the overall network training. The overall loss function can be expressed as:

(42) $$L=\mathrm{mi}n_{F}\left(L_{R}+L_{S}+L_{H}\right)$$

### 4.6 Datasets Description and Preprocessing

#### 4.6.1 General Processing

Unless otherwise stated, data preprocessing was performed using Scanpy (version 1.8.2) with default parameters. Raw counts were normalized per cell by total counts using sc.pp.normalize_total, followed by log1p transformation (sc.pp.log1p). Highly variable genes were selected using the Seurat method, retaining the top 2000 genes (sc.pp.highly_variable_genes, flavor = ‘seurat’, n_top_genes = 2000). For all datasets, the cellular association matrix of SRT data was used to construct a cell‐specific gene–gene network for each cell [72]. Cell type compositions were obtained via deconvolution using the CARD1.1 R package with default parameters, except for the MOP dataset, where deconvolution was performed with Tangram.

#### 4.6.2 Simulation Dataset

Simulated spatial multi‐omics datasets were generated by SpatialGlue using non‐negative spatial factorization [15]. SpatialGlue employed the “ggblocks” model to build expression matrices across modalities: Modality 1 was a spatial transcriptomic matrix (1296 cells × 1000 genes) following a zero‐inflated negative binomial (ZINB) distribution, while Modality 2 simulated spatial proteomic data (1296 cells × 100 proteins) under a negative binomial distribution. To reduce stochasticity, five simulated dual‐modality datasets with varying parameters were generated. A tri‐modality dataset (transcriptome, proteome, and epigenome) was also created. Gaussian noise was added to all modalities to mimic biological variability. Datasets are available at https://drive.google.com/drive/folders/1PsYs62vbA_VLxvMZOV82GZ5Dr7btNY3O.

For the simulated dataset, all clustering analyses and evaluation metrics were performed in Python. Scanpy was used for data preprocessing, dimensionality reduction, and clustering (Leiden algorithm). Visualization of clustering outcomes and evaluation metrics was carried out using Matplotlib.

#### 4.6.3 Mouse Breast Cancer and Mouse Spleen

This dataset contains both protein and transcript measurements [11], generated using SPOTS with 10x Visium technology, which captures whole transcriptomes and extracellular proteins via polyadenylated antibody‐derived tag‐conjugated antibodies. Data were downloaded fromGene Expression Omnibus (GEO) under accession GSE198353. Corresponding scRNA‐seq data were obtained from GEO under accession GSE158677.

Gene expression visualization for this dataset was performed in R (using ggplot2, pheatmap, etc.). Clustering results, UMAP plots, and modality weight analysis were implemented in Python. Prognostic analysis was conducted using GEPIA2, and pathway diagrams were created with Figdraw.

#### 4.6.4 Human Dorsolateral Prefrontal Cortex (DLPFC) 10X Visium Datasets

The dataset consists of 12 tissue slices from three human brains. The six neuronal layers and the white matter (WM) layer were manually annotated as the ground truth for benchmarking. Datasets were downloaded from http://spatial.libd.org/spatialLIBD/. Corresponding scRNA‐seq data were obtained from GEO accession GSE144136.

Gene expression visualization was again performed in R. Clustering results, UMAP plots, and evaluation metrics were visualized using Python (Scanpy and Matplotlib).

#### 4.6.5 V10S14‐085_XY04‐21–0057

The kidney sample V10S14‐085_XY04‐21–0057 from chronic kidney disease (CKD) patients, sequenced on the 10× Visium platform, was downloaded from the KPMP atlas (https://atlas.kpmp.org/repository/ ). Cell types in each spot were annotated as epithelial, endothelial, immune, or stromal by experienced nephrology physicians from KPMP. Corresponding scRNA‐seq data were downloaded from https://zenodo.org/records/6410326.

#### 4.6.6 Developing Mouse Brain

Spatial transcriptomics and scRNA‐seq datasets were obtained from the Developing Mouse Brain Atlas (http://mousebrain.org/downloads.html ). Tamim Abdelaal et al. used a voxelized version of the data, summarizing spatial gene expression on a 2D grid of 30 000 pixels [38]. Annotations of major brain regions (forebrain, midbrain, hindbrain) and finer subclass labels were transferred from scRNA‐seq to spatial data using SIRV. Datasets were downloaded from https://doi.org/10.5281/zenodo.6798659. Corresponding scRNA‐seq data were obtained from http://mousebrain.org/downloads.html.

Clustering results, UMAP plots, and evaluation metrics were generated in Python. RNA velocity calculation and visualization were performed using SIRV and scVelo. Pseudotime analysis was carried out in R with Monocle2, and gene association networks were visualized using Cytoscape.

#### 4.6.7 IDC

The raw count matrix, histology, and spatial location data for IDC samples are publicly available on the 10X Genomics website (https://support.10xgenomics.com/spatialgene‐expression/datasets). Corresponding scRNA‐seq data were downloaded from https://singlecell.broadinstitute.org/single_cell/study/SCP1039.

Clustering results, UMAP plots, and evaluation metrics were visualized in Python. Differential expression analysis and cell–cell communication analysis were performed in R (e.g., DESeq2, FindMarkers, CellChat).

#### 4.6.8 Mouse Primary Motor Cortex (MOP)

MERFISH data provided spatially resolved RNA expression in the mouse brain [62], and SHARE‐seq data simultaneously profiled RNA expression (>16 000 genes) and chromatin accessibility (>400 000 peaks) in >3000 cells [63]. MERFISH MOP data are available at the Brain Image Library (https://doi.brainimagelibrary.org/doi/10.35077/g.21) [72]. SHARE‐seq dataset is available https://www.ncbi.nlm.nih.gov/geo/query/acc.cgi?acc=GSE140203. First, we aligned spatial RNA patterns between MERFISH and the RNA component of SHARE‐seq using Tangram [64]. Then, using the successfully mapped RNA spatial locations as a scaffold, we inferred the spatial patterns of chromatin accessibility from SHARE‐seq to reconstruct a spatially resolved multi‐omics profile. Tangram also provided a probability matrix indicating the likelihood of each sc/snRNA‐seq cell being located in each spatial voxel. Based on this matrix, we assigned the most probable sc/snRNA‐seq cell to each spatial voxel to obtain a deterministic mapping reference.

All figures for this dataset (including clustering, dimensionality reduction, and evaluation metrics) were generated in Python using Scanpy, Matplotlib, and Seaborn.

## Author Contributions

Y.J.L. conceived and designed the study, implemented the model, performed all the experiments, and wrote the manuscript with feedback from all authors. R.Q. analyzed the IDC dataset and wrote the corresponding manuscript. Y.J.L., R.Q., Y.L., J.G., P.L.L., and L.N. Chen revised the manuscript. L.N.C. and P.L.L. supervised the work and critically reviewed the paper. The authors read and approved the final manuscript.

## Conflicts of Interest

The authors declare no conflicts of interest.

## Supporting information




**Supporting File**: advs75533‐sup‐0001‐SuppMat.docx.

## Data Availability

All data analyzed in this paper are available in publicly available datasets. Specifically, murine breast spatial profiling data consisting of protein and transcript measurements are available from GEO under accession GSE198353. High‐resolution images of the tissues used in the present study are available at the Figshare website (https://figshare.com/account/home#/projects/143019), the corresponding scRNA‐seq data were downloaded from the GEO, accession no. GSE158677, the gene annotation file of Mus musculus from Ensembl to get the ID transformation files of genes and proteins is publicly available at (https://ftp.ensembl.org/pub/release‐106/gtf/mus_musculus/). The 12 slices of human DLPFC dataset11 are available from spatialLIBD (http://spatial.libd.org/spatialLIBD/) [73], the corresponding scRNA‐seq data were downloaded from GEO, accession no. GSE144136. The kidney sample from CKD patients sequenced on 10× Visium platform are downloaded from the KPMP atlas https://atlas.kpmp.org/repository/. The developing mouse brain data is available at (https://doi.org/10.5281/zenodo.6798659). The simulated datasets are available at (https://drive.google.com/drive/folders/1PsYs62vbA_VLxvMZOV82GZ5Dr7btNY3O). The raw count matrix, histology, and spatial location data for IDC samples are publicly available at the 10X Genomics Website (https://support.10xgenomics.com/spatialgene‐expression/datasets), the corresponding scRNA‐seq data were downloaded from (https://singlecell.broadinstitute.org/single_cell/study/SCP1039). The MERFISH MOP data are available at the Brain Image Library (https://doi.brainimagelibrary.org/doi/10.35077/g.21) [72], and the corresponding SHARE‐seq dataset are available at (https://www.ncbi.nlm.nih.gov/geo/query/acc.cgi?acc=GSE140203). The SOTMGF algorithm is implemented in Python and is available on Github [https://github.com/Luyj6812/SOTMGF].
