# Supplementary material for: Combining Spatial Multi‐Omics Data to Decipher Spatial Domains and Elucidate Cell Heterogeneity Based on Self‐Supervised Graph Learning
Source: Adv Sci (Weinh). 2026 May 11;13(42):e75533. doi: 10.1002/advs.75533 (PMC13335623; doi:10.1002/advs.75533)
Supplement: Supplementary file 1 — Supporting File: advs75533‐sup‐0001‐SuppMat.docx. [file ADVS-13-e75533-s001.docx]

Supporting Information

**Combining spatial multi-omics data to decipher spatial domains and elucidate cell** **heterogeneity based on self-supervised graph learning**

*Yuejing Lu^1,3†^, Rui Qiao^1†^, Ying Li^4^,* *Junhong Li^9^, Nuoya Yue^1^, Jing Ge^5^, Jiao Wang^9,10*^,* *Luonan Chen^6,7,8^, Peiluan Li^1,2*^*

^1^ School of Mathematics and Statistics, Henan University of Science and Technology, Luoyang 471023, China.

^2^ Guangdong Provincial Key Laboratory of Mathematical and Neural Dynamical Systems, Great Bay University, Dongguan, 52300, China.

^3^ School of Artificial Intelligence, Wuhan University, Wuhan 430072, China.

^4^ College of Electronic and Information Engineering, Shenzhen University, Shenzhen 518060, Guangdong, China.

^5^ Shanghai Immune Therapy Institute, Renji Hospital, Shanghai Jiao Tong University School of Medicine, Shanghai 200032, China.

^6^School of Mathematical Sciences and School of AI, Shanghai Jiao Tong University, Shanghai 200240, China，

^7^Tianfu Jincheng Laboratory, Chengdu, 610212, China，

^8^Key Laboratory of Systems Health Science of Zhejiang Province, Hangzhou Institute for Advanced Study, University of Chinese Academy of Sciences, Hangzhou 310024, China.

^9^ School of Life Sciences, Shanghai University, Shanghai 200444, China.

^10^ School of Life Science and Technology, Shandong Vocational University of Foreign Affairs Jinan, 250131. China,

^†^These authors contributed equally to this work

**Supporting Information text**

**Comparison with other spatial domain detection methods.**

We use the default parameter settings for all methods and the same number of clusters in clustering.

**SpatialGlue:**

We used SpatialGlue algorithm for clustering. For all datasets, a learning rate of 0.0001 was used. To account for differences in feature distribution across the datasets, a tailored group of weight factors [γ1, γ2, γ3, γ4] was empirically assigned to each one. We also provided a default parameter set that would work for most users on most data types. The training epochs used for the simulated datasets were 200.  In their experiment, SpatialGlue finds ‘mclust’ algorithm performs better than ‘leiden’ and ‘louvain’ on spatial data in most cases. Therefore, SpatialGlue recommend using ‘mclust’ algorithm for clustering[1].

**MISO:**

MISO is a deep-learning based method developed for the integration and clustering of multi-modal spatial omics data. MISO requires minimal hyperparameter tuning, and can be applied to any number of omic and imaging data modalities from any multi-modal spatial omics experiment[2]. MISO has been evaluated on datasets from experiements including spatial transcriptomics (transcriptomics and histology), spatial epigenome-transcriptome co-profiling (chromatin accessibility, histone modification, and transcriptomics), spatial CITE-seq (transcriptomics, proteomics, and histology), and spatial transcriptomics and metabolomics (transcriptomics, metabolomics, and histology). We use the default parameter settings for MISO and the same number of clusters in clustering.

**MultiVI:**

By default, MultiVI is optimized using AdamW with a learning rate of 0.0001, weight decay of 0.001 and minibatch size of 128. As in previous models, we trained on 90% the data and used 10% as a validation set[3]. We selected an initial training plan consisting of 500 epochs but the model is stopped early if there is no improvement in the reconstruction loss on the validation dataset for 50 epochs (early stopping). We down-weighted the KL divergence between the latent representation and its prior during the first 50 epochs (for $i\in\left[ 1,50 \right], KL\cdot\frac{i}{50}$). In addition, a domain adaptation penalty is included in the training schemes to increase mixing in the latent space. Briefly, a classifier is created using a two-layer feed forward neural network with 32 hidden units. Its output is the probability for each cell to belong to one of the batch keys. We use the output of this classifier to create a cross-entropy loss that is adversarially trained.

**SpaceFlow:**

We used SpaceFlow algorithm for clustering [4]. The preprocessing of the raw count expression matrix of spatial transcriptomics (ST) data involves several steps. Initially, genes that are expressed in fewer than three cells, as well as cells expressing fewer than 100 genes, are excluded.. The resulting log-transformed expression matrix, containing the top 3000 highly variable genes (HVGs), is utilized as input for constructing the spatial expression graph. To select highly expressed genes, we employ a dispersion-based method. Genes are categorized into 20 bins. For constructing the Spatial Expression Graph (SEG), the default setting for the number of nearest neighbors $k$ for each cell or spot is 15; increasing $k$ expands the spatial neighborhood. The DeepGraphInfomax (DGI) model, implemented via the PyTorch Geometric library, is employed. The default size for low-dimensional embeddings is set to 50. The encoder for SEG is a two-layer Graph Convolutional Network (GCN), using Parametric ReLUs (PReLU) as activation functions. The number of neurons in both layers matches the low-dimensional embedding size.

**DeepST:**

We applied DeepST[5]to DLPFC dataset to detect spatial domain. Initially, DeepST excluded regions outside the primary tissue area from all datasets. The Scanpy package [6] was employed to filter, log-transform, and standardize the raw gene expression data relative to library size. DeepST utilizes Principal Component Analysis (PCA) to reduce dimension of gene expression data. The reduced dimensionality data then serve as input for subsequent model training. Based on DeepST embeddings, we employed the Leiden algorithm (via Scanpy[6]) to identify spatial domains. DeepST determines the optimal resolution through two approaches: (1) When the number of spatial domains is known, it performs a grid search between 0.1 and 2.5, with an increment of 0.01, until the desired number of clusters is achieved. (2) In the absence of prior knowledge, DeepST uses a grid search across resolutions from 0.1 to 2.5, with a step size of 0.01, concurrently calculating the Calinski and Harabasz (CH) score using sklearn ultimately selecting the resolution with the highest CH score.

**conST:**

We applied conST[7] to DLPFC dataset to detect spatial domain. conST set $K=20$ for constructing the KNN graph. Principal Component Analysis (PCA) is applied to reduce the gene expression dimensions to $F_{1}=300$. The morphological features are reduced to a dimension of $F_{2}=768$ Users have the flexibility to adjust these parameters to fit the needs of specific datasets. Both stages undergo 200 training epochs individually. For downstream analysis, the Leiden algorithm is employed for clustering.

**stCluster:**

As default settings, we set radius=150, ae_rate=0.8, adj_rate=0.2, pred_rate=0.3, seed=0. we used their default parameter settings to learn the latent representations. We then applied their default clustering methods to generate spatial domains for comparison[8].

**GraphST**

Follow the default settings, we set reconstruction loss weight and contrastive loss weight λ1 and λ2 as 10 and 1. The training of this module is independent of the next scRNA-seq and ST data integration module, and we employ the Adam optimizer for the optimization. The learning rate and training epoch are set to 0.001 and 600 for both spatial clustering and multiple ST data integration tasks, while 0.001 and 1200 are used for the scRNA-seq and ST data integration task[9].

**Details of datasets**

**Simulated data**

The simulated datasets are available at (<https://drive.google.com/drive/folders/1PsYs62vbA_VLxvMZOV82GZ5Dr7btNY3O>).

**Murine breast cancer data consisting of protein and transcript measurements**

We demonstrate SOTMGF ’s capabilities with murine breast cancer spatial profiling data consisting of protein and transcript measurements. The data was generated using SPOTS with the 10x Visium technology capturing whole trancriptomes and extracellular proteins with polyadenylated antibody-derived tag-conjugated (ADT-conjugated) antibodies. Dataset link: ([GEO Accession viewer (nih.gov)](https://www.ncbi.nlm.nih.gov/geo/query/acc.cgi?acc=GSE198353))

High-resolution images of the tissues used in the present study are available at the Figshare website (<https://figshare.com/account/home#/projects/143019>), the corresponding scRNA-seq data were downloaded from the Gene Expression Omnibus (GEO), accession no. GSE158677, the gene annotation file of Mus musculus from Ensembl to get the ID transformation files of genes and proteins is publicly available at ([Index of /pub/release-106/gtf/mus_musculus (ensembl.org)](https://ftp.ensembl.org/pub/release-106/gtf/mus_musculus/)).

**Murine brain data consisting of protein and transcript measurements**

MERFISH technology enables true in situ single-cell spatial genomics analysis to visualize gene expression and localization in a dataset containing 4188 cells and 254 genes. MERFISH MOp data are available at the Brain Image Library (<https://doi.brainimagelibrary.org/doi/10.35077/g.21>).

SHARE-seq technology enables simultaneous high-quality, high-throughput detection of gene expression and chromatin accessibility in single cells. SHARE-seq dataset are available (<https://www.ncbi.nlm.nih.gov/geo/query/acc.cgi?acc=GSE140203>).

**Human tonsil dataset**

10x Visium human tonsil gene and protein expression data (https://www.10xgenomics.com/resources/ datasets/gene-protein-expression-library-of-human-tonsil-cytassistffpe-2-standard).

**Some Analysis of Spatial Transcriptomics**

**Metrics**

**Adjusted Rand Index (ARI)**

The ARI is the corrected-for-chance version of the Rand index (RI), which is a measure of the similarity between two clusters. ARI can be obtained by:

$$ARI=\frac{\max\left( RI \right)-E(RI)}{RI-E(RI)},$$

where $RI$ is:

$$RI=\frac{TP+TN}{TP+FP+TN+FN}$$

**Average Silhouette Width (ASW)**

The Silhouette value is used to evaluate the similarity of a destination to other clusters in the cluster where the destination is located[10]. The range of Silhouette value is from -1 to +1. The value reflects the matching relationship between the target and their own clusters, a high value indicates a high matching relationship with other clusters, conversely, a low value indicates a low matching relationship with other clusters. If the value is high, then the clustering result is better, if it is small or negative, then it may be caused by too many or too few clusters.

Suppose the dataset has been divided into many classes by the clustering algorithm and for the objective $i$ there are $i\in C_{i}$,obtaining:

$$a\left( i \right)=\frac{1}{|C_{i}-1|}\sum_{j\in C_{i},i\neq j} d(i,j)$$

where $a\left( i \right)$ represents the average distance between$i$ and other targets between the same cluster. $d(i,j)$ is the distance of targets $i$ and $j$ in cluster $C_{i}$.


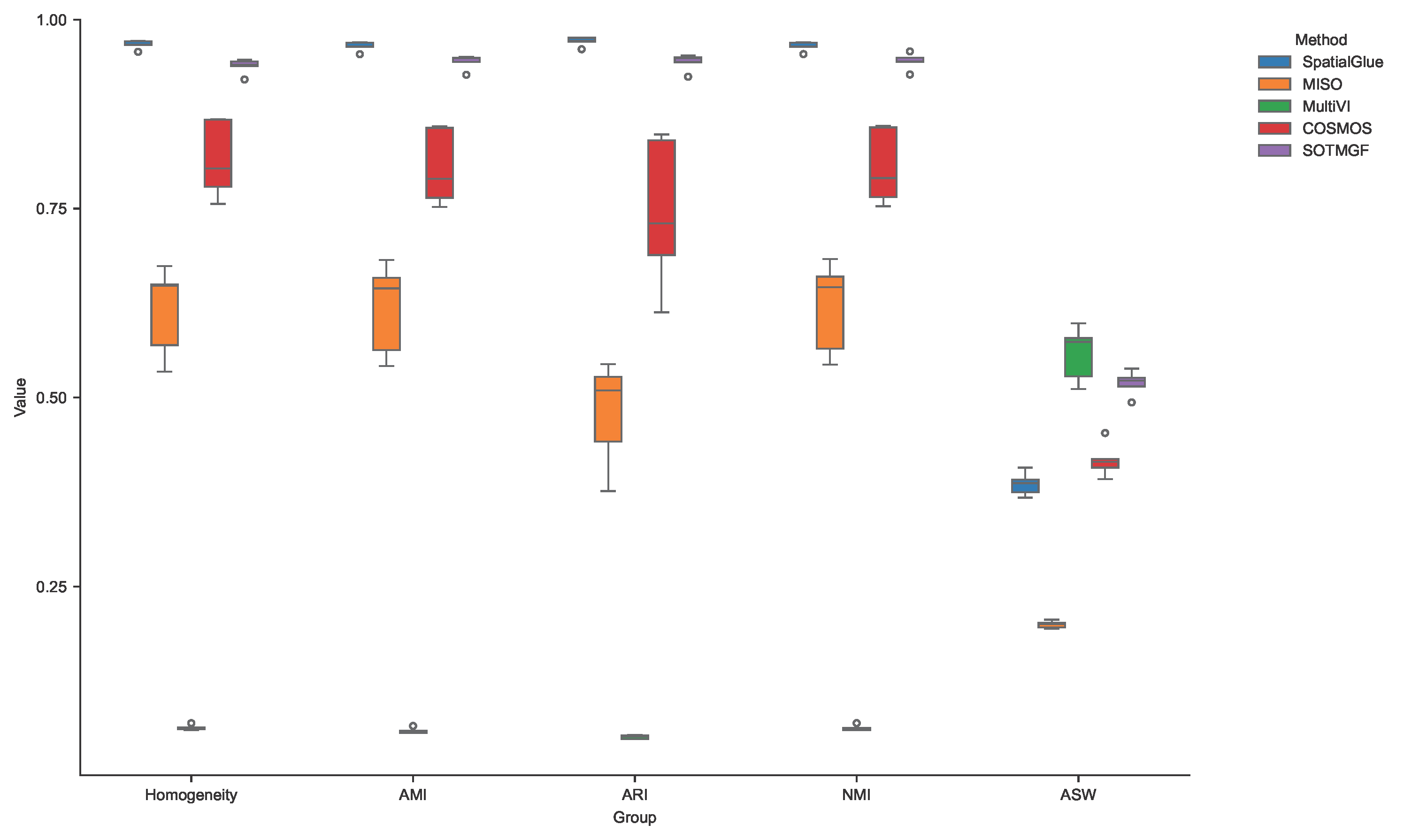


**Figure S1.**

**A)** Spatial domain identification of the simulated dataset in dual-modality including spatial transcriptome and spatial proteome. **B)** Comparison boxplots of spatial domain metrics in five datasets containing two spatial omics.


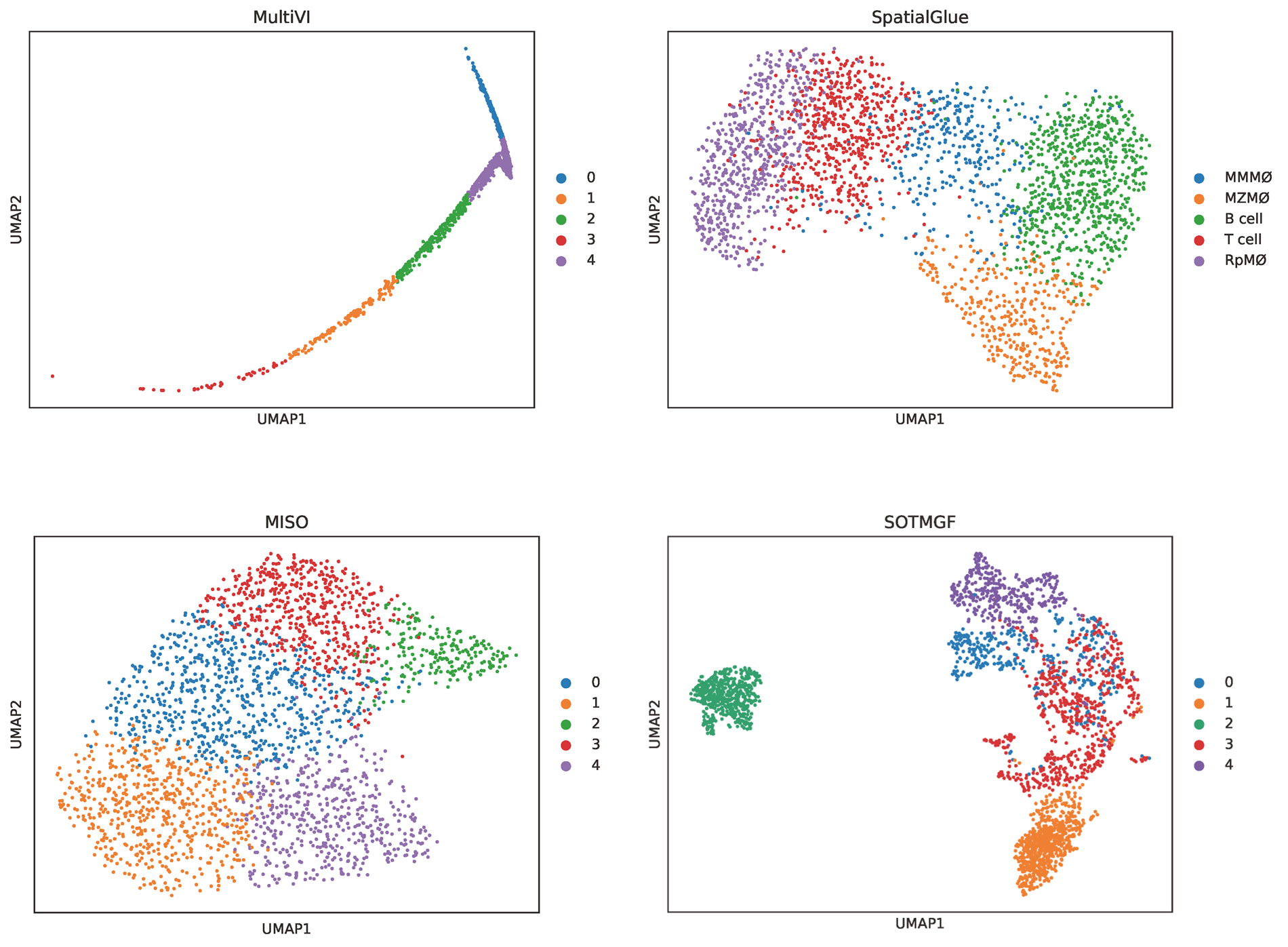


**Figure S2.**

Scatter plot of the two-dimensional UMAP extracted from latent features with the joint analysis of SP and ST by MultiVI, SpatialGlue, MISO and SOTMGF.
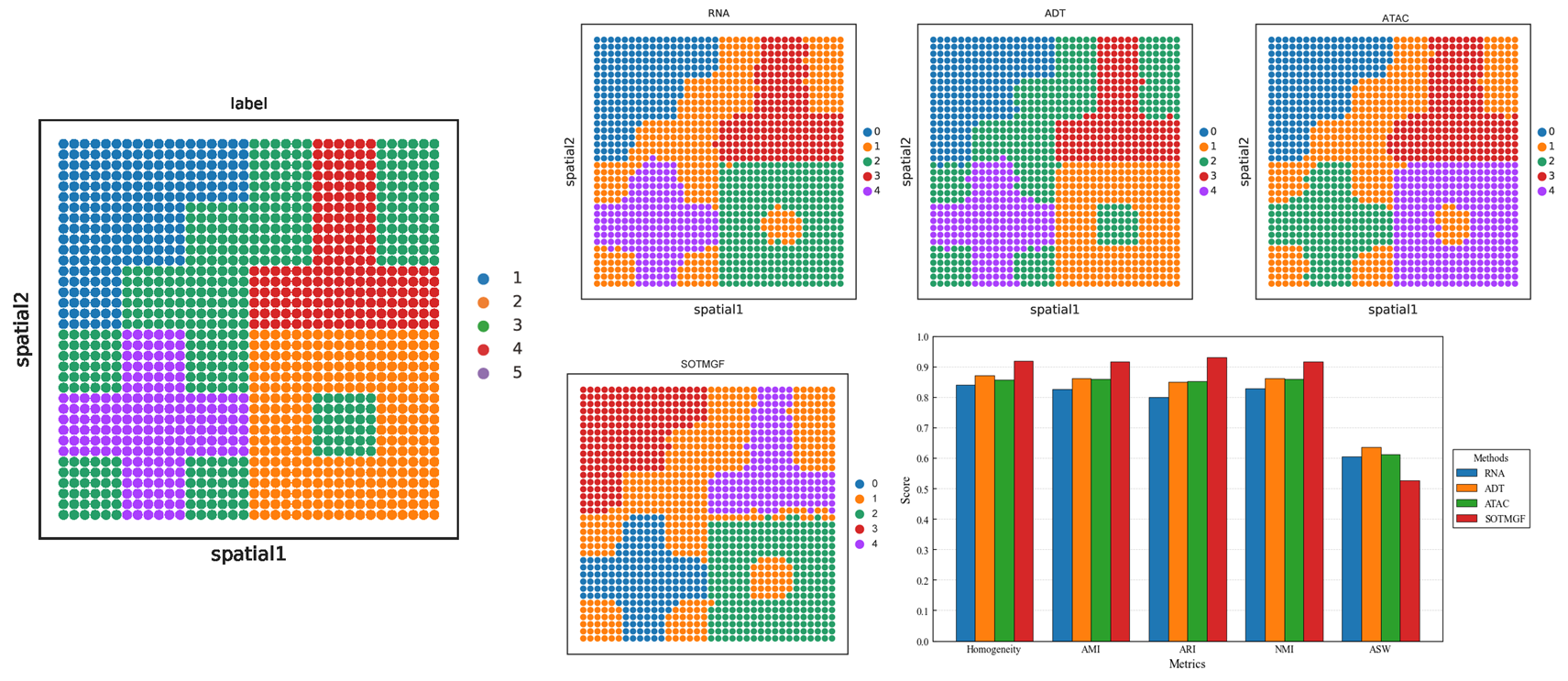


**Figure S3.**

**A)** Spatial domain identification of the simulated dataset in triplet-modality including spatial transcriptome, spatial proteome and spatial ATAC. **B)** Comparison histogram of spatial domain metrics identified in spatial map RNA, protein and ATAC data


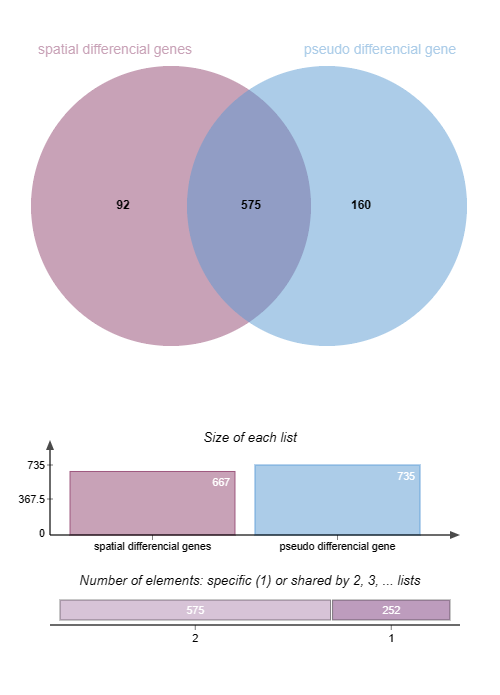


**Figure S4.**

Venn diagram showing spatially differentiated gene overlap across original and denoised gene expression, i.e., spatially dark genes.


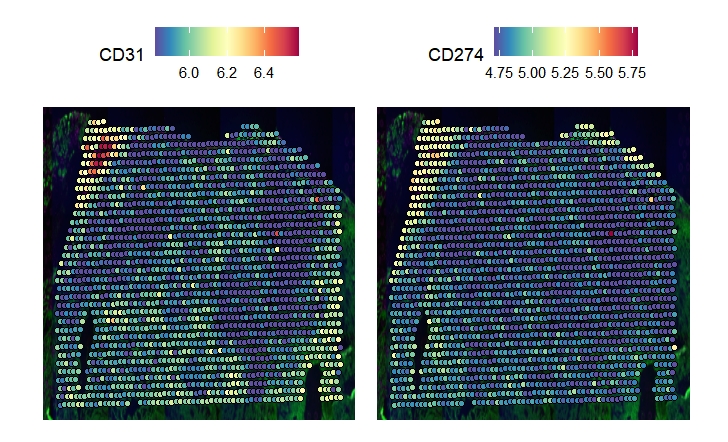

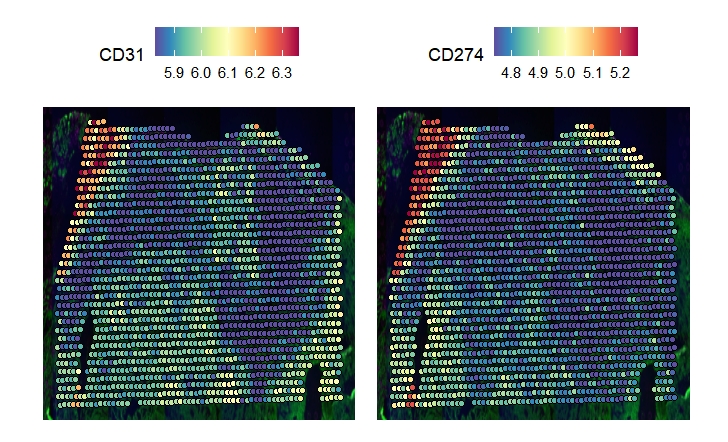


**Figure S5**

Raw and denoised spatial expression profiles of dark proteins.


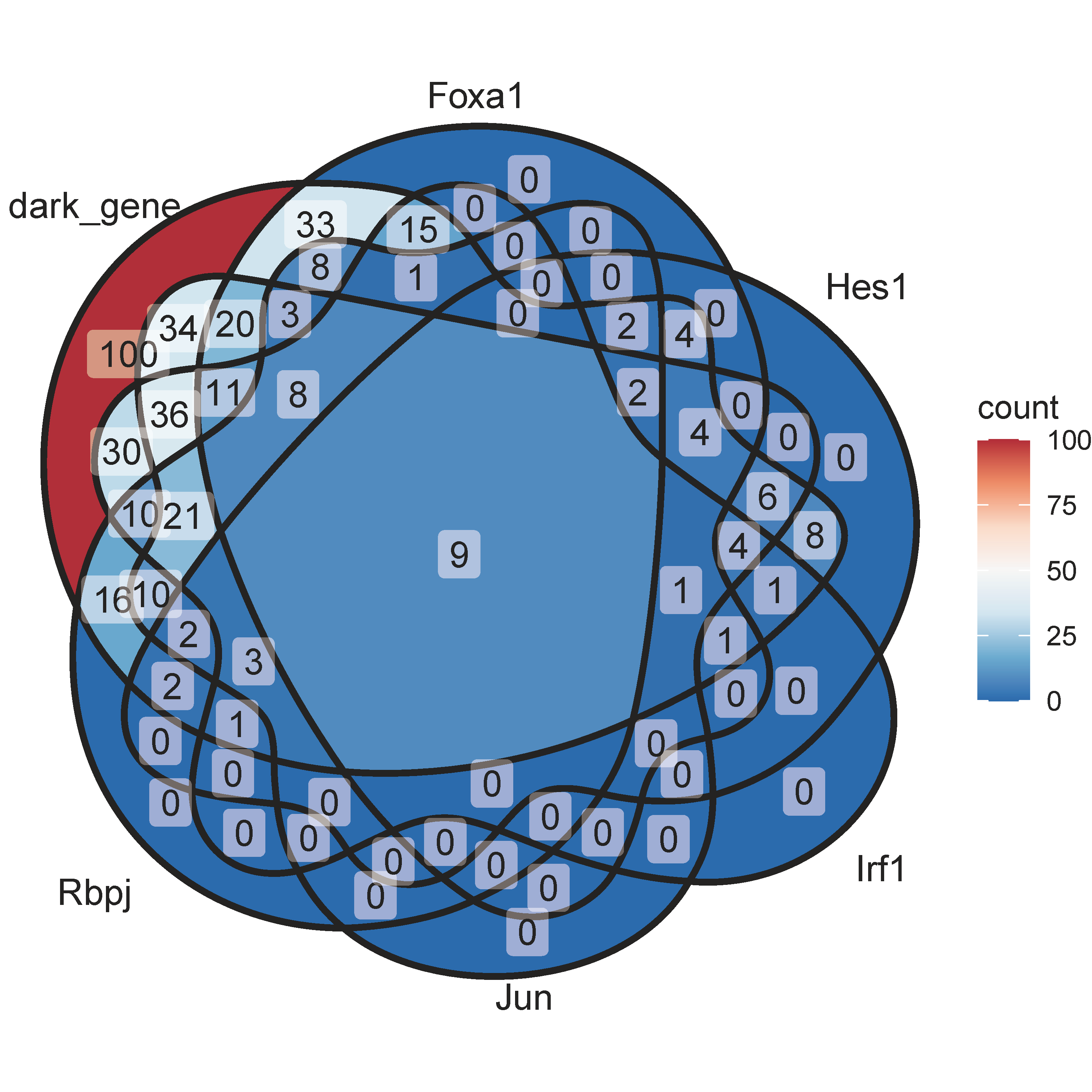


**Figure S6**

Predicted upstream transcription factors (TFs) regulating SOTMGF-derived SDGs. The top five TFs (Hes1, Rbpj, Irf1, Foxa1, and Jun) collectively regulate 75.37% of identified SDGs.


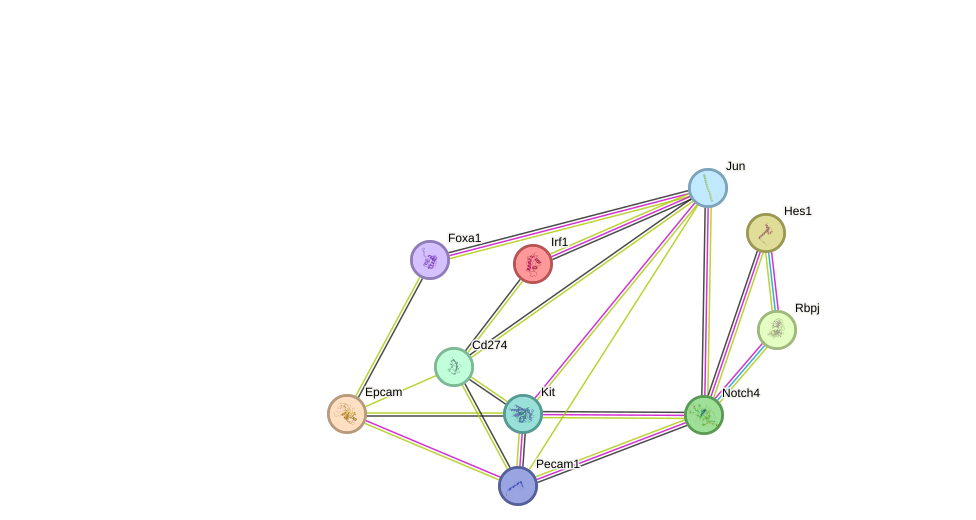


**Figure S7**

Protein-protein interaction networks between the top five upstream transcription factors and two SDPs.
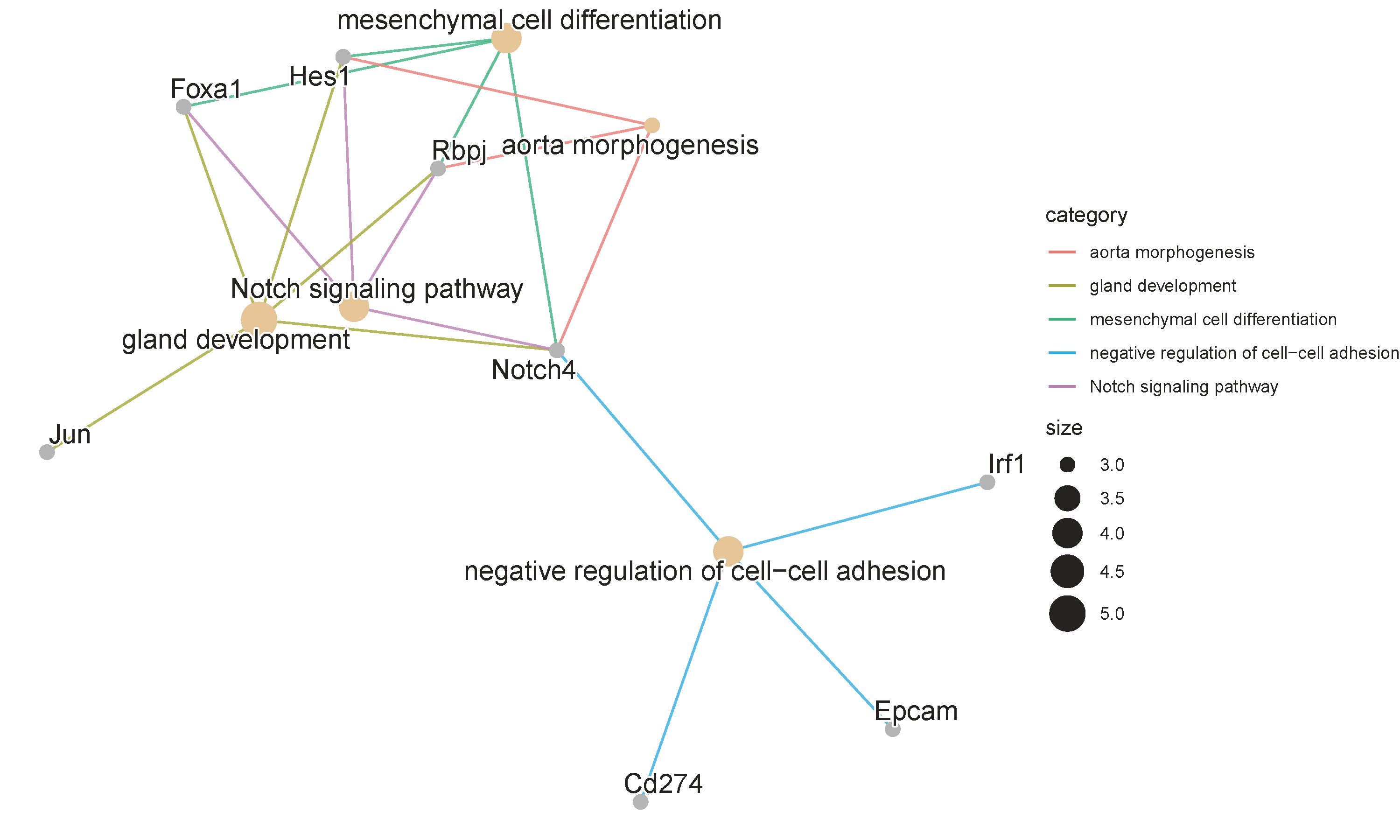

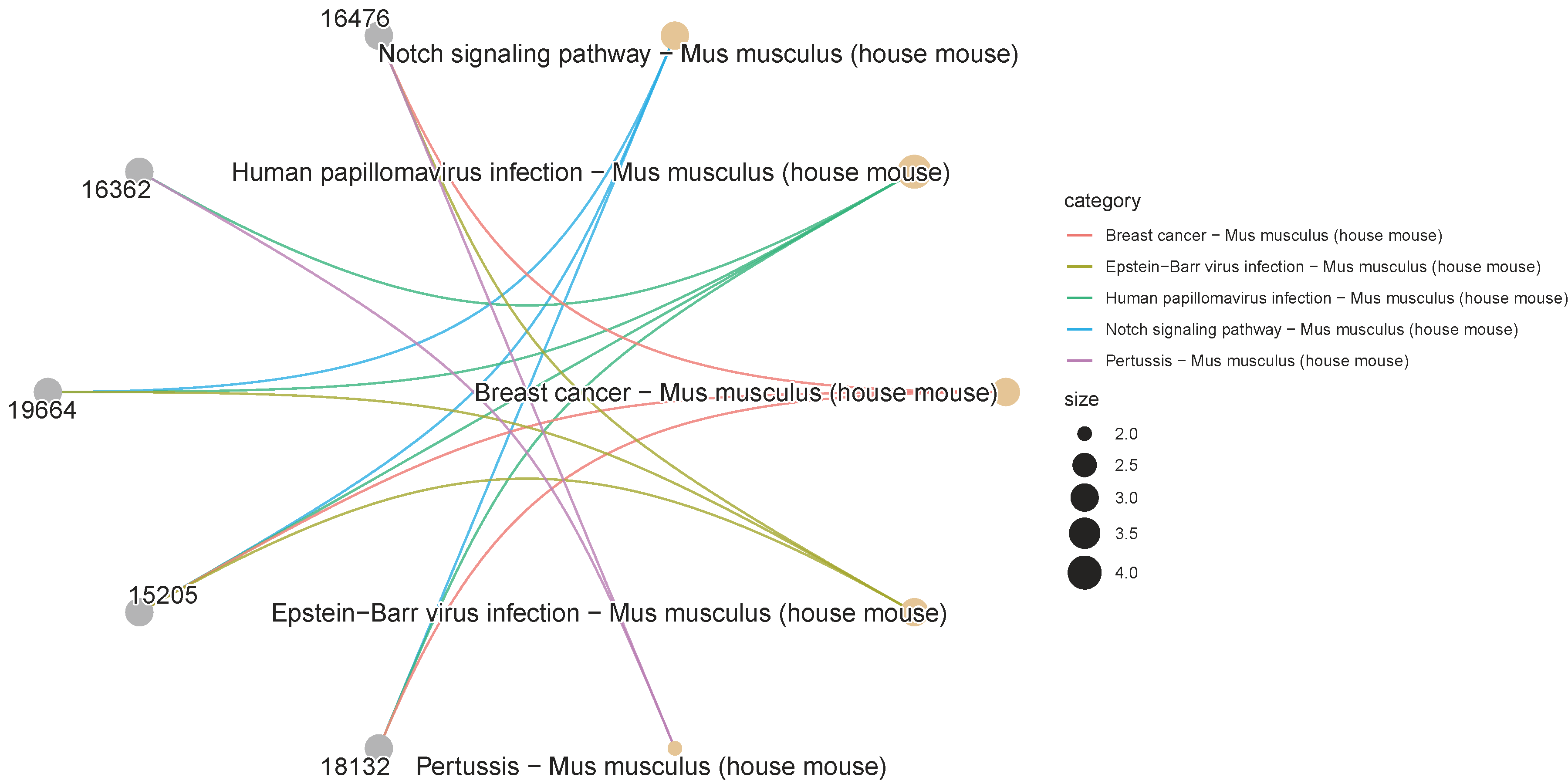


**Figure S8**

**A)** Association network diagram of enriched SDPs with the set of GO functions/pathways they are involved in. **B)** Association network diagram of enriched SDPs with the set of KEGG functions/pathways they are involved in.


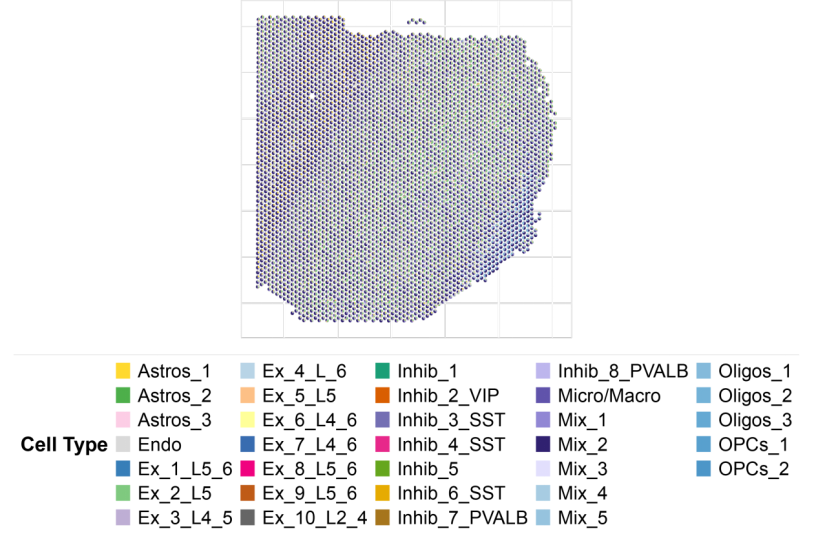

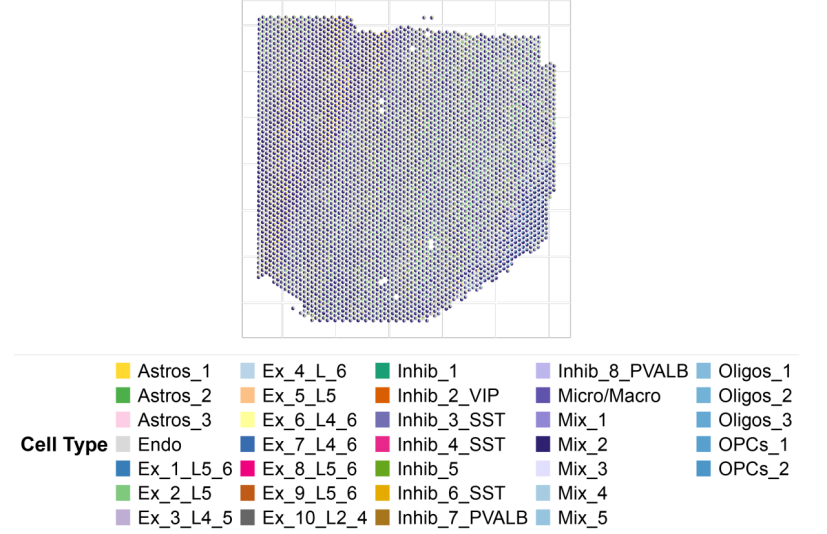


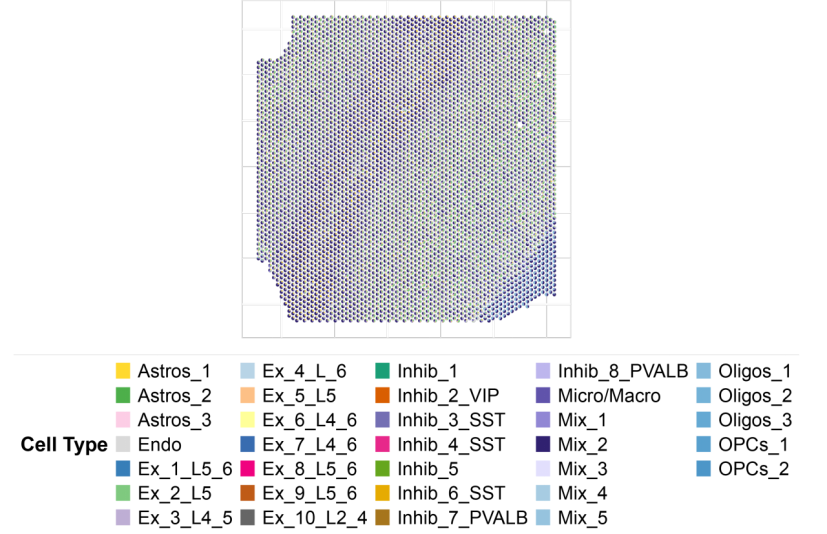

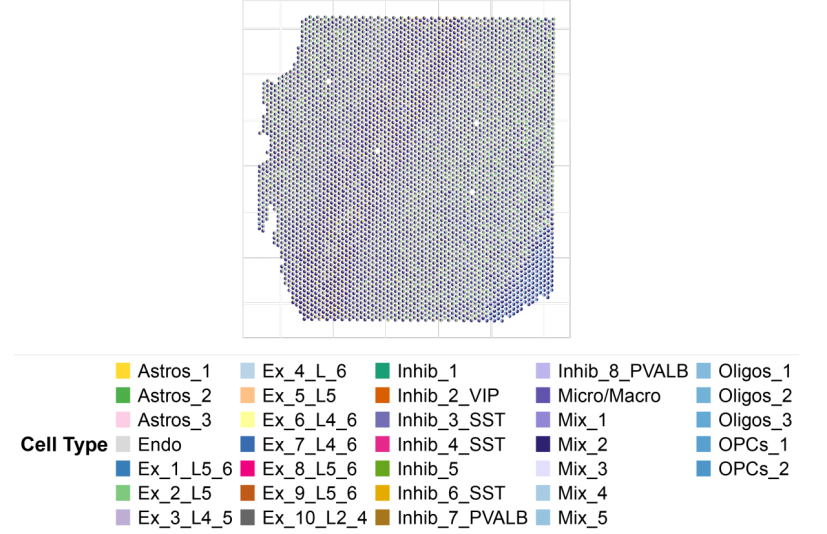


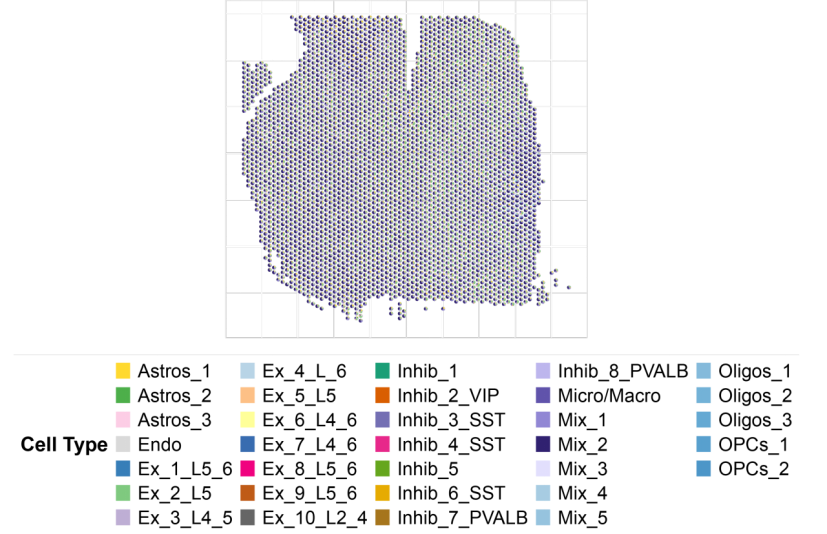

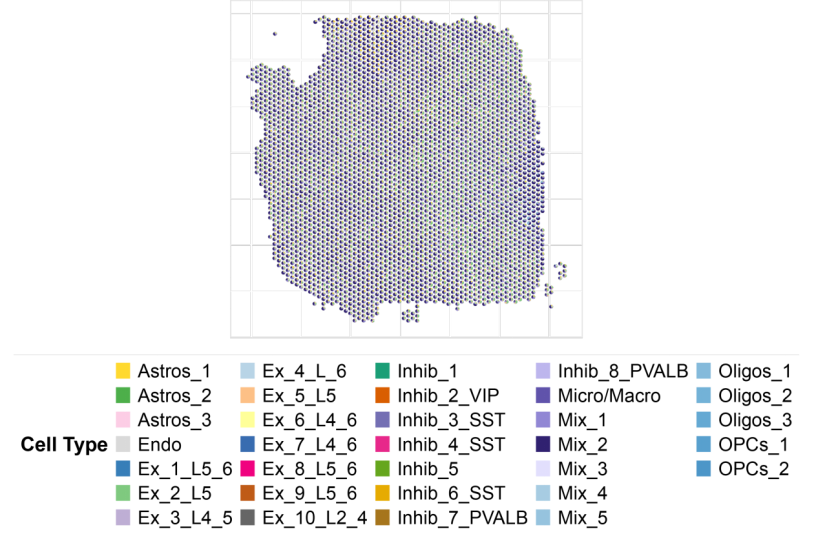


**See next page**


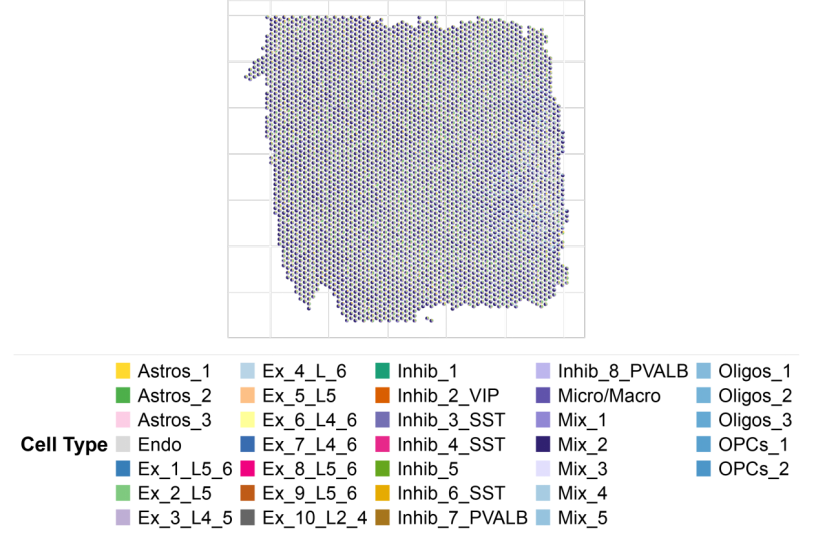

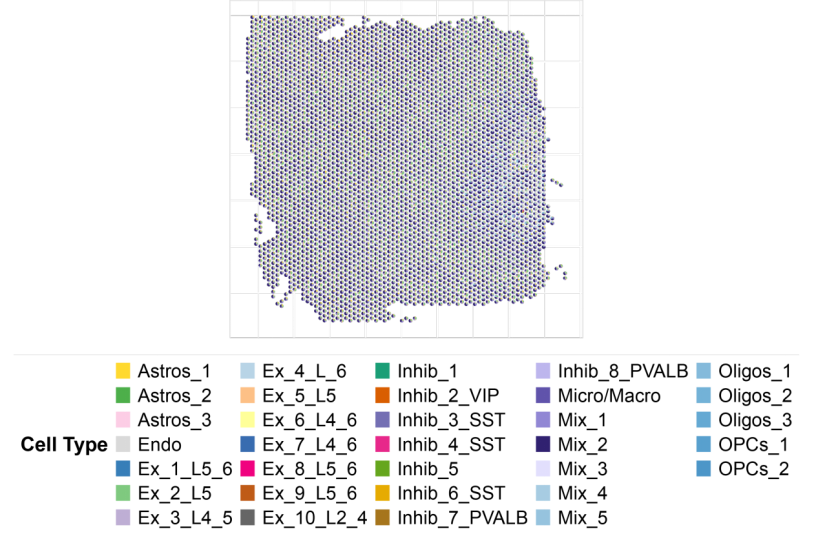


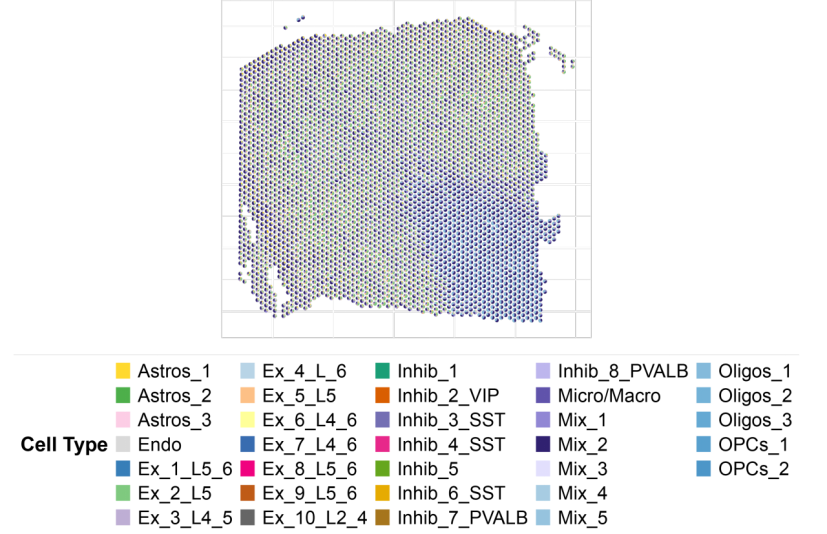

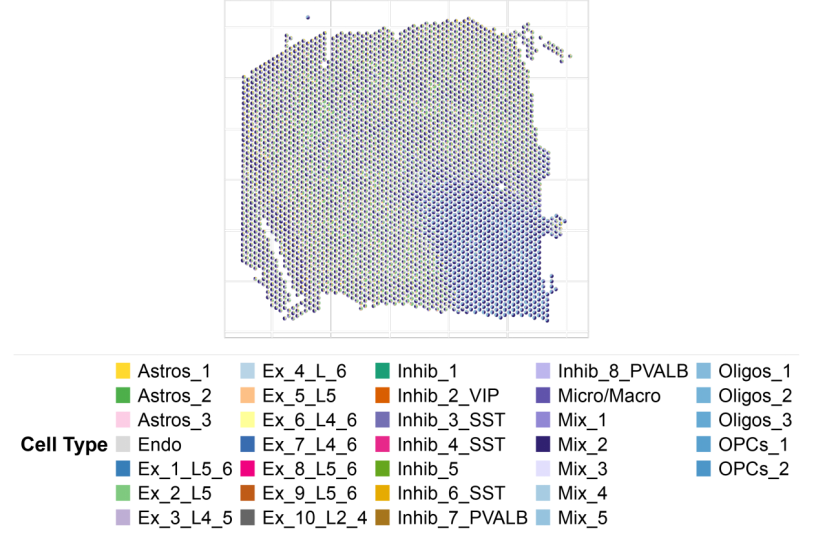


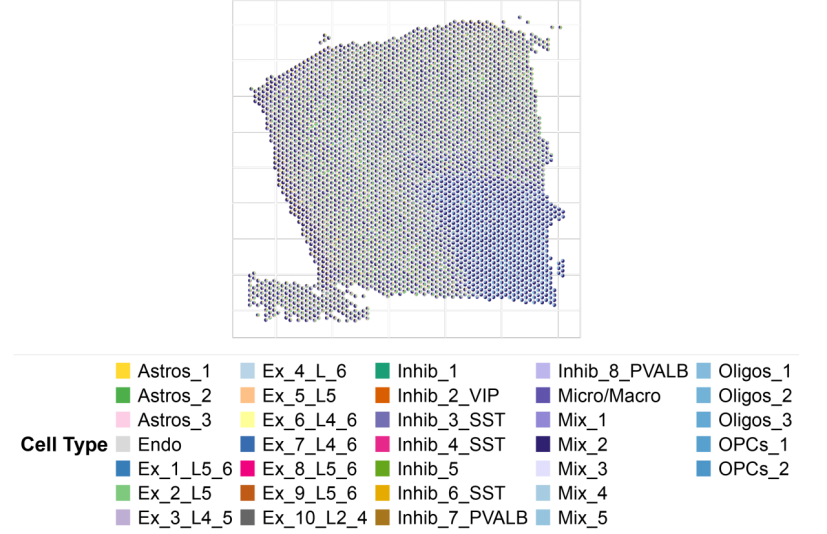

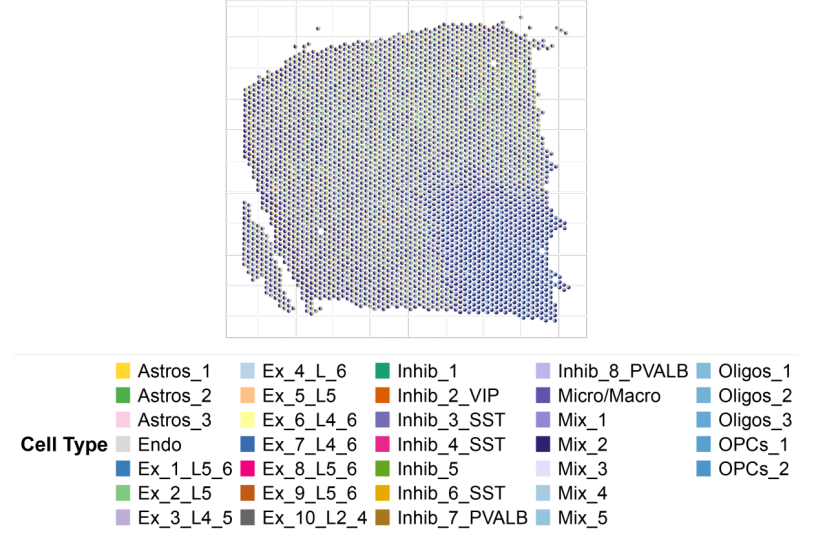


**Figure S9.**

Cell type distribution of 12 slices of DLPFC by CARD deconvolution.


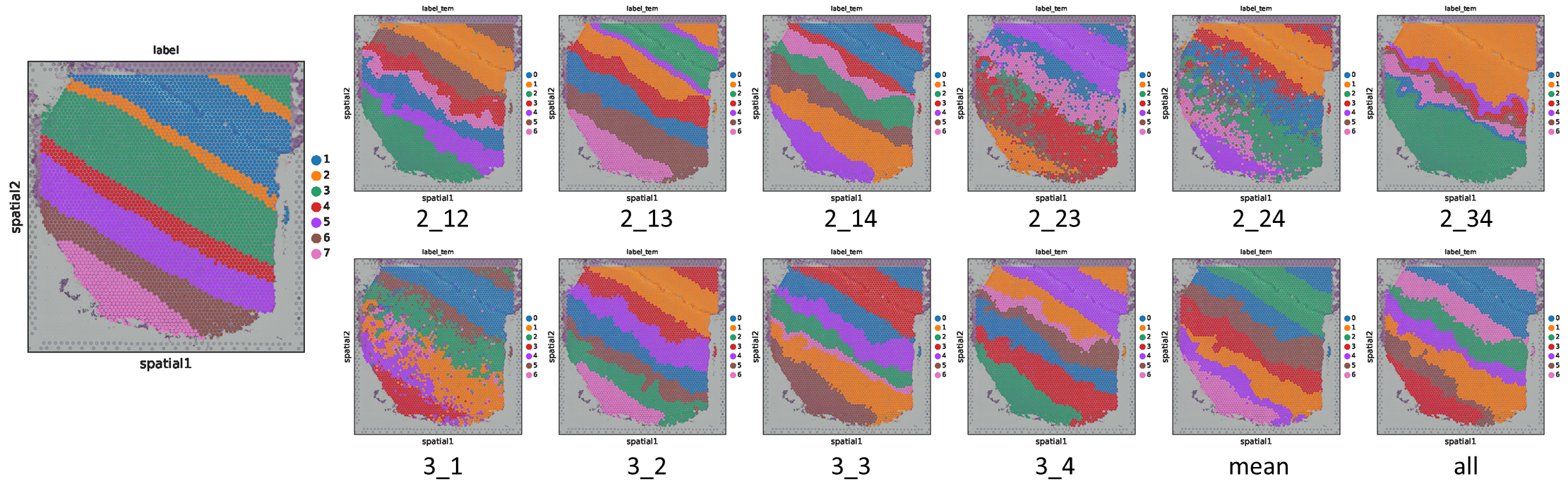

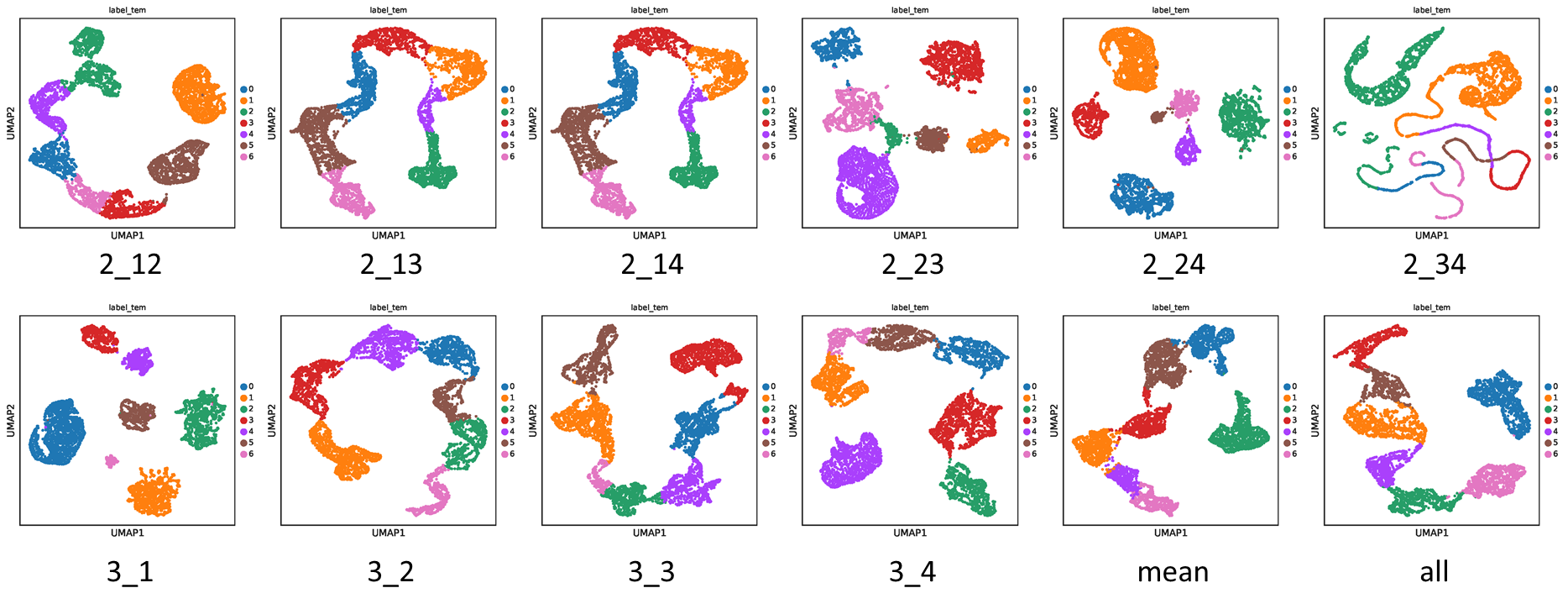


**Figure S10.**

Clustering results and UMAP plots of replaced the fusion mechanism with simpler baselines and ablation studies to assess the contribution of each view.


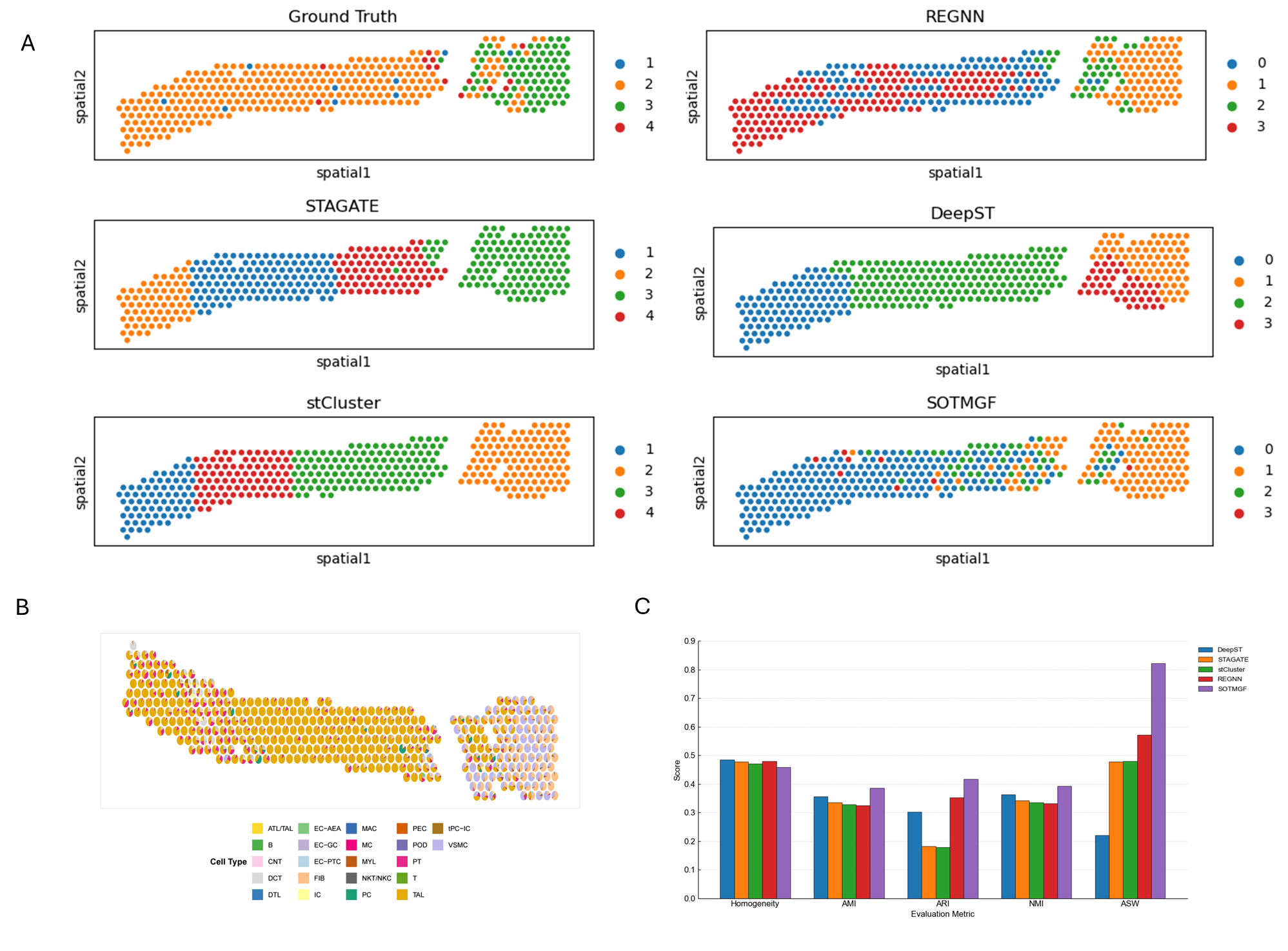


**Figure S11.**

**A)** Visualization of results from computational methods on a representative CKD sample. The gold standard annotations and calculated results of the computational methods are mapped to the original locations of CKD sample V10S14-085_XY04_21-0057. **B)** Cell type distribution of CKD sample V10S14-085_XY04_21-0057 by CARD deconvolution. **C)** Comparison histogram of spatial domain metrics identified in CKD sample V10S14-085_XY04_21-0057.


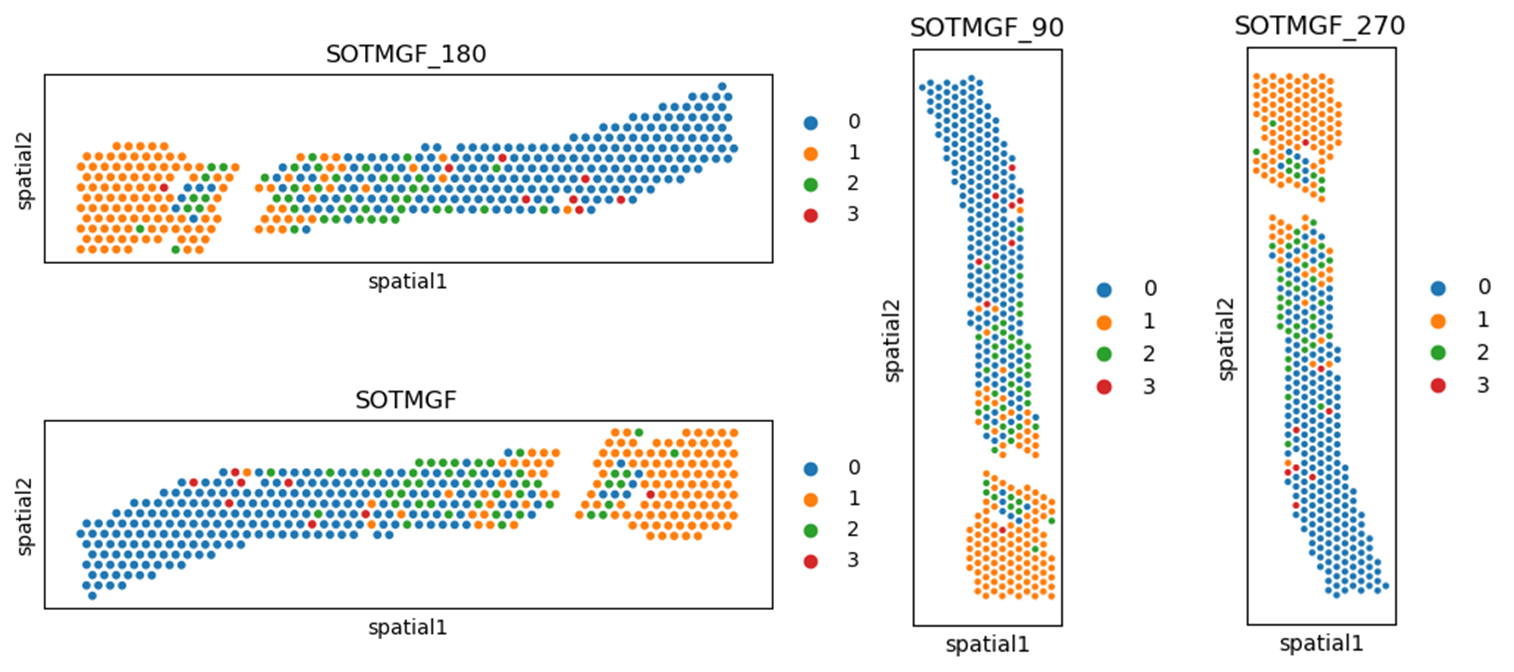


**Figure R12.**

Visualization results of spatial domain identification on the V10S14-085_XY04_21-0057 dataset under different rotation angles.


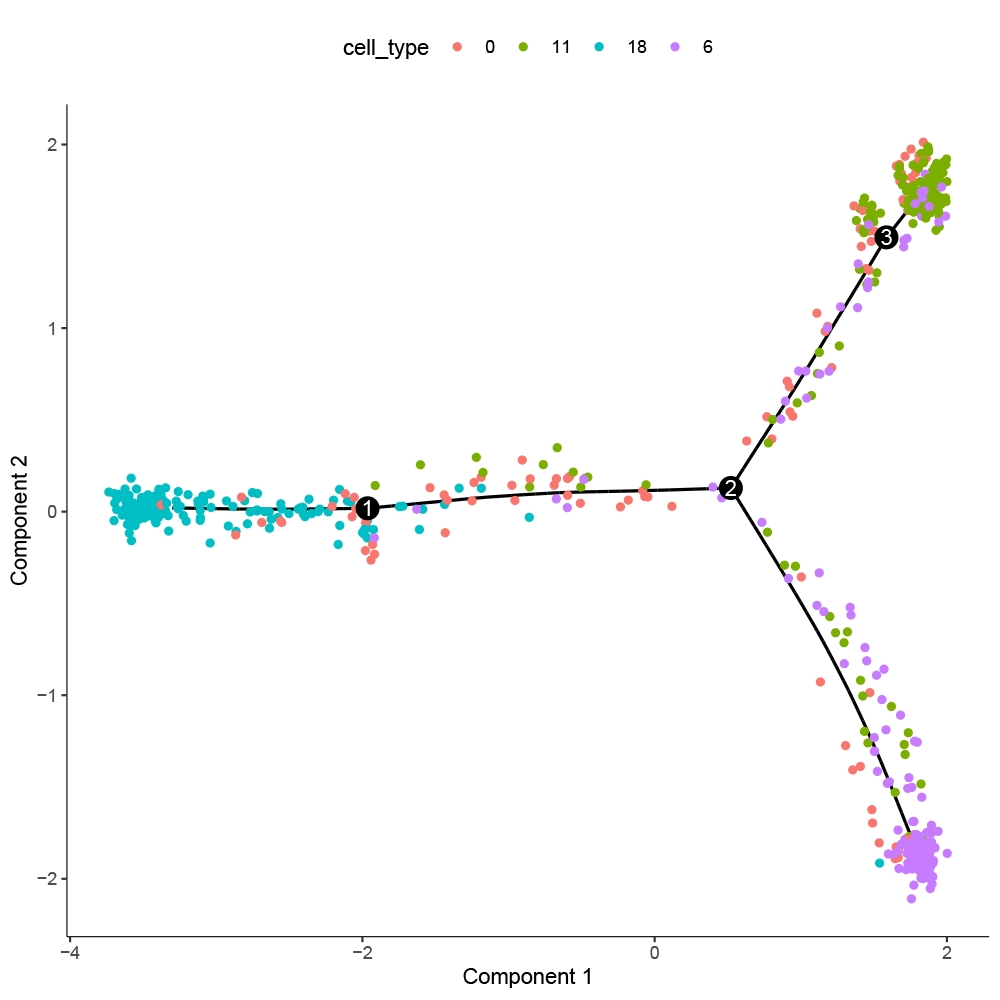

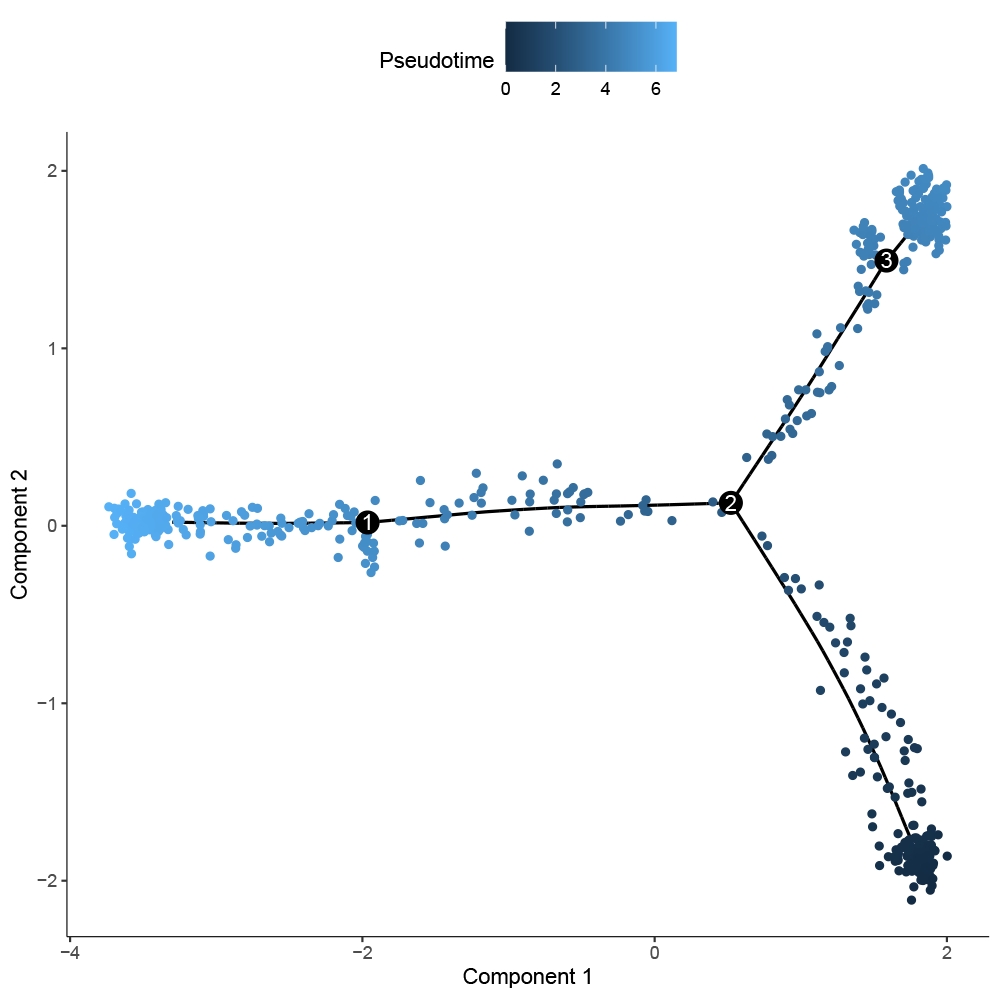


**Figure S13.**

Distribution of cells in each cluster on the cell_type and pseudo-time trajectory.


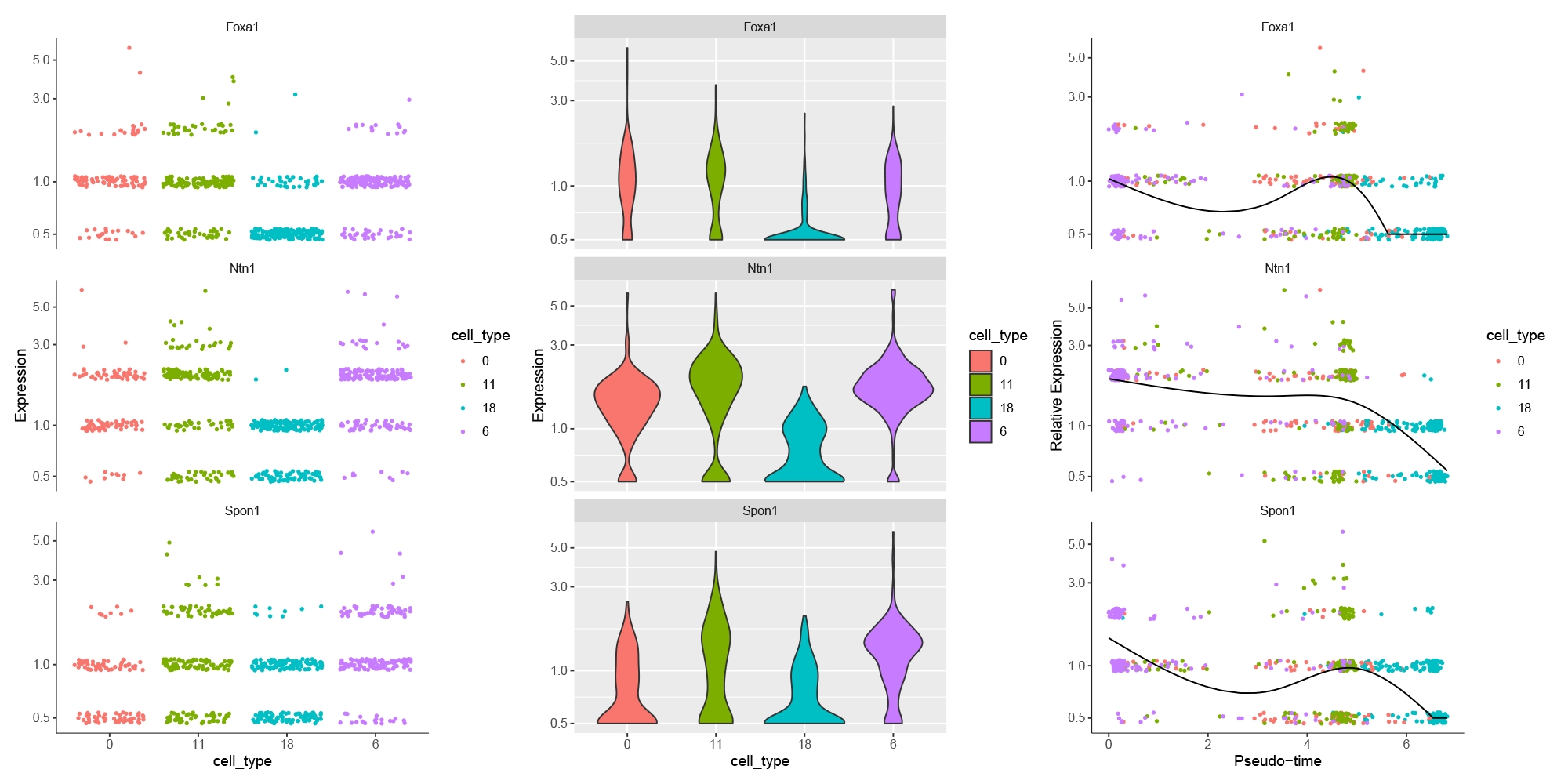


**Figure S14.**

Pseudo-time-dependent changes in the expression levels of Cnpy1, En2, and Pax5. Each color indicates one cluster.


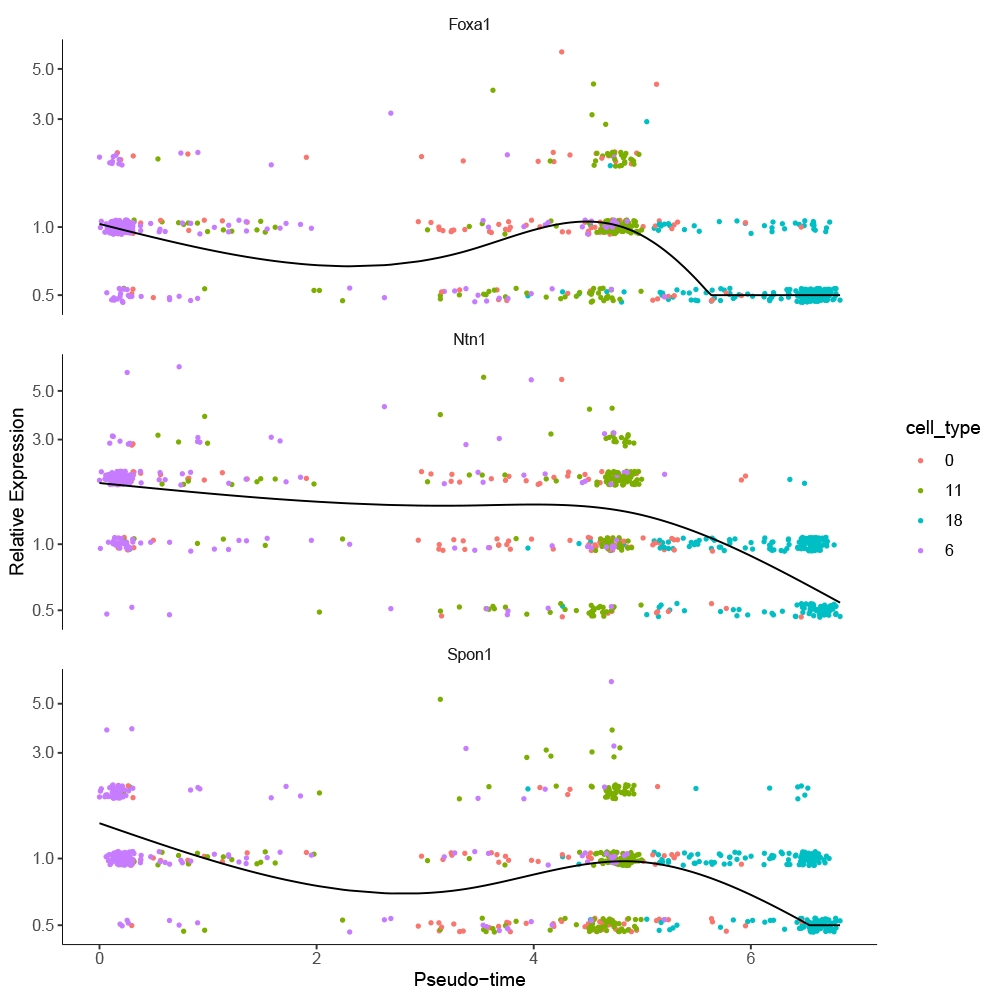

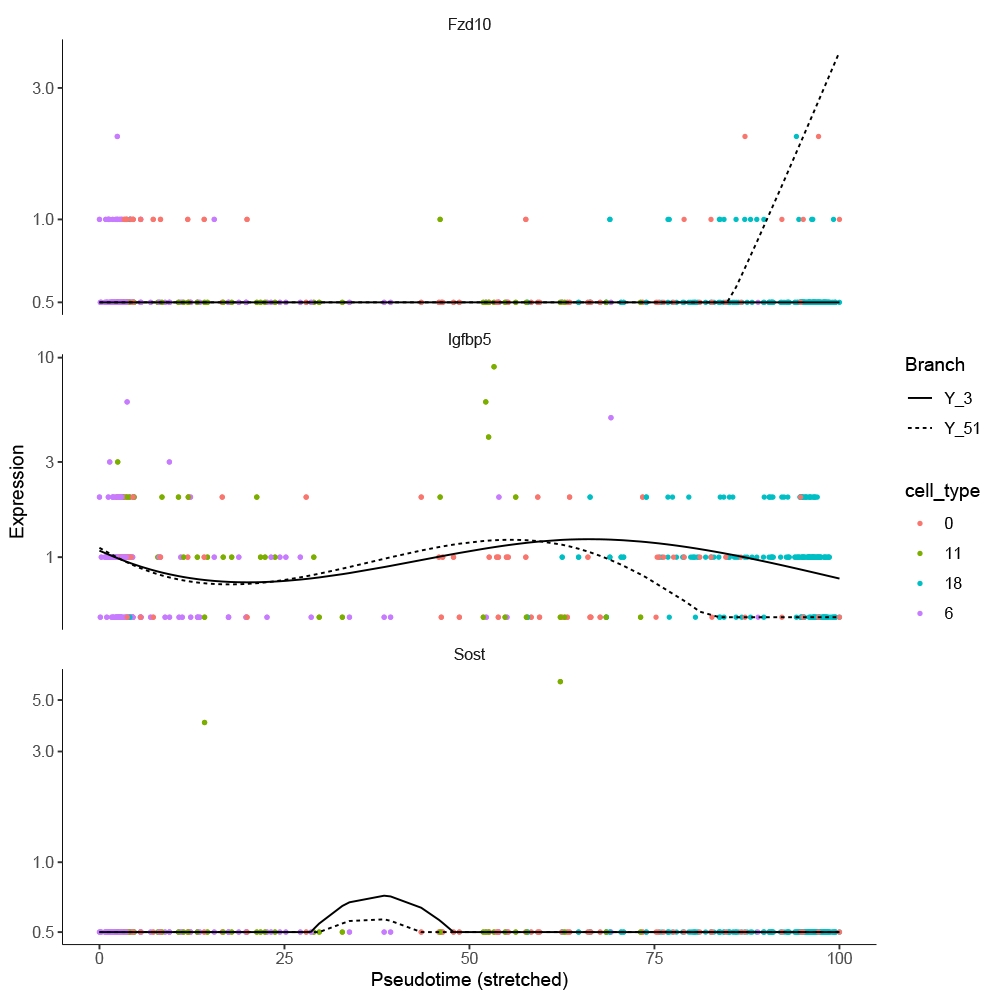


**Figure S15.**

Distribution of gene expression by BEAM analysis.


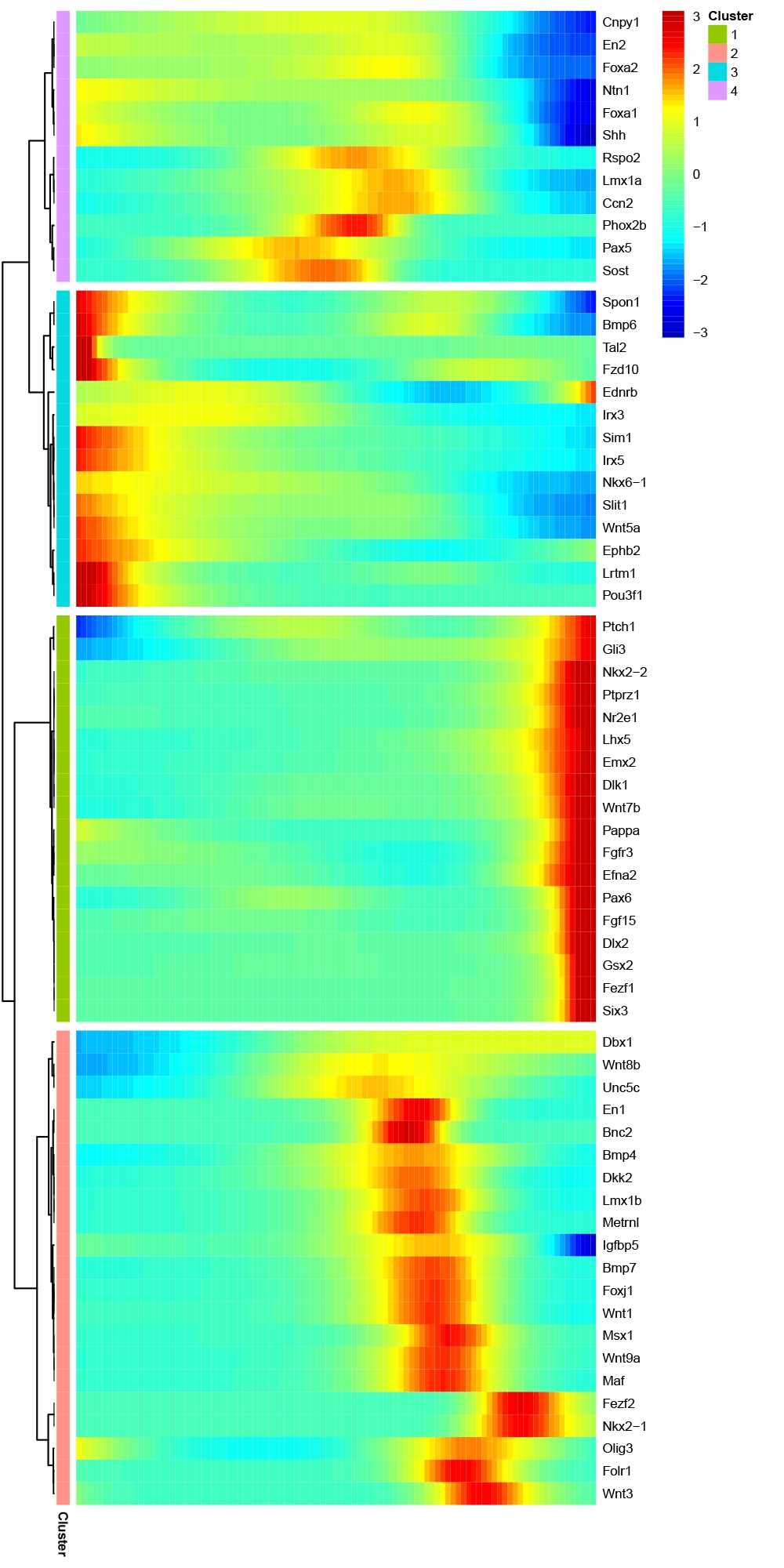

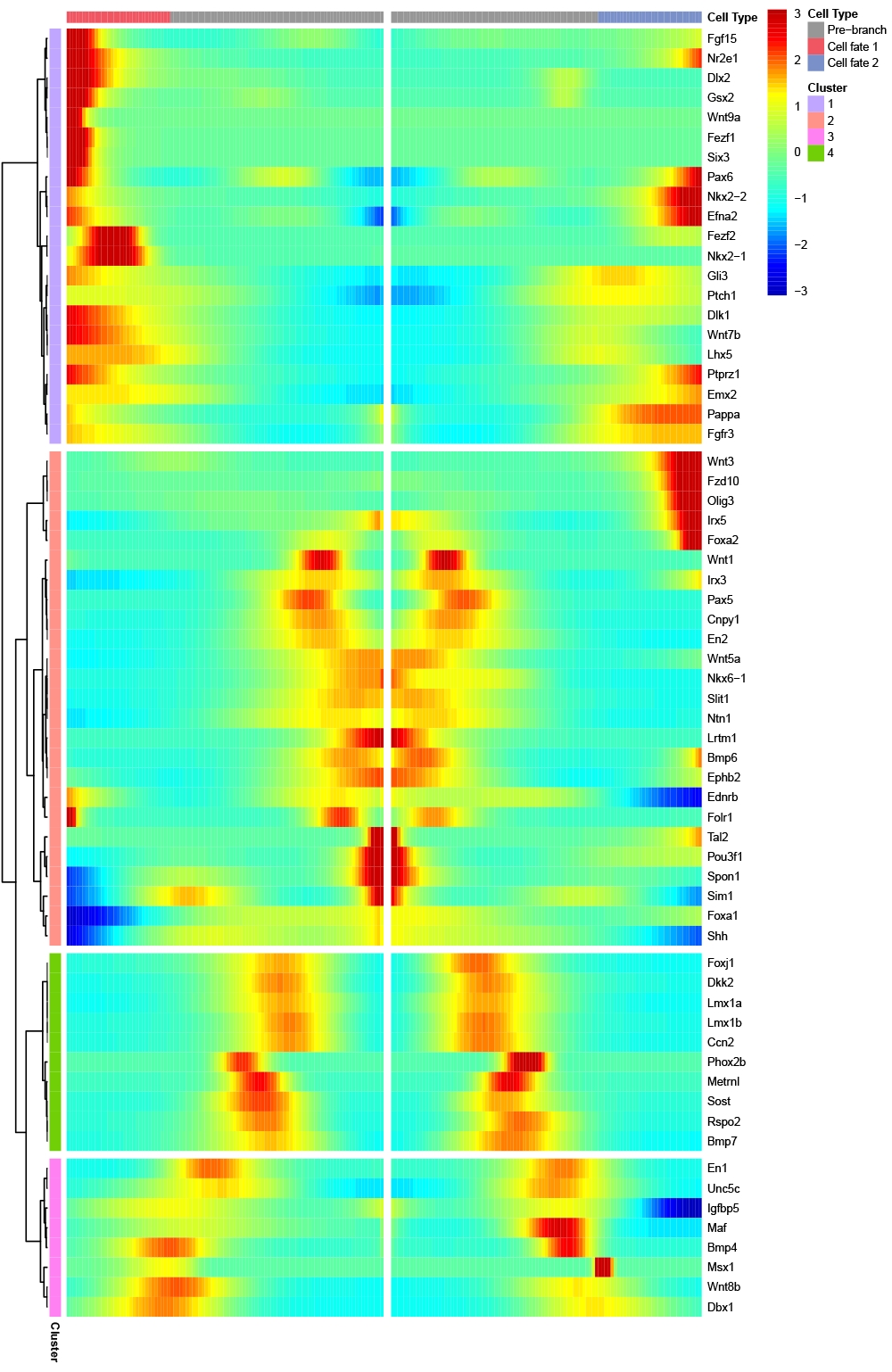


**Figure S16.**

Heatmap of Gene Expression Analyzed by BEAM analysis.


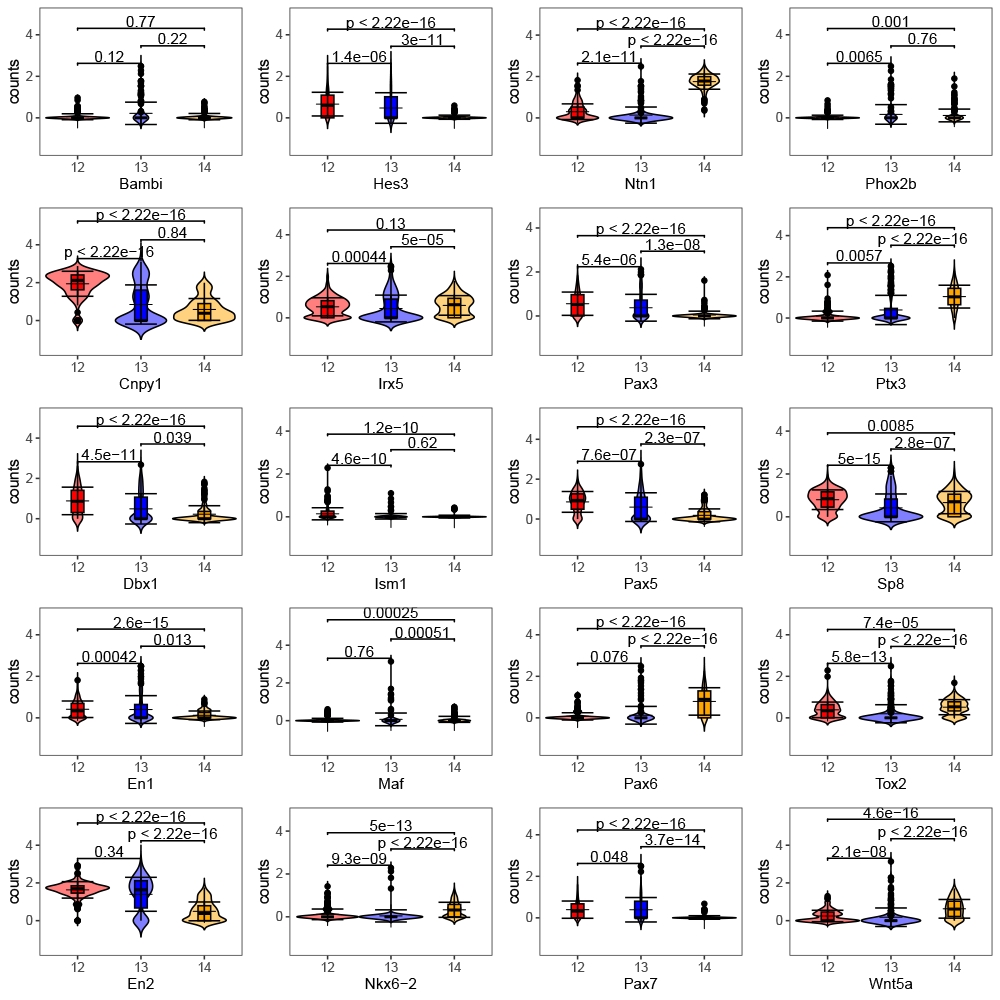


**Figure S17.**

Differences in the distribution of branching-related genes in clusters 12,13,14.


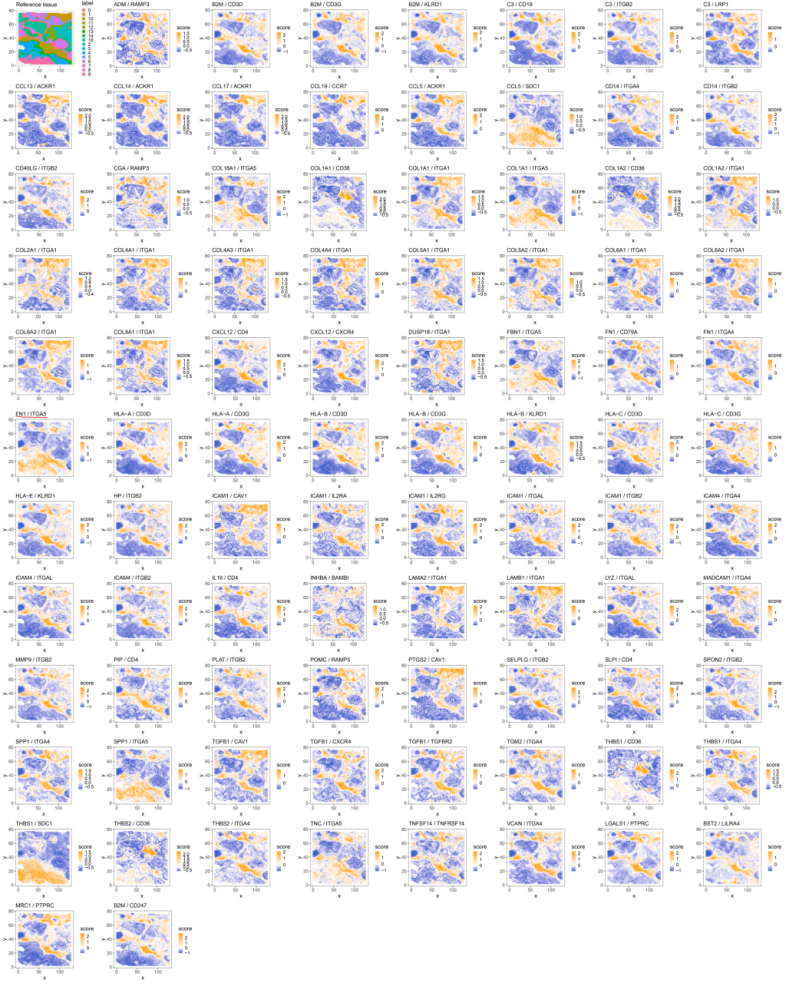


**Figure S18.**

Overview of the 70 identified interactions in the IDC dataset represented by the spatial rendering of their gene signature score.


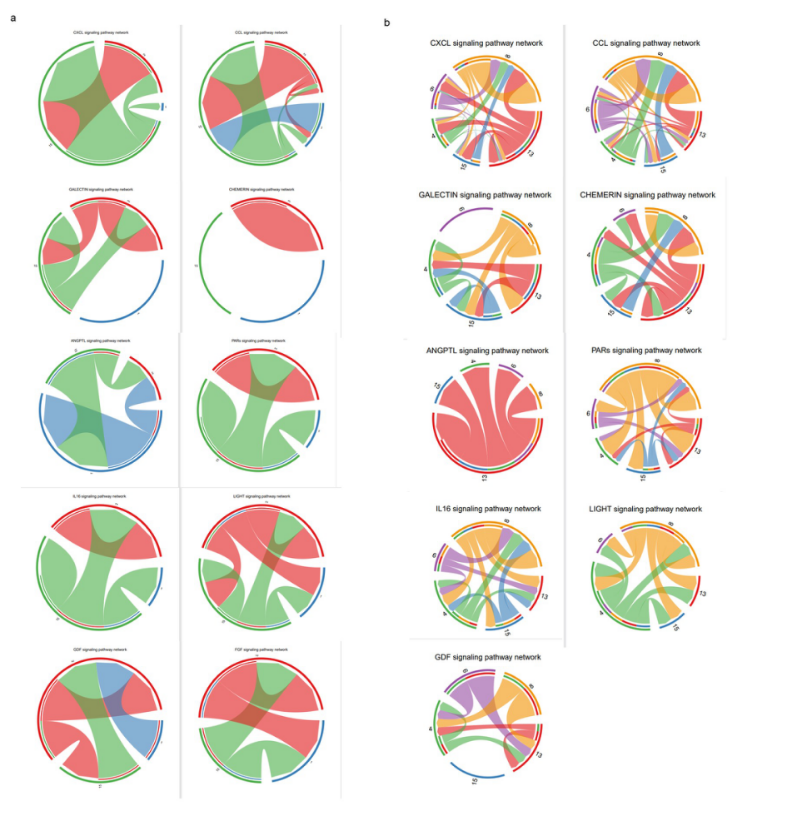


**Figure S19.**

**a** String diagram of the ten signalling pathways identified in clusters 0, 1, 14 signalling between these regions. **b** String diagram of the nine signalling pathways identified in clusters 4, 6, 8, 13, 15 signalling between these regions.





**Figure S20.** Modality weights coefficient explaining the importance of different modalities to each cluster in the mouse brain dataset.

**Table S1.**

GO enrichment analysis of transcription factors.

| ID | Description | p.adjust |
| --- | --- | --- |
| GO:0048732 | gland development | 5.84E-05 |
| GO:0035909 | aorta morphogenesis | 5.84E-05 |
| GO:0007219 | Notch signaling pathway | 5.84E-05 |
| GO:0022408 | negative regulation of cell-cell adhesion | 0.000106515 |
| GO:0048762 | mesenchymal cell differentiation | 0.000114259 |
| GO:0048863 | stem cell differentiation | 0.000124808 |
| GO:0042509 | regulation of tyrosine phosphorylation of STAT protein | 0.000124808 |
| GO:0035904 | aorta development | 0.000124808 |
| GO:0007260 | tyrosine phosphorylation of STAT protein | 0.000124808 |
| GO:0060485 | mesenchyme development | 0.00013292 |
| GO:0048844 | artery morphogenesis | 0.000143556 |
| GO:0003151 | outflow tract morphogenesis | 0.000143556 |
| GO:0007162 | negative regulation of cell adhesion | 0.000143556 |
| GO:0060562 | epithelial tube morphogenesis | 0.000236014 |
| GO:0045446 | endothelial cell differentiation | 0.000294029 |
| GO:0060840 | artery development | 0.000311444 |
| GO:0007159 | leukocyte cell-cell adhesion | 0.000311444 |
| GO:0001886 | endothelial cell morphogenesis | 0.000368517 |
| GO:0048505 | regulation of timing of cell differentiation | 0.000368517 |
| GO:0017053 | transcription repressor complex | 0.008292155 |
| GO:0061629 | RNA polymerase II-specific DNA-binding transcription factor binding | 0.000102912 |
| GO:0001227 | DNA-binding transcription repressor activity, RNA polymerase II-specific | 0.025740046 |
| GO:0035497 | cAMP response element binding | 0.025740046 |
| GO:0001217 | DNA-binding transcription repressor activity | 0.025740046 |
| GO:0008432 | JUN kinase binding | 0.027714262 |
| GO:0071837 | HMG box domain binding | 0.027714262 |
| GO:0005112 | Notch binding | 0.027714262 |
| GO:0070412 | R-SMAD binding | 0.027714262 |

**Table S2.**

KEGG enrichment analysis of spatial dark proteins and transcription factors.

| ID | Description | p.adjust |
| --- | --- | --- |
| mmu04330 | Notch signaling pathway - Mus musculus (house mouse) | 0.00056997 |
| mmu05165 | Human papillomavirus infection - Mus musculus (house mouse) | 0.001873324 |
| mmu05224 | Breast cancer - Mus musculus (house mouse) | 0.002417551 |
| mmu05169 | Epstein-Barr virus infection - Mus musculus (house mouse) | 0.006644861 |
| mmu05133 | Pertussis - Mus musculus (house mouse) | 0.014735382 |
| mmu04658 | Th1 and Th2 cell differentiation - Mus musculus (house mouse) | 0.014735382 |
| mmu05235 | PD-L1 expression and PD-1 checkpoint pathway in cancer - Mus musculus (house mouse) | 0.014735382 |
| mmu01522 | Endocrine resistance - Mus musculus (house mouse) | 0.014735382 |
| mmu04625 | C-type lectin receptor signaling pathway - Mus musculus (house mouse) | 0.018858721 |
| mmu04668 | TNF signaling pathway - Mus musculus (house mouse) | 0.018858721 |
| mmu05418 | Fluid shear stress and atherosclerosis - Mus musculus (house mouse) | 0.026738872 |
| mmu04514 | Cell adhesion molecules - Mus musculus (house mouse) | 0.035514571 |

**References**

1. Y. Long, K. S. Ang, R. Sethi, et al., “Deciphering spatial domains from spatial multi-omics with SpatialGlue,” *Nature Methods* 21 , no. 9 (2024) : 1658–1667 , https://doi.org/10.1038/s41592-024-02316-4.

2. K. Coleman, A. Schroeder, M. Loth, et al., “Resolving tissue complexity by multimodal spatial omics modeling with MISO,” *Nature Methods* 22 , no. 3 (2025) : 530–538 , https://doi.org/10.1038/s41592-024-02574-2.

3. T. Ashuach, M. I. Gabitto, R. V. Koodli, G.-A. Saldi, M. I. Jordan, N. Yosef, “MultiVI: deep generative model for the integration of multimodal data,” *Nature Methods* 20 , no. 8 (2023) : 1222–1231 , https://doi.org/10.1038/s41592-023-01909-9.

4. H. Ren, B. L. Walker, Z. Cang, Q. Nie, “Identifying multicellular spatiotemporal organization of cells with SpaceFlow,” *Nature Communications* 13 , no. 1 (2022) : 4076 , https://doi.org/10.1038/s41467-022-31739-w.

5. C. Xu, X. Jin, S. Wei, et al., “DeepST: identifying spatial domains in spatial transcriptomics by deep learning,” *Nucleic Acids Research* 50 , no. 22 (2022) : e131–e131 , https://doi.org/10.1093/nar/gkac901.

6. F. A. Wolf, P. Angerer, F. J. Theis, “SCANPY: large-scale single-cell gene expression data analysis,” *Genome Biology* 19 , no. 1 (2018) : 15 , https://doi.org/10.1186/s13059-017-1382-0.

7. Y. Zong, T. Yu, X. Wang, Y. Wang, Z. Hu, Y. Li, *conST: an interpretable multi-modal contrastive learning framework for spatial transcriptomics*, Bioinformatics 2022.

8. T. Wang, H. Shu, J. Hu, et al., “Accurately deciphering spatial domains for spatially resolved transcriptomics with stCluster,” *Briefings in Bioinformatics* 25 , no. 4 (2024) : bbae329 , https://doi.org/10.1093/bib/bbae329.

9. Y. Long, K. S. Ang, M. Li, et al., “Spatially informed clustering, integration, and deconvolution of spatial transcriptomics with GraphST,” *Nature Communications* 14 , no. 1 (2023) : 1155 , https://doi.org/10.1038/s41467-023-36796-3.

10. P. J. Rousseeuw, “Silhouettes: A graphical aid to the interpretation and validation of cluster analysis,” *Journal of Computational and Applied Mathematics* 20 (1987) : 53–65 , https://doi.org/10.1016/0377-0427(87)90125-7.
